# How to identify essential genes from molecular networks?

**DOI:** 10.1186/1752-0509-3-102

**Published:** 2009-10-13

**Authors:** Gabriel del Rio, Dirk Koschützki, Gerardo Coello

**Affiliations:** 1 Department of Biochemistry and Structural Biology, Universidad Nacional Autónoma de México, Instituto de Fisiología Celular, Circuito Exterior s/n ciudad Universitaria, 04510 México D.F., México; 2 Department of Molecular Genetics, Leibniz Institute of Plant Genetics and Crop Plant Research (IPK), Corrensstr. 3, 06466 Gatersleben, Germany; 3 Department of Computer and Electrical Engineering, Furtwangen University of Applied Sciences, Robert-Gerwig-Platz 1, 78120 Furtwangen, Germany

## Abstract

**Background:**

The prediction of essential genes from molecular networks is a way to test the understanding of essentiality in the context of what is known about the network. However, the current knowledge on molecular network structures is incomplete yet, and consequently the strategies aimed to predict essential genes are prone to uncertain predictions. We propose that simultaneously evaluating different network structures and different algorithms representing gene essentiality (centrality measures) may identify essential genes in networks in a reliable fashion.

**Results:**

By simultaneously analyzing 16 different centrality measures on 18 different reconstructed metabolic networks for *Saccharomyces cerevisiae*, we show that no single centrality measure identifies essential genes from these networks in a statistically significant way; however, the combination of at least 2 centrality measures achieves a reliable prediction of most but not all of the essential genes. No improvement is achieved in the prediction of essential genes when 3 or 4 centrality measures were combined.

**Conclusion:**

The method reported here describes a reliable procedure to predict essential genes from molecular networks. Our results show that essential genes may be predicted only by combining centrality measures, revealing the complex nature of the function of essential genes.

## Background

Modelling the molecular mechanisms present in living organisms represents an active area of research in biology. Such modelling is appealing to scientists both in applied and basic research areas, because they represent a way to test our basic understanding on how cells are organized. The models also have the potential to accelerate the discovery of new drugs (*e.g*., antibiotics) or to guide the engineering of new organisms that are better at producing desired compounds (*e.g*., vitamins). Different approaches are available to build molecular networks, but in any case these models need to be able to reproduce an observed feature of a biological system. This ability rests on the assumed relationship between a biological observed feature (commonly referred as *phenotype*) and the molecular structure of the cell. We will refer to this relationship, as the *structure-function relationship paradigm*. This paradigm may be stated as follows:

S1. A given cellular phenotype is related to the molecular structure of the cell.

Additionally, the molecular structure of a cell is represented by a *molecular network*; that is, the set of molecules and molecule-molecule interactions (*e.g*., genetic relationships, protein-protein interactions).

According to S1, molecules and their interactions have to be known in order to model phenotypes. The advances on DNA manipulation techniques have allowed scientists to systematically identify all the genes from different organisms and consequently, genes became the first set of molecules to model phenotypes. It is important to note that although genes represent only one of the many different types of molecules in biological systems (*e.g*., proteins, ions, carbohydrates, lipids), genetic relationships on the other hand represent different molecular interactions (*e.g*., protein-protein interactions, protein-DNA interactions). Mapping genetic relationships has the advantage that these are observed by relatively simple experiments such as gene deletion experiments or gene expression studies. For instance, using a culture media without Arginine to grow different gene deletion mutant strains may be used to identify genes related to the biosynthesis of Arginine; alternatively, extracting and comparing the mRNA from a strain cultured with and without Arginine may be used to identify up-regulated gene transcripts in the presence of Arginine, indicating their potential functional relationship. Thus, having access to a full set of genes and their relationships (otherwise, having access to a genetic network) may ultimately render a model to describe a phenotype.

The goal of the present work is to describe a computational procedure to achieve the reliable prediction of essential genes from such genetic networks. To achieve that, we need access to a set of molecular networks and algorithms reproducing some features of essentiality. The need for different sets of networks is justified by the current incompleteness of biological networks.

In previous studies the effective reconstruction of transcriptional regulatory networks (TRN) has been achieved [1,2]. Wagner for instance [1], described an algorithm where parsimonious TRN can be built based on any experimental data source in only n^2 ^steps, being n the number of genes. A parsimonious network uses the lowest number of genetic relationships to connect every pair of genes [1]. However, the algorithm does not attempt to establish the completeness of the network, instead it depends on the available data. Alternatively, the group of Palsson [2] described a procedure for TRN reconstruction as well. To validate the completeness of the TRN, predicted cell growth rates from the metabolic enzymes included in the TRN are compared with observed ones [2]. Although this is a very powerful tool, we argue that these are based on chemicals while genetic networks are based on genes; as we have argued above, information for genes is now readily available and more complete [3]. Other reports have used all the known genetic relationships for yeast to infer the role of genes [4]; however, not all the reported genetic relationships are used in every particular process performed by yeast (*e.g*., sexual reproduction may use a different set of genes and/or genetic relationships than cell division). Hitherto, to the best of our knowledge, the available experimental data and theoretical approaches have not completed the mapping of all the genetic relationships for every cellular phenotype.

On the other hand, the diverse nature of essential genes [5] may explain the limited success of previous attempts to predict them [6-8]. Hence, in the current scenario where there is uncertainty about the completeness of the network it is likely that the algorithms may fail to identify the true nature of essentiality. In order to evaluate the significance of the algorithm used to predict essential genes, it is necessary to vary both the network structure and the algorithm to predict essentiality. In this way, by comparing the significance of the prediction algorithm in different network structures it is possible to find the most significant result among the tested networks.

**Figure 1:**
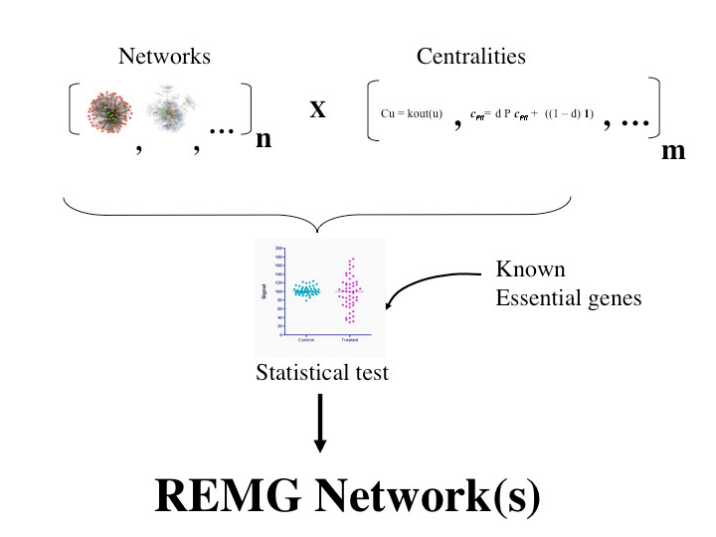
**Reliable prediction of essential genes**. Prediction of essential genes depends on both the quality of the network and the efficacy of the prediction algorithm to reproduce the features of essentiality. To account for these two factors, our method uses a set of **n **networks modelling a biological process, and **m **different centrality measures are applied to these to identify both the network(s) with the largest predictable set of essential genes and the centrality(ies) more effective to identify the essential genes. To determine the reliability of these predictions it is necessary to have access to the set of known essential genes.

Here, we show the first combined analysis of these two aspects to reliably predict the essential genes from networks as follows (see Figure 1). First, according to S1, a phenotype may be represented as a molecular network with *n *genes; formally, a network G is defined by two sets, a set of genes V and a set of relations R, such that:

V(G) = {v1, v2, ... vn} where n is the total number of genes in the network and V(G) is not empty.

R(G) ⊆ V(G) × V(G) such that R(G) is not empty.

Now we define the set of critical genes associated to a phenotype as **c**; thus:



Consequently, we postulate that:

P1. There should be a mathematical operation on G to rank V(G) in a way to obtain c.

It is expected that G is incomplete, thus the ability to identify **c **may be limited as well. In that condition, the best approach to predict **c **is to identify the G that allows for the most complete prediction of set c. This constitutes the limiting condition of P1.

In the present work, we will develop a method based on postulate P1, that is, we will describe a method to achieve the reliable prediction of essential genes from genetic networks. We propose as the mathematical operation described in P1 the calculation of network centralities. A centrality is defined, as a measure that computes the relative importance of network elements (genes) within a network. Therefore, given a centrality measure that satisfies postulate P1, this must include the basic features that determine the essentiality of a gene within the network.

## Methods

### Databases and computer programs

The genetic metabolic networks of *S. cerevisiae *described in this work were based on the KEGG PATHWAY database [9] and the hand-curated model iND750 [10]. Genetic metabolic networks were built by connecting the genes annotated as enzymes through metabolites. That is, two genes coding for metabolic enzymes may be linked if they share a metabolite that is the product of one of the reactions and the substrate for the other. All metabolites were considered here except in the cases indicated (see below). Additionally, a depurated version of KEGG was built and named KEGG2. This version was obtained by eliminating the genes in the KEGG database without an EC classification. In KEGG2, genes with the same EC number and different substrate were forced to accept all substrates found for the given EC number. All the programs used to rebuild and analyze the genetic metabolic networks were coded in Java programming language.

### Directionality of the networks

Enzymatic reactions involved in metabolism could be either reversible or irreversible. We refer to this as the reaction type. However, genetic relationships are not reversible or irreversible as a consequence of the experimental way these are detected (gene deletions or gene transcription profiles). To account for these different ways to represent the relationships of genes, we built genetic metabolic networks assuming the annotated reversibility of the reaction (*e.g*., KEGG-derived networks with the "type" word, *e.g*., KEGGtype network) or assuming all the genetic relationships were reversible (KEGG-derived networks without the "type" word, *e.g*., KEGG or KEGGpath) (see Table 1). In summary, all KEGG-derived networks named without the "type" keyword include reversible genetic relationships (*i.e*., undirected networks).

**Table 1:** Genetic metabolic networks used in this study

**Network name**	**Over-linked metabolites**	**Vertices:Edges**	**Overlap (%)**
**KEGG**	H2O, ATP, ADP, NAD+, NADH, NADP+, NADPH, Oxygen	636:10038	33.85

**KEGGtype**	Ibid	629:6590	26.81

**KEGGpath**	Ibid	634:7752	31.57

**KEGGtypepath**	ibid	621:5223	25.11

**KEGG2**	Ibid	609:10518	34.24

**KEGG2type**	Ibid	602:7099	27.44

**KEGG2path**	Ibid	608:8130	31.89

**KEGG2typepath**	Ibid	595:5691	25.65

**iND750_0**	H2O, H+	990:8427	19.19

**iND750_1**	H2O, H+, Pi	976:6995	18.27

**iND750_2**	H2O, H+, Pi, ATP	976:6278	17.85

**iND750_3**	H2O, H+, Pi, ATP, Glu-L	974:5742	17.60

**iND750_4**	H2O, H+, Pi, ATP, Glu-L, ADP	969:5186	17.18

**iND750_0nh**	H2O, H+	634:4761	19.19

**iND750_1nh**	H2O, H+, Pi	619:3963	18.27

**iND750_2nh**	H2O, H+, Pi, ATP	618:3387	17.85

**iND750_3nh**	H2O, H+, Pi, ATP, Glu-L	617:3122	17.60

**iND750_4nh**	H2O, H+, Pi, ATP, Glu-L, ADP	613:2720	17.18

The names of the KEGG and the iND750-derived networks are indicated under the "Network name" column. Note that the last five networks end with an "nh", indicating that hypothetical reactions were not included in these networks. The column labeled "Over-linked metabolites" indicates the metabolites being removed while reconstructing the network: H2O: water; H+: a proton; Pi: inorganic phosphorus; ATP: adenosine triphosphate; Glu-L: L-glutamate and ADP: adenosine diphosphate, NADP+/NADPH: Nicotinamide adenine dinucleotide phosphate, NAD/NADH: Nicotinamide adenine dinucleotide. The number of genes (values in bold) and gene-to-gene relationships (values in italics) of each graph are indicated separated by a colon in the Vertices:Edges column. The column labeled "Overlap (%)" indicates the percentage of known gene-to-gene relationships (network links) found in each reconstructed network reported in the probabilistic functional network of yeast genes [4] that included two metabolic genes. According to the procedure to build these networks, KEGG, KEGGpath, KEGG2 and KEGG2path networks are undirected.

### Pathway insulation and compartamentalization

For decades metabolism has been organized into pathways and recently it has been observed that genes in pathways are often co-regulated [11]. It is also well known that the genes of many pathways are not always expressed. For instance, enzymes involved in aerobic primary metabolism are not present during anaerobic conditions [12]. In order to account for such insulation in metabolic pathways, we built a group of genetic metabolic networks by only linking genes in the same pathway; additionally, we also different pathway through the genes present in these different pathways (*e.g*., KEGGpath network). Alternatively, we build metabolic networks by linking all genes disregarding the pathway (*e.g*., KEGG network) (see Table 1). In summary, all KEGG-derived networks labelled with the "path" keyword include genetic relationships restricted to the annotated pathways, even if they share a metabolite.

Finally, it is well known, that inside eukaryotic cells there are barriers to keep proteins and small molecules sorted in compartments [10]. Such insulation was taken into account in the iND750 derived networks. Specifically, we did not link genes in different cellular compartments. Additionally, two groups of networks were derived from the hand-curated iND750 network. In the first group, all the reactions contained in the original model were considered (*e.g*., iND750_0, see Table 1), while in the second, hypothetic reactions were eliminated (*e.g*., iND750_0nh). For each of these groups, different sets of over-linked metabolites were eliminated from each network (see Table 1).

### Elimination of genetic relationships emerging from highly connected metabolites

Including highly connected metabolites (*e.g*., ATP or water) into reconstructed metabolite networks induce a small average path length of 3 [13]. By eliminating highly connected metabolites, more distinct chemical metabolic networks have been obtained in [14]. All these reconstructed networks were treated as bipartite networks, composed of enzymes and metabolites, and the effect on the prediction of essential genes have not been evaluated. Here, we evaluated the effect that eliminating highly connected metabolites has on the prediction of essential genes using genetic networks. All the KEGG-derived networks eliminated the following over-linked metabolites: water, ATP, ADP, NAD+, NADH, NADP+, NADPH and Oxygen.

### Network properties

Two sets were determined from these genetic networks, the genes in a network (vertices) and the connections between these genes (edges). From these sets, the eccentricity and the radius were determined; eccentricity is defined as the largest shortest path found for a given gene, while the radius is defined as the smallest eccentricity value for all genes. Thus, if there is no path connecting two genes, the radius of a network is equal to infinitum and the network is not connected. This measure was also used to determine the presence of the giant strong component in our models [15].

### Centrality measures

We used 16 different centrality measures (see Table 2 for a mathematical description of each): eccentricity, 1/eccentricity, closeness, average distance, shortest-path betweenness, Katz index (for the network under analysis and the corresponding inverse network), PageRank (for the network under analysis and the corresponding inverse network), radiality, integration, clustering coefficient, 1/clustering coefficient, degree (in and out) and sphere degree (the number of reachable vertices up to a distance of 2) [16-18]. An inverse network is derived from a directed network by switching the nodes in every edge. Note that when a centrality value was equal to zero, the inverse value was set to zero in our study. Eccentricity, average distance, closeness, shortest-path betweenness, Katz index, PageRank, radiality and integration are global centrality measures while the others are local in the sense that the measure only depends on the immediate connections of a gene. Thus, every gene is assigned a centrality value and the list of genes is sorted in a descendent fashion. From this ordered list, we took every possible fraction of the list and determine the correspondence with the known lethal genes coding for metabolic enzymes.

**Table 2:** Centrality measures used in this study

**Centrality**	**Formula**	**Description**
**In degree**	Cu = kin(u)	Number of connections into node u

**Out degree**	Cu = kout(u)	Number of connections out from node u

**Sphere degree**	Cu = kout(u) + ∑_w∩u _kout(w); w is any neighbor of u.	Number of nodes at 1 or 2 connections from node u

**Clustering coefficient**		The fraction of connections between the neighbors of node u

**1/Clustering coefficient**		

**Eccentricity**	Cu = max{dist(u, w): w ∈ V}	The distance between node u and the most distant node in the net.

**1/Eccentricity**	Cu = 1/max{dist(u, w): w ∈ V}	

**Average distance**		Average distance of node u to the rest of nodes in the net

**Closeness**		Inverse of average distance

**Katz**		A node has a larger ckatz value while more paths reach it.

**KatzR**		A node has a larger ckatz value while more paths leave the node.

**PageRank**	c_PR _= d P c_PR _+ ((1 - d) 1)	The centrality of a node depends on its incoming connections and the relative connectivity of these connections

**PageRankR**	c_PR _= d P^T ^c_PR _+ ((1 - d) 1)	The centrality of a node depends on its outcoming connections and the relative connectivity of these connections

**Integration**		The easiness of reaching node u from any other node

**Radiality**		The easiness of reaching any node from node u

**SP-betweenness**		The fraction of shortest paths inside the network, which utilize node u

In the table, kin(u), kout(u) and ktot(u) refer to the incoming, outgoing and total number of edges of node u. diamG refers to the diameter of the graph and dist(u, v) stands for the distance between nodes u and v. In clustering coefficient, |e| stands for the observed paths between the neighbours of a node. In Katz A is the adjacency matrix and α a damping factor. In PageRank d is a damping factor and P the transition matrix. In the formula for shortest path (SP) betweenness σ_G _denotes the number of shortest path from s to t. For a more detailed description of these centralities, please read [17].

Additionally to performing the analysis based on a single centrality, we combined every individual centrality in groups of 2, 3 or 4 centralities and generated a combined centrality score for each gene in the network, CS(v), according to the following formula:



Where MAX_C_i _and MIN_C_i _define the maximum and minimum score obtained for ith-centrality in a given network, respectively, C_i_(v) refers to the centrality of a given gene and *m *in the summation refers to the centrality combinations evaluated; for instance, m = 2 for groups of 2 centralities, m = 3 for groups of 3 centralities and m = 4 for groups of 4 centralities. CS(v) estimates how far from the largest observed centrality measures are the centralities of the genes analysed. Thus, the lower the combined score is, the higher the individual centrality measures are.

### Assessing the reliability of the centrality predictions

The Yeast Genome Consortium [19] provides a list of genes that upon deletion from the yeast genome prevent the yeast growth in YPD media. Those lethal phenotypes arising from deletion of genes involved in metabolism are considered critical for metabolism function, since inactivation of primary metabolism may lead to cell death. This included a total of 246 genes: the iND750 networks in this work have 107 to 115 essential metabolic genes and in the KEGG and KEGG2 networks this number varies from 127 to 131. The iND750 networks and the KEGG networks share 93 essential genes.

The genes of each network were ranked according to each of the 16 centrality values (the 4 non directed networks used 13 measurements). Therefore, we created 276 gene lists to compare with the list of known essential genes when single centrality values were used. When pairs of centrality were used to score each gene, 2,736 comparisons were performed, 13,200 for the groups of 3 centralities, and 48,300 for groups of 4.

The quality of the comparisons was assessed with a Receiver-Operator Characteristic curve (ROC curve), where the sensitivity vs. the false discovery rate (FDR) for each possible cut-off of a ranked gene list is plotted. The area under each ROC curve (AUC) was calculated using an empirical method [20]. The AUC is an estimate of how good a classifier is to differentiate between essential and non-essential genes. This is a variant to the Mann Whitney U statistic. An effective classifier will generate an AUC significantly greater than 0.50 (the expected AUC value for a random classifier), therefore we calculated the confidence intervals (CI) for all AUC scores using the formula CI = ± z * SE(AUC), were z is the z-value for a given significance level (0.1, 0.05, 0.01 in our case) and SE(AUC) is the standard error of the AUC [20]. In addition, we calculated the minimum error (minE) for each ROC curve as follows, minE = (MIN [(1-sensitivity)^2^+(FDR)^2^]^1/2^). This tracks the point of the curve that is closer to a perfect prediction. The prediction accuracy for each ROC curve was calculated as [TP+TN]/[TP+TN+FP+FN] [7]. Sensitivity is defined as TP/(TP+FN), where TP = true positives (genes truly predicted as essential) and FN = false negatives (missed essential genes). FDR is defined as FP/(TN+FP), where TN = true negatives (genes truly predicted as non-critical) and FP = false positives (missed non-essential genes). Note that Specificity = 1 - FDR.

### Estimating the completeness of the genetic networks

We estimated the fraction of identical edges between each reconstructed network in this work with the probabilistic functional network of yeast genes [4]; the percentage of identical links was estimated by dividing this value by the number of edges present in the reconstructed network (See Table 1, column "Overlap").

To estimate the completeness of the networks, the missing relationships of non-predicted essential genes in the KEGG2path network were determined by grouping all essential genes by their Gene Ontology biological process ID. Then, the missing local relationships of unpredicted essential genes in these groups were determined.

Using these two criteria, we estimated the completeness of the reconstructed networks; however, it has to be kept in mind that there is no precise approach on how to estimate the completeness of genetic networks, thus our results may be used as an approximation to this problem.

## Results

The reliable identification of essential genes in a biological network depends on both the network structure and the algorithm used to identify the essentiality of genes. Therefore, by using different methods to analyze alternative representations of the same network, we may identify on one side the algorithm(s) that reproduce(s) the best the feature of essential genes presented in the networks and, on the other hand the network(s) with the largest identifiable number of essential genes. Here we refer to these as Rich on Essential Metabolic Genetic network, or REMG network.

18 different networks were built following the procedures described above (see Methods). These networks can be grouped according to the data source used to build them: KEGG and iND750 (see Table 1). These two data sets constitute the most common sources for metabolism reconstruction in *S. cerevisiae*. Note that when reconstructing a model of metabolism, several assumptions are made regarding the edge set present in this model. In our study we consider some of the most common assumptions, including the reversibility of the relationships, insulation by organelle or function, and the elimination of highly connected metabolites (see Methods). Thus, the genetic networks used in this study are similar (see Table 1) and constitute a sampling of the common reconstructing strategies to build models of metabolism.

All the networks analyzed here were disconnected (*i.e*., these presented at least one gene for which there was not path to connect it with the rest of the genes) and presented a giant component (data not shown), as previously reported for other metabolic networks [15].

### Prediction of essential genes by a single centrality measure

**Figure 2:**
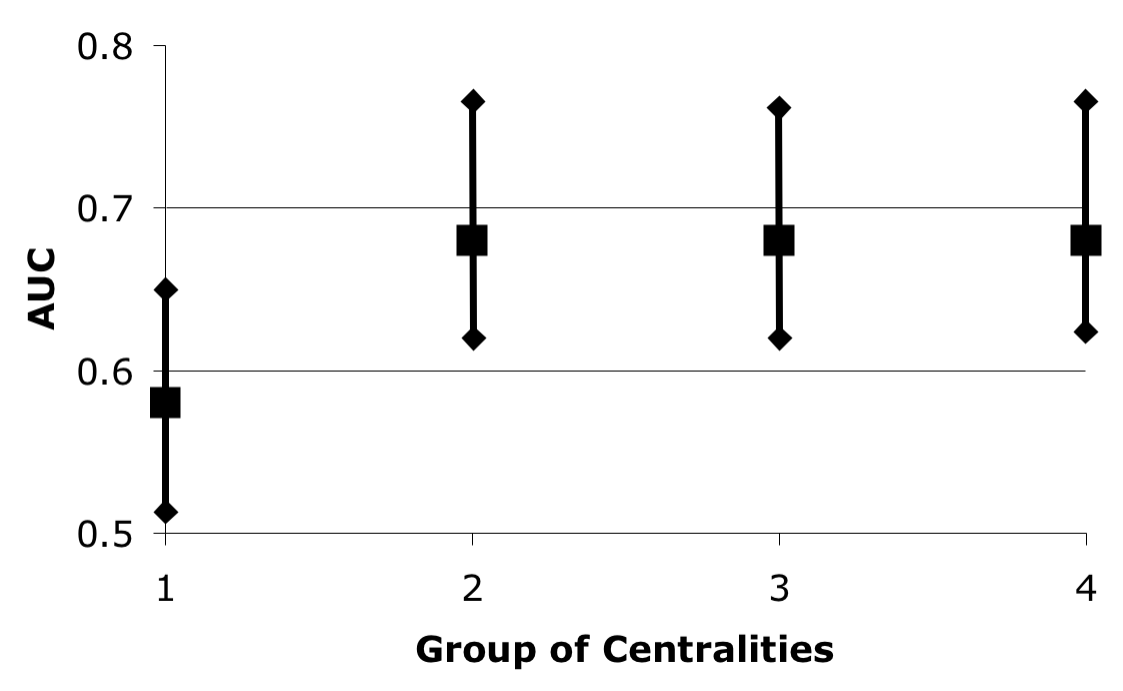
**The efficacy to predict essential metabolic genes from centrality measures**. The maximum Area Under the ROC Curve (max AUC) obtained from the Mann-Whitney test (see Methods) applied to the 18 different metabolic networks used in this study (see Table 1) are shown in the plot as black squares. Each square represent the max AUC obtained from groups of 1, 2, 3 or 4 centrality measures used to differentiate essential from non-essential genes. The vertical lines crossing each square represent the confidence interval at 99%.

Using the Mann Whitney U test normalized by the number of possible pairings (see Methods), we evaluated the efficacy of 16 different centrality measures to differentiate essential from non-essential genes on 18 genetic networks. We selected as the best network-centrality pairs those with i) an AUC score above 0.50 and, ii) an AUC score within a confidence interval above 0.50 with 99% of significance (see Methods). From this analysis, we detected that only the KEGG2path-Closeness pair satisfied these two criteria (see Additional File 1 and Figure 2). However, for the 276 tests performed (14 × 16 + 4 × 13), almost 3 network-centrality pairs were expected by chance to be positive at this level of significance. Thus, our results indicate that single centrality measures may not identify critical genes with statistical significance.

### Prediction of essential genes by combining centrality measures

We noticed that genes predicted by individual centrality measures did not overlap (to access the specific sets of overlapping genes see ); therefore, we hypothesized that combining the predictions from different centralities may improve the reliability achieved by the individual centralities. To test the hypothesis, we combined the scores of 2, 3 and 4 centrality measures in a single Combined Score, CS(v), and assigned this new centrality measure to every gene (see Methods). That is, instead of assigning 16 different centrality measures to every gene in a given network, the number of combined centrality measures was n!/(n-i)!*i! (n: number of individual centrality measures applied to a given network; i: number of combined centralities, i.e., i = 2, 3 or 4). That is, the number of combined centralities were 2,562, 13,244 and 48,300 for the groups of 2, 3 and 4 individual centrality measures, respectively. For each group of combined centralities, the efficiency in differentiating essential from non-essential genes was estimated using a variant of the Mann Whitney U test (see Methods). In this case too, the best network-"combined centrality measures" pairs were selected based on an AUC score > 0.5 where the confidence interval at 99% was > 0.5. Figure 2 shows the maximum AUC scores obtained for groups of 2, 3 and 4 centralities. The numbers of positive hits observed (884, 5,023 and 20,250) are clearly above the expected number of positive hits by chance (26, 132 and 483 for the groups of 2, 3 and 4 combined centrality measurements, respectively), at 99% confidence level (see ,  and ). Particularly, the combined centralities closeness-"1/clustering coefficient", excentricity-"1/clustering coefficient", 1/excentricity-"1/clustering coefficient" and radiality-"1/clustering coefficient" rendered significant predictions in both iND750 and KEGG networks.

### Completeness of REMG networks

The combination of more than 2 centralities did not improve the correct prediction of metabolic essential genes (see Figure 2). In this case, it is possible that some essential genes may have not been detected because REMG networks do not include all the relevant genetic relations for metabolism, among other reasons. Indeed, we observed that those networks did not include all the known genetic relationships for metabolic genes: only 17% to 34% of all known metabolic genetic relationships (see Methods) are included in any given network used in this study (see Table 1).

Furthermore, to identify the relevant missing genetic relationships in a REMG network (KEGG2path network, as detected by combining 1 or 2, 3 or 4 centrality measures; see ,  and  respectively), we aimed at the unpredicted essential metabolic genes. It has been shown that essential genes tend to be part of *Essential Complex Biological Modules *[21]; in that case, the missing local genetic relationships of unpredicted essential genes may be found within these modules. As a proof of concept, essential genes were predicted by both local (Clustering coefficient) and global (Closeness centrality) centrality measures using the minimum error criteria in each case. This identifies 99 essential genes out of 131 total known metabolic essential genes (see ). The missing 32 essential genes may be reduced to 18 by including the essential genes predicted with every local centrality measure (YBR153W, YDR236C, YDR050C, YKL192C, YNR016C, YBR196C, YDL055C, YBL076C, YPR033C, YPL160W, YFL022C, YDR023W, YGR185C, YHR019C, YGR094W, YDR341C, YBR121C, YDR037W). These genes were classified using the April's 2008 Gene Ontology classification [22] and found that 6 (YER003C, YKL152C, YCR012W, YKL182W, YPL231W, YBR256W) out of these 18 unpredicted essential metabolic genes shared the same classification with predicted essential genes (see Additional File 2). According to the reported probabilistic functional gene network of yeast [4], these 6 genes are engaged in 119 different metabolic genetic relations, while only 26 of these are present in the KEGG2path network (data not shown). Thus, the REMG network KEGG2path does have missing genetic relationships.

## Discussion

Our work describes a procedure to identify essential genes from genetic networks (see Figure 1). We claim than in the current scenario where the knowledge on the molecular network structures is not complete yet, it is necessary to test the mathematical operation to reproduce the essentiality of genes beyond a single model network. To achieve this, we report a procedure that simultaneously varies both the network structure and the mathematical operation that reproduces gene essentiality. Considering the infinite number of possible network structures for a given set of genes, a reasonable approach is to use knowledge-based networks reconstructed with different criteria. Hence, we used 2 knowledge-based networks sharing a common set of genes and genetic relationships (*i.e*., KEGG and iND750) and use variations accounting for some of the most common assumptions during metabolic network reconstruction (see Methods and Table 1).

To differentiate essential from non-essential genes on 18 different metabolic genetic networks, a total of 204,233 centrality calculations were performed using 16 different centrality measures. Our results indicate that closeness centrality applied to the KEGG2path network identify the largest fraction of essential metabolic genes (see Additional File 1). As stated, this result is in agreement with previous reports showing that global centrality measures (*e.g*., betweeness, closeness, synthetic lethality) and other chemical-based approaches [23-25] are efficient identifiers of essential genes [26-32]. However, when the overall number of predictions in our study is taken into account, this prediction is not significant: closeness must have been observed at least 4 times as a reliable predictor in these 18 similar networks. Hence, closeness highly depends on the structure of the KEGG2path network to achieve the correct identification of essential genes. We conclude that none of the single centrality measure tested can predict essential genes in a statistically significant way.

The discrepancy of our results with previous reports may be explained also by the different statistical analysis performed: in previous studies the prediction of essential genes is achieved at one particular centrality cut-off value (*e.g*., top 20% of genes with largest centrality values) and for that cut-off value one statistical parameter is reported (*i.e*., sensitivity, specificity, accuracy; see Table 3 for a summary of some of these reports). This assumption may induce errors because the parameters reported in the Table are dependent on the chosen cut-off value: for large cut-off values, the sensitivity tend to present large values and the specificity small ones, while this trend may be reversed for small cut-off values. Our method considered all possible cut-off values and we did not observe any statistical significance in the predictions of gene essentiality with any individual centrality measure.

**Table 3:** Comparison of statistical parameters used to estimate the efficacy to identify essential metabolic genes in yeast

**Model**	**Method**	**Essentials/Non-essentials**	**Sensitivity**	**Specificity**	**Error**	**Accuracy**	**Reference**
**iFF708**	FBA	23/90	0.13	1	0.87	82%	22

**iFF708**	FBA	91/508	0.31	0.95	0.69	85%	6

**iFF708**	FBA	79-146/562-629	0.40-0.53	90-96%	NR	NR	33

**iND750**	FBA	118/NR	0.31	NR	NR	NR	10

**iLL672**	FBA	NR/NR	0.68-0.80	96-98%	NR	NR	33

**iFF708**	MOMA	46/302	0.60	0.92	0.41	88%	34

**iLL672**	MOMA	NR/NR	0.73-0.80%	NR	NR	NR	33

**iND750**	SA	100/NR	0.14	NR	NR	NR	7

Previous studies on the efficiency to identify essential metabolic genes on yeast, report different statistical parameters of confidence (Sensitivity, Specificity, Error and Accuracy columns), and commonly use a single network (Model column) and a single method (Method column) to identify essential genes. The data were obtained from the works reported in the "Reference" column. FBA: Flux-Balance Analysis; MOMA: Method of Minimization of Metabolic Adjustment; SA: Synthetic accessibility; NR: a value not reported in the cited reference.

Because of this, it is valid to ask whether the current network structure of metabolism may be efficiently related to gene essentiality? Recent studies shows that the incomplete knowledge of gene function in metabolism cannot explain incorrect gene essentiality predictions achieved by centrality because other factors may be responsible for the essential role of genes in metabolism [8,21,33,34]. However, we argue that other ways to measure centrality in networks may improve the correct prediction of essential genes. Our argument is based on the observation that different essential genes are predicted by different centrality measures (see ). Thus, combining centrality measures might improve the significance of these predictions.

Indeed, combining 2 centrality measures improved the overall significant predictions (see Figure 2). The combined predictions that specifically achieved the best results included both local and global centralities (see ,  and ). Particularly, the top-ranked combined centralities (AUC > 0.60) successfully identified essential genes in both KEGG and iND750 networks: the observed number of predictions (4 or more) for these 4 combined centralities is above the expected number in a random prediction for the 18 different networks tested (p = 0.01). These combined centralities are composed by the local centrality 1/"clustering coefficient" and the global centralities closeness, radiality and excentricity. Small 1/"clustering coefficient" values identify genes with highly connected neighbouring genes, like those at the edge of networks where the leaf node (last node in the network) only has 1 single neighbour to which is connected; hence, genes with large CS(v) values would tend to be at the end of metabolic pathways where many critical enzymatic reactions are located. On the other hand, closeness, excentricity and radiality estimate how near any gene is from the others; thus, genes with high values of these centralities tend to be hubs for the network communication where many essential proteins are located too. Thus, combining these two centrality measures identify genes that are hubs for metabolism, genes located at the end of metabolic pathways or both.

These results indicate that the best way to represent gene essentiality is by combining the single features estimated by individual centrality measures. This in turn, reflects the complex nature of essential genes [5]. Specifically, genes are essential because they establish different relationships (local and/or global) with other genes of an organism: these genes may have local contacts in pathways essential for cell survival (*e.g*., glycolysis); alternatively, essential genes may have distant relationships with genes in other non-essential pathways that become essential to maintain cellular homeostasis for instance.

It is important to emphasize that although our method improved some limitations of previous works to reliably identify essential genes, it does not correctly predict all the known essential metabolic genes. This is expected, as the essentiality of some genes comes from regulatory dynamics and quantitative aspect of their function. Although such aspects cannot be captured neither by the static networks analyzed in the work nor by the centrality measures tested here, it is possible that some missing essential genes may still be detected by adding missing genetic relations in metabolic networks, if any. To determine if there are missing genetic relationships in our REMG networks, we performed two analyses: i) the identification of known metabolic genetic relationships (see Methods) in our reconstructed networks and ii) the identification of the missing local relationships of non-predicted essential genes (see Methods).

Our results show that many known metabolic genetic relationships are missing in our networks. In that case, some improvements in the prediction of essential genes may be achieved by adding some of these missing genetic relationships; but which are the missing relations to be added?

We show how this can be achieved in a particular REMG network (KEGG2path) by extracting the unpredicted essential genes from the *Essential Complex Biological Modules *[21]. We observed that only 6 out of 18 unpredicted essential metabolic genes share the same classification with truly predicted ones (see Additional File 2) and may have local interactions within the essential complexes they belong to. For instance, the gene YER003C is an unpredicted essential gene that shares the same biological process classification (*i.e*., GDP-mannose biosynthetic process) with essential genes (see Additional File 2); the location of YER003C is at the end of a metabolic network and connects two pathways, thus it may be detected by our approach. Interestingly, the 2 genes that constitute the GDP-mannose biosynthetic process in *S. cerevisiae *were part of the KEGG dataset, but KEGG did not include the GDP-mannose biosynthetic pathway, thus our REMG network did not include this relationship. In summary, our REMG network does have missing relations. Adding some of these may have improved the prediction of essential genes using our method.

As noted above, the essential role for some genes is determined by factors others than those included in network structures, such as the variations on the media composition and/or kinetics. Therefore, our method may complement these other analysis as well as to guide experimentalists to direct experiments to complete our understanding about the structure-function relationship present in metabolism.

## Conclusion

In summary, we describe an effective procedure to identify essential genes from genetic networks. Our method is useful in the current scenario where there is uncertainty about the completeness of the network and consequently, in the correct representation of gene essentiality. We show that essentiality is represented by a combination of centrality measures, revealing the complex nature of the function of essential genes. It is expected that further improvements in essential gene predictions may be achieved by adding missing genetic relationships into metabolic networks.

## Authors' contributions

GDR conceived the original idea, developed some of the codes to generate the networks reported in this work, developed the code to calculate the combined centralities reported in this work and mainly wrote this manuscript. DK implemented many of the codes to calculate the centrality measures reported in this work; he also discussed and wrote parts of this manuscript. GC performed the classification of genes based on the GO database and generated supplementary Figure 1. All authors read and approved the final manuscript.

## Supplementary Material

Additional file 1 **Table S1**. AUC values obtained for local and global centralities on each genetic metabolic network. Click here for file

Additional file 2 **Figure S1**. Unpredicted essential metabolic genes matching GO classification with locally essential genes. Click here for file
