# Supplementary material for: How to identify essential genes from molecular networks?
Source: BMC Syst Biol. 2009 Oct 13;3:102. doi: 10.1186/1752-0509-3-102 (PMC2765966; doi:10.1186/1752-0509-3-102)
Supplement: Additional file 1 — Table S1. AUC values obtained for local and global centralities on each genetic metabolic network. [file 1752-0509-3-102-S1.HTML]

Network name


**A) Local Centralities**

 

|  |  |  |  |  |  |
| --- | --- | --- | --- | --- | --- |
| **Network name** | **Clustering**  **Coefficient** | **1/Clustering Coefficient** | **Sphere Degree** | **In Degree** | **Out Degree** |
| KEGG | **0.58** | 0.42 | 0.47 | 0.50 | ND |
| KEGGtype | 0.50 | 0.41 | 0.48 | **0.55** | 0.49 |
| KEGGpath | **0.58** | 0.45 | **0.51** | 0.50 | ND |
| KEGGtypepath | 0.50 | 0.44 | **0.52** | **0.55** | **0.51** |
| KEGG2 | **0.56** | 0.39 | 0.43 | 0.46 | ND |
| KEGG2type | 0.46 | 0.37 | 0.46 | **0.51** | 0.47 |
| KEGG2path | **0.56** | 0.42 | 0.48 | 0.47 | ND |
| KEGG2typepath | 0.46 | 0.39 | 0.49 | **0.52** | 0.48 |
| iND750\_0 | 0.45 | 0.46 | 0.38 | 0.50 | 0.37 |
| iND750\_1 | 0.47 | 0.46 | 0.39 | **0.51** | 0.39 |
| iND750\_2 | 0.46 | 0.48 | 0.37 | 0.47 | 0.39 |
| iND750\_3 | 0.47 | 0.48 | 0.38 | 0.48 | 0.41 |
| iND750\_4 | 0.46 | 0.46 | 0.37 | 0.47 | 0.38 |
| iND750\_0nh | 0.45 | 0.43 | 0.39 | **0.51** | 0.36 |
| iND750\_1nh | 0.46 | 0.43 | 0.40 | **0.51** | 0.38 |
| iND750\_2nh | 0.45 | 0.45 | 0.37 | 0.47 | 0.38 |
| iND750\_3nh | 0.46 | 0.46 | 0.38 | 0.47 | 0.39 |
| iND750\_4nh | 0.44 | 0.41 | 0.36 | 0.47 | 0.36 |

 

**B) Global Centralities**

 

|  |  |  |  |  |  |  |  |  |  |  |  |
| --- | --- | --- | --- | --- | --- | --- | --- | --- | --- | --- | --- |
| **Network name** | **C1** | **C2** | **C3** | **C4** | **C5** | **C6** | **C7** | **C8** | **C9** | **C10** | **C11** |
| KEGG | 0.47 | **0.52** | 0.48 | **0.53** | 0.46 | **0.53** | ND | 0.48 | ND | 0.46 | 0.42 |
| KEGGtype | 0.48 | 0.44 | 0.47 | 0.44 | 0.50 | **0.56** | **0.51** | 0.45 | **0.54** | 0.45 | 0.45 |
| KEGGpath | **0.58** | 0.44 | **0.57** | 0.42 | 0.50 | **0.53** | ND | 0.48 | ND | 0.50 | 0.40 |
| KEGGtypepath | **0.54** | 0.44 | 0.48 | 0.38 | **0.51** | **0.56** | **0.52** | 0.47 | **0.53** | 0.50 | 0.43 |
| KEGG2 | 0.47 | **0.51** | 0.49 | **0.53** | 0.43 | 0.48 | ND | 0.47 | ND | 0.43 | 0.40 |
| KEGG2type | 0.49 | 0.43 | 0.48 | 0.42 | 0.47 | **0.53** | 0.48 | 0.43 | **0.52** | 0.43 | 0.42 |
| KEGG2path | **0.59** | 0.45 | **0.55** | 0.41 | 0.47 | 0.50 | ND | 0.46 | ND | 0.47 | 0.38 |
| KEGG2typepath | **0.55** | 0.42 | 0.49 | 0.36 | 0.48 | **0.53** | 0.49 | 0.45 | **0.52** | 0.48 | 0.40 |
| iND750\_0 | 0.37 | 0.45 | 0.44 | **0.52** | **0.54** | **0.53** | 0.38 | 0.49 | 0.46 | 0.37 | 0.50 |
| iND750\_1 | 0.38 | 0.43 | 0.44 | 0.49 | **0.57** | **0.54** | 0.39 | 0.50 | 0.46 | 0.38 | **0.51** |
| iND750\_2 | 0.35 | **0.53** | 0.35 | **0.53** | 0.48 | 0.49 | 0.39 | 0.46 | 0.46 | 0.35 | **0.52** |
| iND750\_3 | 0.35 | **0.56** | 0.34 | **0.53** | 0.49 | 0.49 | 0.40 | 0.47 | 0.46 | 0.36 | **0.51** |
| iND750\_4 | 0.35 | **0.56** | 0.32 | **0.53** | 0.49 | 0.48 | 0.37 | 0.47 | 0.46 | 0.35 | 0.50 |
| iND750\_0nh | 0.43 | 0.40 | 0.49 | 0.47 | **0.56** | **0.54** | 0.38 | 0.49 | 0.44 | 0.39 | 0.46 |
| iND750\_1nh | 0.44 | 0.39 | 0.49 | 0.44 | **0.58** | **0.55** | 0.39 | 0.50 | 0.44 | 0.39 | 0.47 |
| iND750\_2nh | 0.39 | 0.48 | 0.40 | 0.49 | 0.48 | 0.49 | 0.37 | 0.47 | 0.43 | 0.35 | 0.48 |
| iND750\_3nh | 0.40 | 0.48 | 0.41 | 0.49 | 0.48 | 0.48 | 0.38 | 0.47 | 0.43 | 0.36 | 0.48 |
| iND750\_4nh | 0.36 | 0.49 | 0.37 | 0.50 | 0.50 | 0.48 | 0.35 | 0.48 | 0.43 | 0.33 | 0.48 |

C1: Closeness; C2: Eccentricity; C3: 1/Eccentricity; C4:
Average Distance; C5: Integration; C6: Katz; C7: Katz for the inversed network;
C8: PageRank; C9: PageRank for the inversed network; C10: Radiality; C11:
Shortest-Path Betweenness.
