# Supplementary material for: How to identify essential genes from molecular networks?
Source: BMC Syst Biol. 2009 Oct 13;3:102. doi: 10.1186/1752-0509-3-102 (PMC2765966; doi:10.1186/1752-0509-3-102)
Supplement: Additional file 2 — Figure S1. Unpredicted essential metabolic genes matching GO classification with locally essential genes. [file 1752-0509-3-102-S2.GZ › SuppFig1.html]

|  |  |  |  |  |  |
| --- | --- | --- | --- | --- | --- |
| |  | | --- | | **PMI40** |  |  |  |  |  | | --- | --- | --- | --- | | |  | | --- | | SGD | | GRID | | KEGG | | |


|  |  |  |  |
| --- | --- | --- | --- |
| |  |  |  | | --- | --- | --- | | | GO:0006057 | mannoprotein biosynthetic process | | --- | --- | | |

|  |  |  |  |
| --- | --- | --- | --- |
| |  |  |  | | --- | --- | --- | | | GO:0006056 | mannoprotein metabolic process | | --- | --- | | |

|  |  |  |  |  |  |  |  |  |
| --- | --- | --- | --- | --- | --- | --- | --- | --- |
| |  |  |  |  |  |  |  |  | | --- | --- | --- | --- | --- | --- | --- | --- | | | GO:0000032 1:12|12:6307 2.26e-02 1:20|12:6307 3.74e-02 | cell wall mannoprotein biosynthetic process | | | | --- | --- | --- | --- | | PMI40 (YER003C) | | Unpredicted Essential Genes |  | | |

|  |  |  |  |
| --- | --- | --- | --- |
| |  |  |  | | --- | --- | --- | | | GO:0006090 | pyruvate metabolic process | | --- | --- | | |

|  |  |  |  |  |  |  |  |  |  |
| --- | --- | --- | --- | --- | --- | --- | --- | --- | --- |
| |  |  |  |  |  |  |  |  |  | | --- | --- | --- | --- | --- | --- | --- | --- | --- | | | GO:0006633 2:16|16:6307 7.09e-04 2:20|16:6307 1.12e-03 | fatty acid biosynthetic process | | | | --- | --- | --- | --- | | FAS1 (YKL182W) | | FAS2 (YPL231W) | | Unpredicted Essential Genes |  | | |

|  |  |  |  |
| --- | --- | --- | --- |
| |  |  |  | | --- | --- | --- | | | GO:0000910 | cytokinesis | | --- | --- | | |

|  |  |  |  |
| --- | --- | --- | --- |
| |  |  |  | | --- | --- | --- | | | GO:0033205 | cytokinesis during cell cycle | | --- | --- | | |

|  |  |  |  |  |  |  |  |  |
| --- | --- | --- | --- | --- | --- | --- | --- | --- |
| |  |  |  |  |  |  |  |  | | --- | --- | --- | --- | --- | --- | --- | --- | | | GO:0000916 1:4|4:6307 2.54e-03 1:20|4:6307 1.26e-02 | contractile ring contraction involved in cytokinesis | | | | --- | --- | --- | --- | | CHS2 (YBR038W) | | Unpredicted Essential Genes |  | | |

|  |  |  |  |
| --- | --- | --- | --- |
| |  |  |  | | --- | --- | --- | | | GO:0006560 | proline metabolic process | | --- | --- | | |

|  |  |  |  |  |  |  |  |  |
| --- | --- | --- | --- | --- | --- | --- | --- | --- |
| |  |  |  |  |  |  |  |  | | --- | --- | --- | --- | --- | --- | --- | --- | | | GO:0006561 1:5|5:6307 3.96e-03 1:20|5:6307 1.58e-02 | proline biosynthetic process | | | | --- | --- | --- | --- | | PRO3 (YER023W) | | Unpredicted Essential Genes |  | | |

|  |  |  |  |
| --- | --- | --- | --- |
| |  |  |  | | --- | --- | --- | | | GO:0009084 | glutamine family amino acid biosynthetic process | | --- | --- | | |

|  |  |  |  |  |  |  |  |  |  |  |
| --- | --- | --- | --- | --- | --- | --- | --- | --- | --- | --- |
| |  |  |  |  |  |  |  |  |  |  | | --- | --- | --- | --- | --- | --- | --- | --- | --- | --- | | | GO:0009082 3:13|13:6307 1.93e-06 3:20|13:6307 7.64e-06 | branched chain family amino acid biosynthetic process | | | | --- | --- | --- | --- | | ILV2 (YMR108W) | | ILV3 (YJR016C) | | ILV5 (YLR355C) | | Unpredicted Essential Genes |  | | |

|  |  |  |  |
| --- | --- | --- | --- |
| |  |  |  | | --- | --- | --- | | | GO:0006771 | riboflavin metabolic process | | --- | --- | | |

|  |  |  |  |
| --- | --- | --- | --- |
| |  |  |  | | --- | --- | --- | | | GO:0042727 | riboflavin and derivative biosynthetic process | | --- | --- | | |

|  |  |  |  |
| --- | --- | --- | --- |
| |  |  |  | | --- | --- | --- | | | GO:0042726 | riboflavin and derivative metabolic process | | --- | --- | | |

|  |  |  |  |
| --- | --- | --- | --- |
| |  |  |  | | --- | --- | --- | | | GO:0006767 | water soluble vitamin metabolic process | | --- | --- | | |

|  |  |  |  |
| --- | --- | --- | --- |
| |  |  |  | | --- | --- | --- | | | GO:0042364 | water soluble vitamin biosynthetic process | | --- | --- | | |

|  |  |  |  |  |  |  |  |  |
| --- | --- | --- | --- | --- | --- | --- | --- | --- |
| |  |  |  |  |  |  |  |  | | --- | --- | --- | --- | --- | --- | --- | --- | | | GO:0009231 1:7|7:6307 7.75e-03 1:20|7:6307 2.20e-02 | riboflavin biosynthetic process | | | | --- | --- | --- | --- | | RIB5 (YBR256C) | | Unpredicted Essential Genes |  | | |

|  |  |  |  |
| --- | --- | --- | --- |
| |  |  |  | | --- | --- | --- | | | GO:0046519 | sphingoid metabolic process | | --- | --- | | |

|  |  |  |  |
| --- | --- | --- | --- |
| |  |  |  | | --- | --- | --- | | | GO:0006665 | sphingolipid metabolic process | | --- | --- | | |

|  |  |  |  |
| --- | --- | --- | --- |
| |  |  |  | | --- | --- | --- | | | GO:0006631 | fatty acid metabolic process | | --- | --- | | |

|  |  |  |  |
| --- | --- | --- | --- |
| |  |  |  | | --- | --- | --- | | | GO:0006643 | membrane lipid metabolic process | | --- | --- | | |

|  |  |  |  |
| --- | --- | --- | --- |
| |  |  |  | | --- | --- | --- | | | GO:0032787 | monocarboxylic acid metabolic process | | --- | --- | | |

|  |  |  |  |
| --- | --- | --- | --- |
| |  |  |  | | --- | --- | --- | | | GO:0046394 | carboxylic acid biosynthetic process | | --- | --- | | |

|  |  |  |  |
| --- | --- | --- | --- |
| |  |  |  | | --- | --- | --- | | | GO:0019752 | carboxylic acid metabolic process | | --- | --- | | |

|  |  |  |  |
| --- | --- | --- | --- |
| |  |  |  | | --- | --- | --- | | | GO:0006399 | tRNA metabolic process | | --- | --- | | |

|  |  |  |  |
| --- | --- | --- | --- |
| |  |  |  | | --- | --- | --- | | | GO:0009100 | glycoprotein metabolic process | | --- | --- | | |

|  |  |  |  |
| --- | --- | --- | --- |
| |  |  |  | | --- | --- | --- | | | GO:0006418 | tRNA aminoacylation for protein translation | | --- | --- | | |

|  |  |  |  |
| --- | --- | --- | --- |
| |  |  |  | | --- | --- | --- | | | GO:0043039 | tRNA aminoacylation | | --- | --- | | |

|  |  |  |  |
| --- | --- | --- | --- |
| |  |  |  | | --- | --- | --- | | | GO:0009064 | glutamine family amino acid metabolic process | | --- | --- | | |

|  |  |  |  |
| --- | --- | --- | --- |
| |  |  |  | | --- | --- | --- | | | GO:0009081 | branched chain family amino acid metabolic process | | --- | --- | | |

|  |  |  |  |
| --- | --- | --- | --- |
| |  |  |  | | --- | --- | --- | | | GO:0008652 | amino acid biosynthetic process | | --- | --- | | |

|  |  |  |  |
| --- | --- | --- | --- |
| |  |  |  | | --- | --- | --- | | | GO:0043038 | amino acid activation | | --- | --- | | |

|  |  |  |  |
| --- | --- | --- | --- |
| |  |  |  | | --- | --- | --- | | | GO:0009309 | amine biosynthetic process | | --- | --- | | |

|  |  |  |  |  |  |  |  |  |
| --- | --- | --- | --- | --- | --- | --- | --- | --- |
| |  |  |  |  |  |  |  |  | | --- | --- | --- | --- | --- | --- | --- | --- | | | GO:0006666 1:2|2:6307 6.34e-04 1:20|2:6307 6.33e-03 | 3 keto sphinganine metabolic process | | | | --- | --- | --- | --- | | TSC10 (YBR265W) | | Unpredicted Essential Genes |  | | |

|  |  |  |  |
| --- | --- | --- | --- |
| |  |  |  | | --- | --- | --- | | | GO:0006520 | amino acid metabolic process | | --- | --- | | |

|  |  |  |  |  |  |  |  |  |
| --- | --- | --- | --- | --- | --- | --- | --- | --- |
| |  |  |  |  |  |  |  |  | | --- | --- | --- | --- | --- | --- | --- | --- | | | GO:0006431 1:3|3:6307 1.43e-03 1:20|3:6307 9.48e-03 | methionyl tRNA aminoacylation | | | | --- | --- | --- | --- | | MES1 (YGR264C) | | Unpredicted Essential Genes |  | | |

|  |  |  |  |
| --- | --- | --- | --- |
| |  |  |  | | --- | --- | --- | | | GO:0043283 | biopolymer metabolic process | | --- | --- | | |

|  |  |  |  |
| --- | --- | --- | --- |
| |  |  |  | | --- | --- | --- | | | GO:0009101 | glycoprotein biosynthetic process | | --- | --- | | |

|  |  |  |  |
| --- | --- | --- | --- |
| |  |  |  | | --- | --- | --- | | | GO:0043284 | biopolymer biosynthetic process | | --- | --- | | |

|  |  |  |  |
| --- | --- | --- | --- |
| |  |  |  | | --- | --- | --- | | | GO:0044267 | cellular protein metabolic process | | --- | --- | | |

|  |  |  |  |
| --- | --- | --- | --- |
| |  |  |  | | --- | --- | --- | | | GO:0033692 | cellular polysaccharide biosynthetic process | | --- | --- | | |

|  |  |  |  |
| --- | --- | --- | --- |
| |  |  |  | | --- | --- | --- | | | GO:0006030 | chitin metabolic process | | --- | --- | | |

|  |  |  |  |
| --- | --- | --- | --- |
| |  |  |  | | --- | --- | --- | | | GO:0006044 | N acetylglucosamine metabolic process | | --- | --- | | |

|  |  |  |  |  |  |  |  |  |  |
| --- | --- | --- | --- | --- | --- | --- | --- | --- | --- |
| |  |  |  |  |  |  |  |  |  | | --- | --- | --- | --- | --- | --- | --- | --- | --- | | | GO:0006048 2:2|2:6307 5.03e-08 2:20|2:6307 9.55e-06 | UDP N acetylglucosamine biosynthetic process | | | | --- | --- | --- | --- | | QRI1 (YDL103C) | | GNA1 (YFL017C) | | Unpredicted Essential Genes |  | | |

|  |  |  |  |  |  |  |  |  |
| --- | --- | --- | --- | --- | --- | --- | --- | --- |
| |  |  |  |  |  |  |  |  | | --- | --- | --- | --- | --- | --- | --- | --- | | | GO:0006031 2:16|16:6307 7.09e-04 2:20|16:6307 1.12e-03 | chitin biosynthetic process | | | | --- | --- | --- | --- | | CHS2 (YBR038W) | | Unpredicted Essential Genes |  | | |

|  |  |  |  |
| --- | --- | --- | --- |
| |  |  |  | | --- | --- | --- | | | GO:0006045 | N acetylglucosamine biosynthetic process | | --- | --- | | |

|  |  |  |  |
| --- | --- | --- | --- |
| |  |  |  | | --- | --- | --- | | | GO:0006042 | glucosamine biosynthetic process | | --- | --- | | |

|  |  |  |  |
| --- | --- | --- | --- |
| |  |  |  | | --- | --- | --- | | | GO:0006041 | glucosamine metabolic process | | --- | --- | | |

|  |  |  |  |
| --- | --- | --- | --- |
| |  |  |  | | --- | --- | --- | | | GO:0006037 | cell wall chitin metabolic process | | --- | --- | | |

|  |  |  |  |
| --- | --- | --- | --- |
| |  |  |  | | --- | --- | --- | | | GO:0031506 | cell wall glycoprotein biosynthetic process | | --- | --- | | |

|  |  |  |  |
| --- | --- | --- | --- |
| |  |  |  | | --- | --- | --- | | | GO:0010383 | cell wall polysaccharide metabolic process | | --- | --- | | |

|  |  |  |  |
| --- | --- | --- | --- |
| |  |  |  | | --- | --- | --- | | | GO:0007047 | cell wall organization and biogenesis | | --- | --- | | |

|  |  |  |  |
| --- | --- | --- | --- |
| |  |  |  | | --- | --- | --- | | | GO:0045229 | external encapsulating structure organization and biogenesis | | --- | --- | | |

|  |  |  |  |  |  |  |  |  |
| --- | --- | --- | --- | --- | --- | --- | --- | --- |
| |  |  |  |  |  |  |  |  | | --- | --- | --- | --- | --- | --- | --- | --- | | | GO:0006038 1:12|12:6307 2.26e-02 1:20|12:6307 3.74e-02 | cell wall chitin biosynthetic process | | | | --- | --- | --- | --- | | PCM1 (YEL058W) | | Unpredicted Essential Genes |  | | |

|  |  |  |  |
| --- | --- | --- | --- |
| |  |  |  | | --- | --- | --- | | | GO:0000271 | polysaccharide biosynthetic process | | --- | --- | | |

|  |  |  |  |
| --- | --- | --- | --- |
| |  |  |  | | --- | --- | --- | | | GO:0046349 | amino sugar biosynthetic process | | --- | --- | | |

|  |  |  |  |
| --- | --- | --- | --- |
| |  |  |  | | --- | --- | --- | | | GO:0005976 | polysaccharide metabolic process | | --- | --- | | |

|  |  |  |  |
| --- | --- | --- | --- |
| |  |  |  | | --- | --- | --- | | | GO:0016051 | carbohydrate biosynthetic process | | --- | --- | | |

|  |  |  |  |
| --- | --- | --- | --- |
| |  |  |  | | --- | --- | --- | | | GO:0006629 | lipid metabolic process | | --- | --- | | |

|  |  |  |  |
| --- | --- | --- | --- |
| |  |  |  | | --- | --- | --- | | | GO:0019538 | protein metabolic process | | --- | --- | | |

|  |  |  |  |
| --- | --- | --- | --- |
| |  |  |  | | --- | --- | --- | | | GO:0005975 | carbohydrate metabolic process | | --- | --- | | |

|  |  |  |  |
| --- | --- | --- | --- |
| |  |  |  | | --- | --- | --- | | | GO:0008610 | lipid biosynthetic process | | --- | --- | | |

|  |  |  |  |  |  |  |  |  |
| --- | --- | --- | --- | --- | --- | --- | --- | --- |
| |  |  |  |  |  |  |  |  | | --- | --- | --- | --- | --- | --- | --- | --- | | | GO:0009059 1:3|3:6307 1.43e-03 1:20|3:6307 9.48e-03 | macromolecule biosynthetic process | | | | --- | --- | --- | --- | | FAS2 (YPL231W) | | Unpredicted Essential Genes |  | | |

|  |  |  |  |
| --- | --- | --- | --- |
| |  |  |  | | --- | --- | --- | | | GO:0006807 | nitrogen compound metabolic process | | --- | --- | | |

|  |  |  |  |
| --- | --- | --- | --- |
| |  |  |  | | --- | --- | --- | | | GO:0043170 | macromolecule metabolic process | | --- | --- | | |

|  |  |  |  |
| --- | --- | --- | --- |
| |  |  |  | | --- | --- | --- | | | GO:0044238 | primary metabolic process | | --- | --- | | |

|  |  |  |  |
| --- | --- | --- | --- |
| |  |  |  | | --- | --- | --- | | | GO:0009058 | biosynthetic process | | --- | --- | | |

|  |  |  |  |
| --- | --- | --- | --- |
| |  |  |  | | --- | --- | --- | | | GO:0006047 | UDP N acetylglucosamine metabolic process | | --- | --- | | |

|  |  |  |  |
| --- | --- | --- | --- |
| |  |  |  | | --- | --- | --- | | | GO:0016070 | RNA metabolic process | | --- | --- | | |

|  |  |  |  |
| --- | --- | --- | --- |
| |  |  |  | | --- | --- | --- | | | GO:0009225 | nucleotide sugar metabolic process | | --- | --- | | |

|  |  |  |  |
| --- | --- | --- | --- |
| |  |  |  | | --- | --- | --- | | | GO:0044264 | cellular polysaccharide metabolic process | | --- | --- | | |

|  |  |  |  |
| --- | --- | --- | --- |
| |  |  |  | | --- | --- | --- | | | GO:0006040 | amino sugar metabolic process | | --- | --- | | |

|  |  |  |  |
| --- | --- | --- | --- |
| |  |  |  | | --- | --- | --- | | | GO:0016053 | organic acid biosynthetic process | | --- | --- | | |

|  |  |  |  |
| --- | --- | --- | --- |
| |  |  |  | | --- | --- | --- | | | GO:0044271 | nitrogen compound biosynthetic process | | --- | --- | | |

|  |  |  |  |
| --- | --- | --- | --- |
| |  |  |  | | --- | --- | --- | | | GO:0009110 | vitamin biosynthetic process | | --- | --- | | |

|  |  |  |  |
| --- | --- | --- | --- |
| |  |  |  | | --- | --- | --- | | | GO:0006412 | translation | | --- | --- | | |

|  |  |  |  |
| --- | --- | --- | --- |
| |  |  |  | | --- | --- | --- | | | GO:0009226 | nucleotide sugar biosynthetic process | | --- | --- | | |

|  |  |  |  |
| --- | --- | --- | --- |
| |  |  |  | | --- | --- | --- | | | GO:0019673 | GDP mannose metabolic process | | --- | --- | | |

|  |  |  |  |
| --- | --- | --- | --- |
| |  |  |  | | --- | --- | --- | | | GO:0006006 | glucose metabolic process | | --- | --- | | |

|  |  |  |  |
| --- | --- | --- | --- |
| |  |  |  | | --- | --- | --- | | | GO:0006013 | mannose metabolic process | | --- | --- | | |

|  |  |  |  |
| --- | --- | --- | --- |
| |  |  |  | | --- | --- | --- | | | GO:0019318 | hexose metabolic process | | --- | --- | | |

|  |  |  |  |  |  |  |  |  |  |
| --- | --- | --- | --- | --- | --- | --- | --- | --- | --- |
| |  |  |  |  |  |  |  |  |  | | --- | --- | --- | --- | --- | --- | --- | --- | --- | | | GO:0006094 2:19|19:6307 1.43e-03 2:20|19:6307 1.58e-03 | gluconeogenesis | | | | --- | --- | --- | --- | | GPM1 (YKL152C) | | PGK1 (YCR012W) | | Unpredicted Essential Genes |  | | |

|  |  |  |  |  |  |  |  |  |
| --- | --- | --- | --- | --- | --- | --- | --- | --- |
| |  |  |  |  |  |  |  |  | | --- | --- | --- | --- | --- | --- | --- | --- | | | GO:0019307 2:3|3:6307 4.53e-07 2:20|3:6307 2.86e-05 | mannose biosynthetic process | | | | --- | --- | --- | --- | | SEC53 (YFL045C) | | Unpredicted Essential Genes |  | | |

|  |  |  |  |
| --- | --- | --- | --- |
| |  |  |  | | --- | --- | --- | | | GO:0019319 | hexose biosynthetic process | | --- | --- | | |

|  |  |  |  |
| --- | --- | --- | --- |
| |  |  |  | | --- | --- | --- | | | GO:0046364 | monosaccharide biosynthetic process | | --- | --- | | |

|  |  |  |  |
| --- | --- | --- | --- |
| |  |  |  | | --- | --- | --- | | | GO:0005996 | monosaccharide metabolic process | | --- | --- | | |

|  |  |  |  |
| --- | --- | --- | --- |
| |  |  |  | | --- | --- | --- | | | GO:0046165 | alcohol biosynthetic process | | --- | --- | | |

|  |  |  |  |
| --- | --- | --- | --- |
| |  |  |  | | --- | --- | --- | | | GO:0006766 | vitamin metabolic process | | --- | --- | | |

|  |  |  |  |
| --- | --- | --- | --- |
| |  |  |  | | --- | --- | --- | | | GO:0042180 | ketone metabolic process | | --- | --- | | |

|  |  |  |  |
| --- | --- | --- | --- |
| |  |  |  | | --- | --- | --- | | | GO:0044255 | cellular lipid metabolic process | | --- | --- | | |

|  |  |  |  |
| --- | --- | --- | --- |
| |  |  |  | | --- | --- | --- | | | GO:0006082 | organic acid metabolic process | | --- | --- | | |

|  |  |  |  |
| --- | --- | --- | --- |
| |  |  |  | | --- | --- | --- | | | GO:0006519 | amino acid and derivative metabolic process | | --- | --- | | |

|  |  |  |  |
| --- | --- | --- | --- |
| |  |  |  | | --- | --- | --- | | | GO:0009308 | amine metabolic process | | --- | --- | | |

|  |  |  |  |
| --- | --- | --- | --- |
| |  |  |  | | --- | --- | --- | | | GO:0044260 | cellular macromolecule metabolic process | | --- | --- | | |

|  |  |  |  |
| --- | --- | --- | --- |
| |  |  |  | | --- | --- | --- | | | GO:0010382 | cell wall metabolic process | | --- | --- | | |

|  |  |  |  |
| --- | --- | --- | --- |
| |  |  |  | | --- | --- | --- | | | GO:0006139 | nucleobase, nucleoside, nucleotide and nucleic acid metabolic process | | --- | --- | | |

|  |  |  |  |
| --- | --- | --- | --- |
| |  |  |  | | --- | --- | --- | | | GO:0044262 | cellular carbohydrate metabolic process | | --- | --- | | |

|  |  |  |  |
| --- | --- | --- | --- |
| |  |  |  | | --- | --- | --- | | | GO:0044249 | cellular biosynthetic process | | --- | --- | | |

|  |  |  |  |
| --- | --- | --- | --- |
| |  |  |  | | --- | --- | --- | | | GO:0006066 | alcohol metabolic process | | --- | --- | | |

|  |  |  |  |
| --- | --- | --- | --- |
| |  |  |  | | --- | --- | --- | | | GO:0032506 | cytokinetic process | | --- | --- | | |

|  |  |  |  |
| --- | --- | --- | --- |
| |  |  |  | | --- | --- | --- | | | GO:0051301 | cell division | | --- | --- | | |

|  |  |  |  |
| --- | --- | --- | --- |
| |  |  |  | | --- | --- | --- | | | GO:0022402 | cell cycle process | | --- | --- | | |

|  |  |  |  |
| --- | --- | --- | --- |
| |  |  |  | | --- | --- | --- | | | GO:0007049 | cell cycle | | --- | --- | | |

|  |  |  |  |
| --- | --- | --- | --- |
| |  |  |  | | --- | --- | --- | | | GO:0016043 | cellular component organization and biogenesis | | --- | --- | | |

|  |  |  |  |
| --- | --- | --- | --- |
| |  |  |  | | --- | --- | --- | | | GO:0044237 | cellular metabolic process | | --- | --- | | |

|  |  |  |  |
| --- | --- | --- | --- |
| |  |  |  | | --- | --- | --- | | | GO:0010467 | gene expression | | --- | --- | | |

|  |  |  |  |
| --- | --- | --- | --- |
| |  |  |  | | --- | --- | --- | | | GO:0008152 | metabolic process | | --- | --- | | |

|  |  |  |  |
| --- | --- | --- | --- |
| |  |  |  | | --- | --- | --- | | | GO:0009987 | cellular process | | --- | --- | | |

|  |  |  |  |
| --- | --- | --- | --- |
| |  |  |  | | --- | --- | --- | | | GO:0008150 | biological\_process | | --- | --- | | |

|  |  |  |  |
| --- | --- | --- | --- |
| |  |  |  | | --- | --- | --- | | | GO:0003673 | Gene\_Ontology | | --- | --- | | |

|  |  |  |  |  |  |  |  |  |
| --- | --- | --- | --- | --- | --- | --- | --- | --- |
| |  |  |  |  |  |  |  |  | | --- | --- | --- | --- | --- | --- | --- | --- | | | GO:0009298 1:2|2:6307 6.34e-04 1:20|2:6307 6.33e-03 | GDP mannose biosynthetic process | | | | --- | --- | --- | --- | | PMI40 (YER003C) | | Unpredicted Essential Genes |  | | |
