# Supplementary material for: How to identify essential genes from molecular networks?
Source: BMC Syst Biol. 2009 Oct 13;3:102. doi: 10.1186/1752-0509-3-102 (PMC2765966; doi:10.1186/1752-0509-3-102)
Supplement: Additional file 2 — Figure S1. Unpredicted essential metabolic genes matching GO classification with locally essential genes. [file 1752-0509-3-102-S2.GZ › BP_global_with_coincidencias.html]

|  |  |
| --- | --- |
| |  | | --- | | The synthesis of RNA on a template of DNA. GOC:jl | |


|  |  |  |  |
| --- | --- | --- | --- |
| |  |  |  | | --- | --- | --- | | | GO:0009150 | purine ribonucleotide metabolic process | | --- | --- | | |

|  |  |  |  |
| --- | --- | --- | --- |
| |  |  |  | | --- | --- | --- | | | GO:0009167 | purine ribonucleoside monophosphate metabolic process | | --- | --- | | |

|  |  |  |  |
| --- | --- | --- | --- |
| |  |  |  | | --- | --- | --- | | | GO:0009161 | ribonucleoside monophosphate metabolic process | | --- | --- | | |

|  |  |  |  |
| --- | --- | --- | --- |
| |  |  |  | | --- | --- | --- | | | GO:0009126 | purine nucleoside monophosphate metabolic process | | --- | --- | | |

|  |  |  |  |  |  |  |  |  |
| --- | --- | --- | --- | --- | --- | --- | --- | --- |
| |  |  |  |  |  |  |  |  | | --- | --- | --- | --- | --- | --- | --- | --- | | | GO:0046037 1:2|2:6307 6.34e-04 1:72|2:6307 2.27e-02 | GMP metabolic process | | | | --- | --- | --- | --- | | GUK1 (YDR454C) | | Genes ausentes |  | | |

|  |  |  |  |
| --- | --- | --- | --- |
| |  |  |  | | --- | --- | --- | | | GO:0000377 | RNA splicing, via transesterification reactions with bulged adenosine as nucleophile | | --- | --- | | |

|  |  |  |  |
| --- | --- | --- | --- |
| |  |  |  | | --- | --- | --- | | | GO:0000375 | RNA splicing, via transesterification reactions | | --- | --- | | |

|  |  |  |  |
| --- | --- | --- | --- |
| |  |  |  | | --- | --- | --- | | | GO:0008380 | RNA splicing | | --- | --- | | |

|  |  |  |  |
| --- | --- | --- | --- |
| |  |  |  | | --- | --- | --- | | | GO:0006374 | nuclear mRNA splicing via U2 type spliceosome | | --- | --- | | |

|  |  |  |  |
| --- | --- | --- | --- |
| |  |  |  | | --- | --- | --- | | | GO:0000398 | nuclear mRNA splicing, via spliceosome | | --- | --- | | |

|  |  |  |  |
| --- | --- | --- | --- |
| |  |  |  | | --- | --- | --- | | | GO:0006397 | mRNA processing | | --- | --- | | |

|  |  |  |  |
| --- | --- | --- | --- |
| |  |  |  | | --- | --- | --- | | | GO:0000388 | spliceosome conformational change to release U4 (or U4atac) and U1 (or U11) | | --- | --- | | |

|  |  |  |  |
| --- | --- | --- | --- |
| |  |  |  | | --- | --- | --- | | | GO:0000393 | spliceosomal conformational changes to generate catalytic conformation | | --- | --- | | |

|  |  |  |  |
| --- | --- | --- | --- |
| |  |  |  | | --- | --- | --- | | | GO:0022618 | protein RNA complex assembly | | --- | --- | | |

|  |  |  |  |  |  |  |  |  |
| --- | --- | --- | --- | --- | --- | --- | --- | --- |
| |  |  |  |  |  |  |  |  | | --- | --- | --- | --- | --- | --- | --- | --- | | | GO:0000396 1:2|2:6307 6.34e-04 1:72|2:6307 2.27e-02 | U2 type spliceosome conformational change to release U4 and U1 | | | | --- | --- | --- | --- | | BRR2 (YER172C) | | Genes ausentes |  | | |

|  |  |  |  |
| --- | --- | --- | --- |
| |  |  |  | | --- | --- | --- | | | GO:0006767 | water soluble vitamin metabolic process | | --- | --- | | |

|  |  |  |  |
| --- | --- | --- | --- |
| |  |  |  | | --- | --- | --- | | | GO:0042364 | water soluble vitamin biosynthetic process | | --- | --- | | |

|  |  |  |  |
| --- | --- | --- | --- |
| |  |  |  | | --- | --- | --- | | | GO:0042559 | pteridine and derivative biosynthetic process | | --- | --- | | |

|  |  |  |  |
| --- | --- | --- | --- |
| |  |  |  | | --- | --- | --- | | | GO:0019933 | cAMP mediated signaling | | --- | --- | | |

|  |  |  |  |
| --- | --- | --- | --- |
| |  |  |  | | --- | --- | --- | | | GO:0007187 | G protein signaling, coupled to cyclic nucleotide second messenger | | --- | --- | | |

|  |  |  |  |
| --- | --- | --- | --- |
| |  |  |  | | --- | --- | --- | | | GO:0007186 | G protein coupled receptor protein signaling pathway | | --- | --- | | |

|  |  |  |  |  |  |  |  |  |
| --- | --- | --- | --- | --- | --- | --- | --- | --- |
| |  |  |  |  |  |  |  |  | | --- | --- | --- | --- | --- | --- | --- | --- | | | GO:0007188 1:2|2:6307 6.34e-04 1:72|2:6307 2.27e-02 | G protein signaling, coupled to cAMP nucleotide second messenger | | | | --- | --- | --- | --- | | CYR1 (YJL005W) | | Genes ausentes |  | | |

|  |  |  |  |
| --- | --- | --- | --- |
| |  |  |  | | --- | --- | --- | | | GO:0000737 | DNA catabolic process, endonucleolytic | | --- | --- | | |

|  |  |  |  |
| --- | --- | --- | --- |
| |  |  |  | | --- | --- | --- | | | GO:0044265 | cellular macromolecule catabolic process | | --- | --- | | |

|  |  |  |  |
| --- | --- | --- | --- |
| |  |  |  | | --- | --- | --- | | | GO:0009259 | ribonucleotide metabolic process | | --- | --- | | |

|  |  |  |  |
| --- | --- | --- | --- |
| |  |  |  | | --- | --- | --- | | | GO:0006163 | purine nucleotide metabolic process | | --- | --- | | |

|  |  |  |  |
| --- | --- | --- | --- |
| |  |  |  | | --- | --- | --- | | | GO:0009123 | nucleoside monophosphate metabolic process | | --- | --- | | |

|  |  |  |  |
| --- | --- | --- | --- |
| |  |  |  | | --- | --- | --- | | | GO:0009132 | nucleoside diphosphate metabolic process | | --- | --- | | |

|  |  |  |  |
| --- | --- | --- | --- |
| |  |  |  | | --- | --- | --- | | | GO:0009262 | deoxyribonucleotide metabolic process | | --- | --- | | |

|  |  |  |  |
| --- | --- | --- | --- |
| |  |  |  | | --- | --- | --- | | | GO:0009117 | nucleotide metabolic process | | --- | --- | | |

|  |  |  |  |
| --- | --- | --- | --- |
| |  |  |  | | --- | --- | --- | | | GO:0006753 | nucleoside phosphate metabolic process | | --- | --- | | |

|  |  |  |  |  |  |  |  |  |
| --- | --- | --- | --- | --- | --- | --- | --- | --- |
| |  |  |  |  |  |  |  |  | | --- | --- | --- | --- | --- | --- | --- | --- | | | GO:0009186 1:2|2:6307 6.34e-04 1:72|2:6307 2.27e-02 | deoxyribonucleoside diphosphate metabolic process | | | | --- | --- | --- | --- | | RNR2 (YJL026W) | | Genes ausentes |  | | |

|  |  |  |  |
| --- | --- | --- | --- |
| |  |  |  | | --- | --- | --- | | | GO:0007015 | actin filament organization | | --- | --- | | |

|  |  |  |  |
| --- | --- | --- | --- |
| |  |  |  | | --- | --- | --- | | | GO:0031532 | actin cytoskeleton reorganization | | --- | --- | | |

|  |  |  |  |
| --- | --- | --- | --- |
| |  |  |  | | --- | --- | --- | | | GO:0030036 | actin cytoskeleton organization and biogenesis | | --- | --- | | |

|  |  |  |  |
| --- | --- | --- | --- |
| |  |  |  | | --- | --- | --- | | | GO:0030029 | actin filament based process | | --- | --- | | |

|  |  |  |  |  |  |  |  |  |
| --- | --- | --- | --- | --- | --- | --- | --- | --- |
| |  |  |  |  |  |  |  |  | | --- | --- | --- | --- | --- | --- | --- | --- | | | GO:0030037 1:4|4:6307 2.54e-03 1:72|4:6307 4.49e-02 | actin filament reorganization during cell cycle | | | | --- | --- | --- | --- | | TOR2 (YKL203C) | | Genes ausentes |  | | |

|  |  |  |  |
| --- | --- | --- | --- |
| |  |  |  | | --- | --- | --- | | | GO:0016116 | carotenoid metabolic process | | --- | --- | | |

|  |  |  |  |
| --- | --- | --- | --- |
| |  |  |  | | --- | --- | --- | | | GO:0016108 | tetraterpenoid metabolic process | | --- | --- | | |

|  |  |  |  |
| --- | --- | --- | --- |
| |  |  |  | | --- | --- | --- | | | GO:0016109 | tetraterpenoid biosynthetic process | | --- | --- | | |

|  |  |  |  |
| --- | --- | --- | --- |
| |  |  |  | | --- | --- | --- | | | GO:0016114 | terpenoid biosynthetic process | | --- | --- | | |

|  |  |  |  |  |  |  |  |  |
| --- | --- | --- | --- | --- | --- | --- | --- | --- |
| |  |  |  |  |  |  |  |  | | --- | --- | --- | --- | --- | --- | --- | --- | | | GO:0006085 1:2|2:6307 6.34e-04 1:72|2:6307 2.27e-02 | acetyl CoA biosynthetic process | | | | --- | --- | --- | --- | | ACS2 (YLR153C) | | Genes ausentes |  | | |

|  |  |  |  |
| --- | --- | --- | --- |
| |  |  |  | | --- | --- | --- | | | GO:0032197 | transposition, RNA mediated | | --- | --- | | |

|  |  |  |  |
| --- | --- | --- | --- |
| |  |  |  | | --- | --- | --- | | | GO:0010528 | regulation of transposition | | --- | --- | | |

|  |  |  |  |
| --- | --- | --- | --- |
| |  |  |  | | --- | --- | --- | | | GO:0050789 | regulation of biological process | | --- | --- | | |

|  |  |  |  |  |  |  |  |  |
| --- | --- | --- | --- | --- | --- | --- | --- | --- |
| |  |  |  |  |  |  |  |  | | --- | --- | --- | --- | --- | --- | --- | --- | | | GO:0010525 1:2|2:6307 6.34e-04 1:72|2:6307 2.27e-02 | regulation of transposition, RNA mediated | | | | --- | --- | --- | --- | | RAD3 (YER171W) | | Genes ausentes |  | | |

|  |  |  |  |
| --- | --- | --- | --- |
| |  |  |  | | --- | --- | --- | | | GO:0018344 | protein geranylgeranylation | | --- | --- | | |

|  |  |  |  |
| --- | --- | --- | --- |
| |  |  |  | | --- | --- | --- | | | GO:0018346 | protein amino acid prenylation | | --- | --- | | |

|  |  |  |  |
| --- | --- | --- | --- |
| |  |  |  | | --- | --- | --- | | | GO:0018342 | protein prenylation | | --- | --- | | |

|  |  |  |  |
| --- | --- | --- | --- |
| |  |  |  | | --- | --- | --- | | | GO:0006497 | protein amino acid lipidation | | --- | --- | | |

|  |  |  |  |  |  |  |  |  |
| --- | --- | --- | --- | --- | --- | --- | --- | --- |
| |  |  |  |  |  |  |  |  | | --- | --- | --- | --- | --- | --- | --- | --- | | | GO:0018348 1:4|4:6307 2.54e-03 1:72|4:6307 4.49e-02 | protein amino acid geranylgeranylation | | | | --- | --- | --- | --- | | CDC43 (YGL155W) | | Genes ausentes |  | | |

|  |  |  |  |  |  |  |  |  |
| --- | --- | --- | --- | --- | --- | --- | --- | --- |
| |  |  |  |  |  |  |  |  | | --- | --- | --- | --- | --- | --- | --- | --- | | | GO:0000165 1:2|2:6307 6.34e-04 1:72|2:6307 2.27e-02 | MAPKKK cascade | | | | --- | --- | --- | --- | | STT4 (YLR305C) | | Genes ausentes |  | | |

|  |  |  |  |  |  |  |  |  |
| --- | --- | --- | --- | --- | --- | --- | --- | --- |
| |  |  |  |  |  |  |  |  | | --- | --- | --- | --- | --- | --- | --- | --- | | | GO:0016051 1:3|3:6307 1.43e-03 1:72|3:6307 3.39e-02 | carbohydrate biosynthetic process | | | | --- | --- | --- | --- | | GFA1 (YKL104C) | | Genes ausentes |  | | |

|  |  |  |  |  |  |  |  |  |
| --- | --- | --- | --- | --- | --- | --- | --- | --- |
| |  |  |  |  |  |  |  |  | | --- | --- | --- | --- | --- | --- | --- | --- | | | GO:0046656 1:4|4:6307 2.54e-03 1:72|4:6307 4.49e-02 | folic acid biosynthetic process | | | | --- | --- | --- | --- | | FOL1 (YNL256W) | | Genes ausentes |  | | |

|  |  |  |  |
| --- | --- | --- | --- |
| |  |  |  | | --- | --- | --- | | | GO:0019935 | cyclic nucleotide mediated signaling | | --- | --- | | |

|  |  |  |  |  |  |  |  |  |  |
| --- | --- | --- | --- | --- | --- | --- | --- | --- | --- |
| |  |  |  |  |  |  |  |  |  | | --- | --- | --- | --- | --- | --- | --- | --- | --- | | | GO:0048015 2:16|16:6307 7.09e-04 2:72|16:6307 1.39e-02 | phosphoinositide mediated signaling | | | | --- | --- | --- | --- | | PIK1 (YNL267W) | | STT4 (YLR305C) | | Genes ausentes |  | | |

|  |  |  |  |
| --- | --- | --- | --- |
| |  |  |  | | --- | --- | --- | | | GO:0007243 | protein kinase cascade | | --- | --- | | |

|  |  |  |  |
| --- | --- | --- | --- |
| |  |  |  | | --- | --- | --- | | | GO:0019932 | second messenger mediated signaling | | --- | --- | | |

|  |  |  |  |
| --- | --- | --- | --- |
| |  |  |  | | --- | --- | --- | | | GO:0007166 | cell surface receptor linked signal transduction | | --- | --- | | |

|  |  |  |  |
| --- | --- | --- | --- |
| |  |  |  | | --- | --- | --- | | | GO:0007242 | intracellular signaling cascade | | --- | --- | | |

|  |  |  |  |
| --- | --- | --- | --- |
| |  |  |  | | --- | --- | --- | | | GO:0007165 | signal transduction | | --- | --- | | |

|  |  |  |  |  |  |  |  |  |  |
| --- | --- | --- | --- | --- | --- | --- | --- | --- | --- |
| |  |  |  |  |  |  |  |  |  | | --- | --- | --- | --- | --- | --- | --- | --- | --- | | | GO:0048017 2:16|16:6307 7.09e-04 2:72|16:6307 1.39e-02 | inositol lipid mediated signaling | | | | --- | --- | --- | --- | | PIK1 (YNL267W) | | STT4 (YLR305C) | | Genes ausentes |  | | |

|  |  |  |  |
| --- | --- | --- | --- |
| |  |  |  | | --- | --- | --- | | | GO:0032196 | transposition | | --- | --- | | |

|  |  |  |  |
| --- | --- | --- | --- |
| |  |  |  | | --- | --- | --- | | | GO:0000018 | regulation of DNA recombination | | --- | --- | | |

|  |  |  |  |  |  |  |  |  |  |  |
| --- | --- | --- | --- | --- | --- | --- | --- | --- | --- | --- |
| |  |  |  |  |  |  |  |  |  |  | | --- | --- | --- | --- | --- | --- | --- | --- | --- | --- | | | GO:0016310 3:10|10:6307 3.43e-07 3:72|10:6307 1.62e-04 | phosphorylation | | | | --- | --- | --- | --- | | ERG8 (YMR220W) | | ERG12 (YMR208W) | | MVD1 (YNR043W) | | Genes ausentes |  | | |

|  |  |  |  |
| --- | --- | --- | --- |
| |  |  |  | | --- | --- | --- | | | GO:0006796 | phosphate metabolic process | | --- | --- | | |

|  |  |  |  |
| --- | --- | --- | --- |
| |  |  |  | | --- | --- | --- | | | GO:0046834 | lipid phosphorylation | | --- | --- | | |

|  |  |  |  |
| --- | --- | --- | --- |
| |  |  |  | | --- | --- | --- | | | GO:0006974 | response to DNA damage stimulus | | --- | --- | | |

|  |  |  |  |
| --- | --- | --- | --- |
| |  |  |  | | --- | --- | --- | | | GO:0006950 | response to stress | | --- | --- | | |

|  |  |  |  |
| --- | --- | --- | --- |
| |  |  |  | | --- | --- | --- | | | GO:0009719 | response to endogenous stimulus | | --- | --- | | |

|  |  |  |  |  |  |  |  |  |  |
| --- | --- | --- | --- | --- | --- | --- | --- | --- | --- |
| |  |  |  |  |  |  |  |  |  | | --- | --- | --- | --- | --- | --- | --- | --- | --- | | | GO:0006284 2:12|12:6307 2.17e-04 2:72|12:6307 7.88e-03 | base excision repair | | | | --- | --- | --- | --- | | POL31 (YJR006W) | | POL3 (YDL102W) | | Genes ausentes |  | | |

|  |  |  |  |  |  |  |  |  |  |
| --- | --- | --- | --- | --- | --- | --- | --- | --- | --- |
| |  |  |  |  |  |  |  |  |  | | --- | --- | --- | --- | --- | --- | --- | --- | --- | | | GO:0006301 2:14|14:6307 4.10e-04 2:72|14:6307 1.07e-02 | postreplication repair | | | | --- | --- | --- | --- | | POL31 (YJR006W) | | POL3 (YDL102W) | | Genes ausentes |  | | |

|  |  |  |  |  |  |  |  |  |
| --- | --- | --- | --- | --- | --- | --- | --- | --- |
| |  |  |  |  |  |  |  |  | | --- | --- | --- | --- | --- | --- | --- | --- | | | GO:0000725 1:4|4:6307 2.54e-03 1:72|4:6307 4.49e-02 | recombinational repair | | | | --- | --- | --- | --- | | DPB11 (YJL090C) | | Genes ausentes |  | | |

|  |  |  |  |  |  |  |  |  |  |  |  |
| --- | --- | --- | --- | --- | --- | --- | --- | --- | --- | --- | --- |
| |  |  |  |  |  |  |  |  |  |  |  | | --- | --- | --- | --- | --- | --- | --- | --- | --- | --- | --- | | | GO:0006298 4:25|25:6307 2.30e-06 4:72|25:6307 1.65e-04 | mismatch repair | | | | --- | --- | --- | --- | | POL31 (YJR006W) | | DPB2 (YPR175W) | | DPB11 (YJL090C) | | POL3 (YDL102W) | | Genes ausentes |  | | |

|  |  |  |  |  |  |  |  |  |
| --- | --- | --- | --- | --- | --- | --- | --- | --- |
| |  |  |  |  |  |  |  |  | | --- | --- | --- | --- | --- | --- | --- | --- | | | GO:0033683 1:2|2:6307 6.34e-04 1:72|2:6307 2.27e-02 | nucleotide excision repair, DNA incision | | | | --- | --- | --- | --- | | RAD3 (YER171W) | | Genes ausentes |  | | |

|  |  |  |  |  |  |  |  |  |  |  |  |  |
| --- | --- | --- | --- | --- | --- | --- | --- | --- | --- | --- | --- | --- |
| |  |  |  |  |  |  |  |  |  |  |  |  | | --- | --- | --- | --- | --- | --- | --- | --- | --- | --- | --- | --- | | | GO:0006289 5:32|32:6307 4.43e-07 5:72|32:6307 2.67e-05 | nucleotide excision repair | | | | --- | --- | --- | --- | | RAD3 (YER171W) | | POL31 (YJR006W) | | DPB2 (YPR175W) | | DPB11 (YJL090C) | | POL3 (YDL102W) | | Genes ausentes |  | | |

|  |  |  |  |
| --- | --- | --- | --- |
| |  |  |  | | --- | --- | --- | | | GO:0006721 | terpenoid metabolic process | | --- | --- | | |

|  |  |  |  |  |  |  |  |  |  |  |
| --- | --- | --- | --- | --- | --- | --- | --- | --- | --- | --- |
| |  |  |  |  |  |  |  |  |  |  | | --- | --- | --- | --- | --- | --- | --- | --- | --- | --- | | | GO:0033014 6:11|11:6307 2.44e-15 6:72|11:6307 7.91e-10 | tetrapyrrole biosynthetic process | | | | --- | --- | --- | --- | | HEM2 (YGL040C) | | HEM3 (YDL205C) | | HEM4 (YOR278W) | | Genes ausentes |  | | |

|  |  |  |  |
| --- | --- | --- | --- |
| |  |  |  | | --- | --- | --- | | | GO:0042168 | heme metabolic process | | --- | --- | | |

|  |  |  |  |  |  |  |  |  |
| --- | --- | --- | --- | --- | --- | --- | --- | --- |
| |  |  |  |  |  |  |  |  | | --- | --- | --- | --- | --- | --- | --- | --- | | | GO:0016117 1:2|2:6307 6.34e-04 1:72|2:6307 2.27e-02 | carotenoid biosynthetic process | | | | --- | --- | --- | --- | | IDI1 (YPL117C) | | Genes ausentes |  | | |

|  |  |  |  |  |  |  |  |  |  |  |  |  |  |
| --- | --- | --- | --- | --- | --- | --- | --- | --- | --- | --- | --- | --- | --- |
| |  |  |  |  |  |  |  |  |  |  |  |  |  | | --- | --- | --- | --- | --- | --- | --- | --- | --- | --- | --- | --- | --- | | | GO:0006779 6:11|11:6307 2.44e-15 6:72|11:6307 7.91e-10 | porphyrin biosynthetic process | | | | --- | --- | --- | --- | | HEM13 (YDR044W) | | HEM2 (YGL040C) | | HEM15 (YOR176W) | | HEM3 (YDL205C) | | HEM12 (YDR047W) | | HEM4 (YOR278W) | | Genes ausentes |  | | |

|  |  |  |  |  |  |  |  |  |  |  |  |  |  |
| --- | --- | --- | --- | --- | --- | --- | --- | --- | --- | --- | --- | --- | --- |
| |  |  |  |  |  |  |  |  |  |  |  |  |  | | --- | --- | --- | --- | --- | --- | --- | --- | --- | --- | --- | --- | --- | | | GO:0006783 6:9|9:6307 8.08e-17 6:72|9:6307 1.46e-10 | heme biosynthetic process | | | | --- | --- | --- | --- | | HEM13 (YDR044W) | | HEM2 (YGL040C) | | HEM15 (YOR176W) | | HEM3 (YDL205C) | | HEM12 (YDR047W) | | HEM4 (YOR278W) | | Genes ausentes |  | | |

|  |  |  |  |  |  |  |  |  |  |  |  |  |  |  |  |  |  |  |
| --- | --- | --- | --- | --- | --- | --- | --- | --- | --- | --- | --- | --- | --- | --- | --- | --- | --- | --- |
| |  |  |  |  |  |  |  |  |  |  |  |  |  |  |  |  |  |  | | --- | --- | --- | --- | --- | --- | --- | --- | --- | --- | --- | --- | --- | --- | --- | --- | --- | --- | | | GO:0006366 11:52|52:6307 1.83e-13 11:72|52:6307 8.11e-12 | transcription from RNA polymerase II promoter | | | | --- | --- | --- | --- | | RPB11 (YOL005C) | | RAD3 (YER171W) | | RPB3 (YIL021W) | | RPB5 (YBR154C) | | RPC10 (YHR143W-A) | | RPB2 (YOR151C) | | RPO21 (YDL140C) | | RPO26 (YPR187W) | | RPB8 (YOR224C) | | RPB7 (YDR404C) | | RPB10 (YOR210W) | | Genes ausentes |  | | |

|  |  |  |  |  |  |  |  |  |  |  |  |  |  |  |  |  |  |
| --- | --- | --- | --- | --- | --- | --- | --- | --- | --- | --- | --- | --- | --- | --- | --- | --- | --- |
| |  |  |  |  |  |  |  |  |  |  |  |  |  |  |  |  |  | | --- | --- | --- | --- | --- | --- | --- | --- | --- | --- | --- | --- | --- | --- | --- | --- | --- | | | GO:0006360 10:24|24:6307 1.37e-19 10:72|24:6307 3.40e-14 | transcription from RNA polymerase I promoter | | | | --- | --- | --- | --- | | RPB5 (YBR154C) | | RPC10 (YHR143W-A) | | RPA135 (YPR010C) | | RPC19 (YNL113W) | | RPC40 (YPR110C) | | RPA43 (YOR340C) | | RPO26 (YPR187W) | | RPB8 (YOR224C) | | RPB10 (YOR210W) | | RPA190 (YOR341W) | | Genes ausentes |  | | |

|  |  |  |  |  |  |  |  |  |  |  |  |  |  |  |  |  |  |  |  |  |  |
| --- | --- | --- | --- | --- | --- | --- | --- | --- | --- | --- | --- | --- | --- | --- | --- | --- | --- | --- | --- | --- | --- |
| |  |  |  |  |  |  |  |  |  |  |  |  |  |  |  |  |  |  |  |  |  | | --- | --- | --- | --- | --- | --- | --- | --- | --- | --- | --- | --- | --- | --- | --- | --- | --- | --- | --- | --- | --- | | | GO:0006383 14:20|20:6307 8.39e-34 14:72|20:6307 6.17e-24 | transcription from RNA polymerase III promoter | | | | --- | --- | --- | --- | | RPC34 (YNR003C) | | RPC31 (YNL151C) | | RPB5 (YBR154C) | | RPC10 (YHR143W-A) | | RPO31 (YOR116C) | | RPC19 (YNL113W) | | RPC40 (YPR110C) | | RPC11 (YDR045C) | | RPC82 (YPR190C) | | RPO26 (YPR187W) | | RPB8 (YOR224C) | | RPB10 (YOR210W) | | RPC53 (YDL150W) | | RET1 (YOR207C) | | Genes ausentes |  | | |

|  |  |  |  |  |  |  |  |  |  |
| --- | --- | --- | --- | --- | --- | --- | --- | --- | --- |
| |  |  |  |  |  |  |  |  |  | | --- | --- | --- | --- | --- | --- | --- | --- | --- | | | GO:0046854 2:5|5:6307 5.02e-06 2:72|5:6307 1.26e-03 | phosphoinositide phosphorylation | | | | --- | --- | --- | --- | | PIK1 (YNL267W) | | STT4 (YLR305C) | | Genes ausentes |  | | |

|  |  |  |  |
| --- | --- | --- | --- |
| |  |  |  | | --- | --- | --- | | | GO:0030384 | phosphoinositide metabolic process | | --- | --- | | |

|  |  |  |  |
| --- | --- | --- | --- |
| |  |  |  | | --- | --- | --- | | | GO:0006650 | glycerophospholipid metabolic process | | --- | --- | | |

|  |  |  |  |
| --- | --- | --- | --- |
| |  |  |  | | --- | --- | --- | | | GO:0006644 | phospholipid metabolic process | | --- | --- | | |

|  |  |  |  |  |  |  |  |  |
| --- | --- | --- | --- | --- | --- | --- | --- | --- |
| |  |  |  |  |  |  |  |  | | --- | --- | --- | --- | --- | --- | --- | --- | | | GO:0046488 1:3|3:6307 1.43e-03 1:72|3:6307 3.39e-02 | phosphatidylinositol metabolic process | | | | --- | --- | --- | --- | | MSS4 (YDR208W) | | Genes ausentes |  | | |

|  |  |  |  |
| --- | --- | --- | --- |
| |  |  |  | | --- | --- | --- | | | GO:0000723 | telomere maintenance | | --- | --- | | |

|  |  |  |  |
| --- | --- | --- | --- |
| |  |  |  | | --- | --- | --- | | | GO:0032200 | telomere organization and biogenesis | | --- | --- | | |

|  |  |  |  |
| --- | --- | --- | --- |
| |  |  |  | | --- | --- | --- | | | GO:0007010 | cytoskeleton organization and biogenesis | | --- | --- | | |

|  |  |  |  |
| --- | --- | --- | --- |
| |  |  |  | | --- | --- | --- | | | GO:0051276 | chromosome organization and biogenesis | | --- | --- | | |

|  |  |  |  |
| --- | --- | --- | --- |
| |  |  |  | | --- | --- | --- | | | GO:0022613 | ribonucleoprotein complex biogenesis and assembly | | --- | --- | | |

|  |  |  |  |
| --- | --- | --- | --- |
| |  |  |  | | --- | --- | --- | | | GO:0022607 | cellular component assembly | | --- | --- | | |

|  |  |  |  |
| --- | --- | --- | --- |
| |  |  |  | | --- | --- | --- | | | GO:0006996 | organelle organization and biogenesis | | --- | --- | | |

|  |  |  |  |  |  |  |  |  |
| --- | --- | --- | --- | --- | --- | --- | --- | --- |
| |  |  |  |  |  |  |  |  | | --- | --- | --- | --- | --- | --- | --- | --- | | | GO:0016233 1:4|4:6307 2.54e-03 1:72|4:6307 4.49e-02 | telomere capping | | | | --- | --- | --- | --- | | POL12 (YBL035C) | | Genes ausentes |  | | |

|  |  |  |  |
| --- | --- | --- | --- |
| |  |  |  | | --- | --- | --- | | | GO:0042158 | lipoprotein biosynthetic process | | --- | --- | | |

|  |  |  |  |
| --- | --- | --- | --- |
| |  |  |  | | --- | --- | --- | | | GO:0006464 | protein modification process | | --- | --- | | |

|  |  |  |  |
| --- | --- | --- | --- |
| |  |  |  | | --- | --- | --- | | | GO:0042157 | lipoprotein metabolic process | | --- | --- | | |

|  |  |  |  |
| --- | --- | --- | --- |
| |  |  |  | | --- | --- | --- | | | GO:0044267 | cellular protein metabolic process | | --- | --- | | |

|  |  |  |  |
| --- | --- | --- | --- |
| |  |  |  | | --- | --- | --- | | | GO:0009110 | vitamin biosynthetic process | | --- | --- | | |

|  |  |  |  |
| --- | --- | --- | --- |
| |  |  |  | | --- | --- | --- | | | GO:0046148 | pigment biosynthetic process | | --- | --- | | |

|  |  |  |  |
| --- | --- | --- | --- |
| |  |  |  | | --- | --- | --- | | | GO:0006412 | translation | | --- | --- | | |

|  |  |  |  |  |  |  |  |  |  |
| --- | --- | --- | --- | --- | --- | --- | --- | --- | --- |
| |  |  |  |  |  |  |  |  |  | | --- | --- | --- | --- | --- | --- | --- | --- | --- | | | GO:0006424 2:3|3:6307 4.53e-07 2:72|3:6307 3.83e-04 | glutamyl tRNA aminoacylation | | | | --- | --- | --- | --- | | GUS1 (YGL245W) | | GLN4 (YOR168W) | | Genes ausentes |  | | |

|  |  |  |  |  |  |  |  |  |
| --- | --- | --- | --- | --- | --- | --- | --- | --- |
| |  |  |  |  |  |  |  |  | | --- | --- | --- | --- | --- | --- | --- | --- | | | GO:0006435 1:2|2:6307 6.34e-04 1:72|2:6307 2.27e-02 | threonyl tRNA aminoacylation | | | | --- | --- | --- | --- | | THS1 (YIL078W) | | Genes ausentes |  | | |

|  |  |  |  |  |  |  |  |  |
| --- | --- | --- | --- | --- | --- | --- | --- | --- |
| |  |  |  |  |  |  |  |  | | --- | --- | --- | --- | --- | --- | --- | --- | | | GO:0006422 1:4|4:6307 2.54e-03 1:72|4:6307 4.49e-02 | aspartyl tRNA aminoacylation | | | | --- | --- | --- | --- | | DPS1 (YLL018C) | | Genes ausentes |  | | |

|  |  |  |  |  |  |  |  |  |  |  |  |  |
| --- | --- | --- | --- | --- | --- | --- | --- | --- | --- | --- | --- | --- |
| |  |  |  |  |  |  |  |  |  |  |  |  | | --- | --- | --- | --- | --- | --- | --- | --- | --- | --- | --- | --- | | | GO:0006418 5:35|35:6307 1.13e-06 5:72|35:6307 4.19e-05 | tRNA aminoacylation for protein translation | | | | --- | --- | --- | --- | | GUS1 (YGL245W) | | WRS1 (YOL097C) | | THS1 (YIL078W) | | DPS1 (YLL018C) | | GLN4 (YOR168W) | | Genes ausentes |  | | |

|  |  |  |  |  |  |  |  |  |
| --- | --- | --- | --- | --- | --- | --- | --- | --- |
| |  |  |  |  |  |  |  |  | | --- | --- | --- | --- | --- | --- | --- | --- | | | GO:0043039 5:37|37:6307 2.00e-06 5:72|37:6307 5.53e-05 | tRNA aminoacylation | | | | --- | --- | --- | --- | | THS1 (YIL078W) | | Genes ausentes |  | | |

|  |  |  |  |
| --- | --- | --- | --- |
| |  |  |  | | --- | --- | --- | | | GO:0043038 | amino acid activation | | --- | --- | | |

|  |  |  |  |  |  |  |  |  |
| --- | --- | --- | --- | --- | --- | --- | --- | --- |
| |  |  |  |  |  |  |  |  | | --- | --- | --- | --- | --- | --- | --- | --- | | | GO:0006436 1:2|2:6307 6.34e-04 1:72|2:6307 2.27e-02 | tryptophanyl tRNA aminoacylation | | | | --- | --- | --- | --- | | WRS1 (YOL097C) | | Genes ausentes |  | | |

|  |  |  |  |
| --- | --- | --- | --- |
| |  |  |  | | --- | --- | --- | | | GO:0006520 | amino acid metabolic process | | --- | --- | | |

|  |  |  |  |
| --- | --- | --- | --- |
| |  |  |  | | --- | --- | --- | | | GO:0032787 | monocarboxylic acid metabolic process | | --- | --- | | |

|  |  |  |  |
| --- | --- | --- | --- |
| |  |  |  | | --- | --- | --- | | | GO:0019752 | carboxylic acid metabolic process | | --- | --- | | |

|  |  |  |  |
| --- | --- | --- | --- |
| |  |  |  | | --- | --- | --- | | | GO:0033013 | tetrapyrrole metabolic process | | --- | --- | | |

|  |  |  |  |
| --- | --- | --- | --- |
| |  |  |  | | --- | --- | --- | | | GO:0006084 | acetyl CoA metabolic process | | --- | --- | | |

|  |  |  |  |
| --- | --- | --- | --- |
| |  |  |  | | --- | --- | --- | | | GO:0009108 | coenzyme biosynthetic process | | --- | --- | | |

|  |  |  |  |
| --- | --- | --- | --- |
| |  |  |  | | --- | --- | --- | | | GO:0006752 | group transfer coenzyme metabolic process | | --- | --- | | |

|  |  |  |  |
| --- | --- | --- | --- |
| |  |  |  | | --- | --- | --- | | | GO:0006778 | porphyrin metabolic process | | --- | --- | | |

|  |  |  |  |
| --- | --- | --- | --- |
| |  |  |  | | --- | --- | --- | | | GO:0051188 | cofactor biosynthetic process | | --- | --- | | |

|  |  |  |  |
| --- | --- | --- | --- |
| |  |  |  | | --- | --- | --- | | | GO:0006732 | coenzyme metabolic process | | --- | --- | | |

|  |  |  |  |
| --- | --- | --- | --- |
| |  |  |  | | --- | --- | --- | | | GO:0042558 | pteridine and derivative metabolic process | | --- | --- | | |

|  |  |  |  |
| --- | --- | --- | --- |
| |  |  |  | | --- | --- | --- | | | GO:0019438 | aromatic compound biosynthetic process | | --- | --- | | |

|  |  |  |  |
| --- | --- | --- | --- |
| |  |  |  | | --- | --- | --- | | | GO:0046655 | folic acid metabolic process | | --- | --- | | |

|  |  |  |  |  |  |  |  |  |  |
| --- | --- | --- | --- | --- | --- | --- | --- | --- | --- |
| |  |  |  |  |  |  |  |  |  | | --- | --- | --- | --- | --- | --- | --- | --- | --- | | | GO:0009396 2:11|11:6307 1.51e-04 2:72|11:6307 6.61e-03 | folic acid and derivative biosynthetic process | | | | --- | --- | --- | --- | | FOL2 (YGR267C) | | FOL1 (YNL256W) | | Genes ausentes |  | | |

|  |  |  |  |  |  |  |  |  |
| --- | --- | --- | --- | --- | --- | --- | --- | --- |
| |  |  |  |  |  |  |  |  | | --- | --- | --- | --- | --- | --- | --- | --- | | | GO:0006760 2:12|12:6307 2.17e-04 2:72|12:6307 7.88e-03 | folic acid and derivative metabolic process | | | | --- | --- | --- | --- | | FOL1 (YNL256W) | | Genes ausentes |  | | |

|  |  |  |  |  |  |  |  |  |  |  |  |  |  |
| --- | --- | --- | --- | --- | --- | --- | --- | --- | --- | --- | --- | --- | --- |
| |  |  |  |  |  |  |  |  |  |  |  |  |  | | --- | --- | --- | --- | --- | --- | --- | --- | --- | --- | --- | --- | --- | | | GO:0008299 6:12|12:6307 9.74e-15 6:72|12:6307 1.57e-09 | isoprenoid biosynthetic process | | | | --- | --- | --- | --- | | IDI1 (YPL117C) | | ERG13 (YML126C) | | ERG9 (YHR190W) | | ERG8 (YMR220W) | | ERG12 (YMR208W) | | MVD1 (YNR043W) | | Genes ausentes |  | | |

|  |  |  |  |  |  |  |  |  |  |  |  |  |  |  |
| --- | --- | --- | --- | --- | --- | --- | --- | --- | --- | --- | --- | --- | --- | --- |
| |  |  |  |  |  |  |  |  |  |  |  |  |  |  | | --- | --- | --- | --- | --- | --- | --- | --- | --- | --- | --- | --- | --- | --- | | | GO:0006694 9:35|35:6307 1.05e-13 9:72|35:6307 1.10e-10 | steroid biosynthetic process | | | | --- | --- | --- | --- | | IDI1 (YPL117C) | | ERG7 (YHR072W) | | ERG13 (YML126C) | | ERG9 (YHR190W) | | ERG8 (YMR220W) | | ERG12 (YMR208W) | | MVD1 (YNR043W) | | Genes ausentes |  | | |

|  |  |  |  |
| --- | --- | --- | --- |
| |  |  |  | | --- | --- | --- | | | GO:0006720 | isoprenoid metabolic process | | --- | --- | | |

|  |  |  |  |
| --- | --- | --- | --- |
| |  |  |  | | --- | --- | --- | | | GO:0006643 | membrane lipid metabolic process | | --- | --- | | |

|  |  |  |  |
| --- | --- | --- | --- |
| |  |  |  | | --- | --- | --- | | | GO:0008202 | steroid metabolic process | | --- | --- | | |

|  |  |  |  |  |  |  |  |  |  |  |  |  |  |  |
| --- | --- | --- | --- | --- | --- | --- | --- | --- | --- | --- | --- | --- | --- | --- |
| |  |  |  |  |  |  |  |  |  |  |  |  |  |  | | --- | --- | --- | --- | --- | --- | --- | --- | --- | --- | --- | --- | --- | --- | | | GO:0008610 9:62|62:6307 6.35e-09 9:72|62:6307 2.47e-08 | lipid biosynthetic process | | | | --- | --- | --- | --- | | IDI1 (YPL117C) | | ERG7 (YHR072W) | | ERG13 (YML126C) | | ERG9 (YHR190W) | | ERG8 (YMR220W) | | ERG12 (YMR208W) | | MVD1 (YNR043W) | | Genes ausentes |  | | |

|  |  |  |  |  |  |  |  |  |  |
| --- | --- | --- | --- | --- | --- | --- | --- | --- | --- |
| |  |  |  |  |  |  |  |  |  | | --- | --- | --- | --- | --- | --- | --- | --- | --- | | | GO:0006695 2:5|5:6307 5.02e-06 2:72|5:6307 1.26e-03 | cholesterol biosynthetic process | | | | --- | --- | --- | --- | | IDI1 (YPL117C) | | ERG9 (YHR190W) | | Genes ausentes |  | | |

|  |  |  |  |
| --- | --- | --- | --- |
| |  |  |  | | --- | --- | --- | | | GO:0008203 | cholesterol metabolic process | | --- | --- | | |

|  |  |  |  |  |  |  |  |  |  |  |  |  |  |
| --- | --- | --- | --- | --- | --- | --- | --- | --- | --- | --- | --- | --- | --- |
| |  |  |  |  |  |  |  |  |  |  |  |  |  | | --- | --- | --- | --- | --- | --- | --- | --- | --- | --- | --- | --- | --- | | | GO:0016126 9:35|35:6307 1.05e-13 9:72|35:6307 1.10e-10 | sterol biosynthetic process | | | | --- | --- | --- | --- | | IDI1 (YPL117C) | | ERG13 (YML126C) | | ERG9 (YHR190W) | | ERG8 (YMR220W) | | ERG12 (YMR208W) | | MVD1 (YNR043W) | | Genes ausentes |  | | |

|  |  |  |  |
| --- | --- | --- | --- |
| |  |  |  | | --- | --- | --- | | | GO:0008204 | ergosterol metabolic process | | --- | --- | | |

|  |  |  |  |
| --- | --- | --- | --- |
| |  |  |  | | --- | --- | --- | | | GO:0016125 | sterol metabolic process | | --- | --- | | |

|  |  |  |  |  |  |  |  |  |  |  |  |  |  |  |  |  |
| --- | --- | --- | --- | --- | --- | --- | --- | --- | --- | --- | --- | --- | --- | --- | --- | --- |
| |  |  |  |  |  |  |  |  |  |  |  |  |  |  |  |  | | --- | --- | --- | --- | --- | --- | --- | --- | --- | --- | --- | --- | --- | --- | --- | --- | | | GO:0006696 9:25|25:6307 9.30e-17 9:72|25:6307 3.48e-12 | ergosterol biosynthetic process | | | | --- | --- | --- | --- | | IDI1 (YPL117C) | | ERG7 (YHR072W) | | ERG13 (YML126C) | | ERG9 (YHR190W) | | ERG8 (YMR220W) | | ERG12 (YMR208W) | | MVD1 (YNR043W) | | ERG1 (YGR175C) | | ERG10 (YPL028W) | | Genes ausentes |  | | |

|  |  |  |  |  |  |  |  |  |
| --- | --- | --- | --- | --- | --- | --- | --- | --- |
| |  |  |  |  |  |  |  |  | | --- | --- | --- | --- | --- | --- | --- | --- | | | GO:0006351 23:81|81:6307 5.87e-26 23:72|81:6307 2.60e-27 | transcription, DNA dependent | | | | --- | --- | --- | --- | | RPO26 (YPR187W) | | Genes ausentes |  | | |

|  |  |  |  |
| --- | --- | --- | --- |
| |  |  |  | | --- | --- | --- | | | GO:0006401 | RNA catabolic process | | --- | --- | | |

|  |  |  |  |
| --- | --- | --- | --- |
| |  |  |  | | --- | --- | --- | | | GO:0006396 | RNA processing | | --- | --- | | |

|  |  |  |  |
| --- | --- | --- | --- |
| |  |  |  | | --- | --- | --- | | | GO:0016071 | mRNA metabolic process | | --- | --- | | |

|  |  |  |  |
| --- | --- | --- | --- |
| |  |  |  | | --- | --- | --- | | | GO:0006399 | tRNA metabolic process | | --- | --- | | |

|  |  |  |  |
| --- | --- | --- | --- |
| |  |  |  | | --- | --- | --- | | | GO:0032774 | RNA biosynthetic process | | --- | --- | | |

|  |  |  |  |
| --- | --- | --- | --- |
| |  |  |  | | --- | --- | --- | | | GO:0005975 | carbohydrate metabolic process | | --- | --- | | |

|  |  |  |  |
| --- | --- | --- | --- |
| |  |  |  | | --- | --- | --- | | | GO:0006629 | lipid metabolic process | | --- | --- | | |

|  |  |  |  |
| --- | --- | --- | --- |
| |  |  |  | | --- | --- | --- | | | GO:0043285 | biopolymer catabolic process | | --- | --- | | |

|  |  |  |  |
| --- | --- | --- | --- |
| |  |  |  | | --- | --- | --- | | | GO:0043412 | biopolymer modification | | --- | --- | | |

|  |  |  |  |
| --- | --- | --- | --- |
| |  |  |  | | --- | --- | --- | | | GO:0065003 | macromolecular complex assembly | | --- | --- | | |

|  |  |  |  |
| --- | --- | --- | --- |
| |  |  |  | | --- | --- | --- | | | GO:0009057 | macromolecule catabolic process | | --- | --- | | |

|  |  |  |  |
| --- | --- | --- | --- |
| |  |  |  | | --- | --- | --- | | | GO:0030258 | lipid modification | | --- | --- | | |

|  |  |  |  |
| --- | --- | --- | --- |
| |  |  |  | | --- | --- | --- | | | GO:0019538 | protein metabolic process | | --- | --- | | |

|  |  |  |  |
| --- | --- | --- | --- |
| |  |  |  | | --- | --- | --- | | | GO:0009059 | macromolecule biosynthetic process | | --- | --- | | |

|  |  |  |  |
| --- | --- | --- | --- |
| |  |  |  | | --- | --- | --- | | | GO:0043283 | biopolymer metabolic process | | --- | --- | | |

|  |  |  |  |
| --- | --- | --- | --- |
| |  |  |  | | --- | --- | --- | | | GO:0009056 | catabolic process | | --- | --- | | |

|  |  |  |  |
| --- | --- | --- | --- |
| |  |  |  | | --- | --- | --- | | | GO:0019222 | regulation of metabolic process | | --- | --- | | |

|  |  |  |  |
| --- | --- | --- | --- |
| |  |  |  | | --- | --- | --- | | | GO:0019748 | secondary metabolic process | | --- | --- | | |

|  |  |  |  |  |  |  |  |  |  |
| --- | --- | --- | --- | --- | --- | --- | --- | --- | --- |
| |  |  |  |  |  |  |  |  |  | | --- | --- | --- | --- | --- | --- | --- | --- | --- | | | GO:0006807 2:19|19:6307 1.43e-03 2:72|19:6307 1.94e-02 | nitrogen compound metabolic process | | | | --- | --- | --- | --- | | GLN1 (YPR035W) | | QNS1 (YHR074W) | | Genes ausentes |  | | |

|  |  |  |  |  |  |  |  |  |
| --- | --- | --- | --- | --- | --- | --- | --- | --- |
| |  |  |  |  |  |  |  |  | | --- | --- | --- | --- | --- | --- | --- | --- | | | GO:0009058 9:99|99:6307 2.16e-05 9:72|99:6307 1.51e-06 | biosynthetic process | | | | --- | --- | --- | --- | | ERG9 (YHR190W) | | Genes ausentes |  | | |

|  |  |  |  |
| --- | --- | --- | --- |
| |  |  |  | | --- | --- | --- | | | GO:0044238 | primary metabolic process | | --- | --- | | |

|  |  |  |  |
| --- | --- | --- | --- |
| |  |  |  | | --- | --- | --- | | | GO:0043170 | macromolecule metabolic process | | --- | --- | | |

|  |  |  |  |  |  |  |  |  |  |  |  |  |  |  |
| --- | --- | --- | --- | --- | --- | --- | --- | --- | --- | --- | --- | --- | --- | --- |
| |  |  |  |  |  |  |  |  |  |  |  |  |  |  | | --- | --- | --- | --- | --- | --- | --- | --- | --- | --- | --- | --- | --- | --- | | | GO:0006273 7:16|16:6307 1.65e-15 7:72|16:6307 1.98e-10 | lagging strand elongation | | | | --- | --- | --- | --- | | POL31 (YJR006W) | | DPB2 (YPR175W) | | DPB11 (YJL090C) | | POL3 (YDL102W) | | PRI2 (YKL045W) | | PRI1 (YIR008C) | | POL12 (YBL035C) | | Genes ausentes |  | | |

|  |  |  |  |
| --- | --- | --- | --- |
| |  |  |  | | --- | --- | --- | | | GO:0006271 | DNA strand elongation during DNA replication | | --- | --- | | |

|  |  |  |  |  |  |  |  |  |
| --- | --- | --- | --- | --- | --- | --- | --- | --- |
| |  |  |  |  |  |  |  |  | | --- | --- | --- | --- | --- | --- | --- | --- | | | GO:0000731 1:3|3:6307 1.43e-03 1:72|3:6307 3.39e-02 | DNA synthesis during DNA repair | | | | --- | --- | --- | --- | | PRI2 (YKL045W) | | Genes ausentes |  | | |

|  |  |  |  |  |  |  |  |  |  |
| --- | --- | --- | --- | --- | --- | --- | --- | --- | --- |
| |  |  |  |  |  |  |  |  |  | | --- | --- | --- | --- | --- | --- | --- | --- | --- | | | GO:0006278 2:9|9:6307 6.48e-05 2:72|9:6307 4.39e-03 | RNA dependent DNA replication | | | | --- | --- | --- | --- | | POL31 (YJR006W) | | POL3 (YDL102W) | | Genes ausentes |  | | |

|  |  |  |  |
| --- | --- | --- | --- |
| |  |  |  | | --- | --- | --- | | | GO:0006261 | DNA dependent DNA replication | | --- | --- | | |

|  |  |  |  |
| --- | --- | --- | --- |
| |  |  |  | | --- | --- | --- | | | GO:0033567 | DNA replication, Okazaki fragment processing | | --- | --- | | |

|  |  |  |  |  |  |  |  |  |  |
| --- | --- | --- | --- | --- | --- | --- | --- | --- | --- |
| |  |  |  |  |  |  |  |  |  | | --- | --- | --- | --- | --- | --- | --- | --- | --- | | | GO:0043137 2:5|5:6307 5.02e-06 2:72|5:6307 1.26e-03 | DNA replication, removal of RNA primer | | | | --- | --- | --- | --- | | POL31 (YJR006W) | | POL3 (YDL102W) | | Genes ausentes |  | | |

|  |  |  |  |  |  |  |  |  |  |  |  |
| --- | --- | --- | --- | --- | --- | --- | --- | --- | --- | --- | --- |
| |  |  |  |  |  |  |  |  |  |  |  | | --- | --- | --- | --- | --- | --- | --- | --- | --- | --- | --- | | | GO:0006270 4:27|27:6307 4.37e-06 4:72|27:6307 2.25e-04 | DNA replication initiation | | | | --- | --- | --- | --- | | DPB11 (YJL090C) | | PRI2 (YKL045W) | | PRI1 (YIR008C) | | POL12 (YBL035C) | | Genes ausentes |  | | |

|  |  |  |  |
| --- | --- | --- | --- |
| |  |  |  | | --- | --- | --- | | | GO:0006308 | DNA catabolic process | | --- | --- | | |

|  |  |  |  |
| --- | --- | --- | --- |
| |  |  |  | | --- | --- | --- | | | GO:0051052 | regulation of DNA metabolic process | | --- | --- | | |

|  |  |  |  |
| --- | --- | --- | --- |
| |  |  |  | | --- | --- | --- | | | GO:0006310 | DNA recombination | | --- | --- | | |

|  |  |  |  |  |  |  |  |  |  |
| --- | --- | --- | --- | --- | --- | --- | --- | --- | --- |
| |  |  |  |  |  |  |  |  |  | | --- | --- | --- | --- | --- | --- | --- | --- | --- | | | GO:0006280 2:9|9:6307 6.48e-05 2:72|9:6307 4.39e-03 | mutagenesis | | | | --- | --- | --- | --- | | POL31 (YJR006W) | | POL3 (YDL102W) | | Genes ausentes |  | | |

|  |  |  |  |  |  |  |  |  |
| --- | --- | --- | --- | --- | --- | --- | --- | --- |
| |  |  |  |  |  |  |  |  | | --- | --- | --- | --- | --- | --- | --- | --- | | | GO:0045005 4:25|25:6307 2.30e-06 4:72|25:6307 1.65e-04 | maintenance of fidelity during DNA dependent DNA replication | | | | --- | --- | --- | --- | | POL3 (YDL102W) | | Genes ausentes |  | | |

|  |  |  |  |
| --- | --- | --- | --- |
| |  |  |  | | --- | --- | --- | | | GO:0006281 | DNA repair | | --- | --- | | |

|  |  |  |  |  |  |  |  |  |  |
| --- | --- | --- | --- | --- | --- | --- | --- | --- | --- |
| |  |  |  |  |  |  |  |  |  | | --- | --- | --- | --- | --- | --- | --- | --- | --- | | | GO:0006269 2:2|2:6307 5.03e-08 2:72|2:6307 1.29e-04 | DNA replication, synthesis of RNA primer | | | | --- | --- | --- | --- | | PRI2 (YKL045W) | | PRI1 (YIR008C) | | Genes ausentes |  | | |

|  |  |  |  |
| --- | --- | --- | --- |
| |  |  |  | | --- | --- | --- | | | GO:0022616 | DNA strand elongation | | --- | --- | | |

|  |  |  |  |  |  |  |  |  |  |  |  |  |  |  |  |
| --- | --- | --- | --- | --- | --- | --- | --- | --- | --- | --- | --- | --- | --- | --- | --- |
| |  |  |  |  |  |  |  |  |  |  |  |  |  |  |  | | --- | --- | --- | --- | --- | --- | --- | --- | --- | --- | --- | --- | --- | --- | --- | | | GO:0006260 8:87|87:6307 2.28e-05 8:72|87:6307 5.52e-06 | DNA replication | | | | --- | --- | --- | --- | | POL31 (YJR006W) | | DPB2 (YPR175W) | | DPB11 (YJL090C) | | POL3 (YDL102W) | | PRI2 (YKL045W) | | PRI1 (YIR008C) | | RNR2 (YJL026W) | | POL12 (YBL035C) | | Genes ausentes |  | | |

|  |  |  |  |
| --- | --- | --- | --- |
| |  |  |  | | --- | --- | --- | | | GO:0055086 | nucleobase, nucleoside and nucleotide metabolic process | | --- | --- | | |

|  |  |  |  |
| --- | --- | --- | --- |
| |  |  |  | | --- | --- | --- | | | GO:0019219 | regulation of nucleobase, nucleoside, nucleotide and nucleic acid metabolic process | | --- | --- | | |

|  |  |  |  |
| --- | --- | --- | --- |
| |  |  |  | | --- | --- | --- | | | GO:0006350 | transcription | | --- | --- | | |

|  |  |  |  |
| --- | --- | --- | --- |
| |  |  |  | | --- | --- | --- | | | GO:0016070 | RNA metabolic process | | --- | --- | | |

|  |  |  |  |
| --- | --- | --- | --- |
| |  |  |  | | --- | --- | --- | | | GO:0006259 | DNA metabolic process | | --- | --- | | |

|  |  |  |  |
| --- | --- | --- | --- |
| |  |  |  | | --- | --- | --- | | | GO:0006766 | vitamin metabolic process | | --- | --- | | |

|  |  |  |  |
| --- | --- | --- | --- |
| |  |  |  | | --- | --- | --- | | | GO:0044248 | cellular catabolic process | | --- | --- | | |

|  |  |  |  |
| --- | --- | --- | --- |
| |  |  |  | | --- | --- | --- | | | GO:0031323 | regulation of cellular metabolic process | | --- | --- | | |

|  |  |  |  |
| --- | --- | --- | --- |
| |  |  |  | | --- | --- | --- | | | GO:0006793 | phosphorus metabolic process | | --- | --- | | |

|  |  |  |  |
| --- | --- | --- | --- |
| |  |  |  | | --- | --- | --- | | | GO:0042440 | pigment metabolic process | | --- | --- | | |

|  |  |  |  |
| --- | --- | --- | --- |
| |  |  |  | | --- | --- | --- | | | GO:0044260 | cellular macromolecule metabolic process | | --- | --- | | |

|  |  |  |  |
| --- | --- | --- | --- |
| |  |  |  | | --- | --- | --- | | | GO:0044249 | cellular biosynthetic process | | --- | --- | | |

|  |  |  |  |
| --- | --- | --- | --- |
| |  |  |  | | --- | --- | --- | | | GO:0006519 | amino acid and derivative metabolic process | | --- | --- | | |

|  |  |  |  |
| --- | --- | --- | --- |
| |  |  |  | | --- | --- | --- | | | GO:0009308 | amine metabolic process | | --- | --- | | |

|  |  |  |  |
| --- | --- | --- | --- |
| |  |  |  | | --- | --- | --- | | | GO:0006082 | organic acid metabolic process | | --- | --- | | |

|  |  |  |  |
| --- | --- | --- | --- |
| |  |  |  | | --- | --- | --- | | | GO:0046483 | heterocycle metabolic process | | --- | --- | | |

|  |  |  |  |
| --- | --- | --- | --- |
| |  |  |  | | --- | --- | --- | | | GO:0051186 | cofactor metabolic process | | --- | --- | | |

|  |  |  |  |
| --- | --- | --- | --- |
| |  |  |  | | --- | --- | --- | | | GO:0006725 | aromatic compound metabolic process | | --- | --- | | |

|  |  |  |  |
| --- | --- | --- | --- |
| |  |  |  | | --- | --- | --- | | | GO:0044255 | cellular lipid metabolic process | | --- | --- | | |

|  |  |  |  |
| --- | --- | --- | --- |
| |  |  |  | | --- | --- | --- | | | GO:0006066 | alcohol metabolic process | | --- | --- | | |

|  |  |  |  |  |  |  |  |  |  |
| --- | --- | --- | --- | --- | --- | --- | --- | --- | --- |
| |  |  |  |  |  |  |  |  |  | | --- | --- | --- | --- | --- | --- | --- | --- | --- | | | GO:0006139 2:21|21:6307 2.13e-03 2:72|21:6307 2.35e-02 | nucleobase, nucleoside, nucleotide and nucleic acid metabolic process | | | | --- | --- | --- | --- | | RAD3 (YER171W) | | POL3 (YDL102W) | | Genes ausentes |  | | |

|  |  |  |  |
| --- | --- | --- | --- |
| |  |  |  | | --- | --- | --- | | | GO:0022402 | cell cycle process | | --- | --- | | |

|  |  |  |  |
| --- | --- | --- | --- |
| |  |  |  | | --- | --- | --- | | | GO:0007049 | cell cycle | | --- | --- | | |

|  |  |  |  |
| --- | --- | --- | --- |
| |  |  |  | | --- | --- | --- | | | GO:0050794 | regulation of cellular process | | --- | --- | | |

|  |  |  |  |
| --- | --- | --- | --- |
| |  |  |  | | --- | --- | --- | | | GO:0007154 | cell communication | | --- | --- | | |

|  |  |  |  |
| --- | --- | --- | --- |
| |  |  |  | | --- | --- | --- | | | GO:0016043 | cellular component organization and biogenesis | | --- | --- | | |

|  |  |  |  |  |  |  |  |  |
| --- | --- | --- | --- | --- | --- | --- | --- | --- |
| |  |  |  |  |  |  |  |  | | --- | --- | --- | --- | --- | --- | --- | --- | | | GO:0044237 3:36|36:6307 1.07e-03 3:72|36:6307 7.78e-03 | cellular metabolic process | | | | --- | --- | --- | --- | | FOL1 (YNL256W) | | Genes ausentes |  | | |

|  |  |  |  |
| --- | --- | --- | --- |
| |  |  |  | | --- | --- | --- | | | GO:0065007 | biological regulation | | --- | --- | | |

|  |  |  |  |
| --- | --- | --- | --- |
| |  |  |  | | --- | --- | --- | | | GO:0050896 | response to stimulus | | --- | --- | | |

|  |  |  |  |
| --- | --- | --- | --- |
| |  |  |  | | --- | --- | --- | | | GO:0010467 | gene expression | | --- | --- | | |

|  |  |  |  |
| --- | --- | --- | --- |
| |  |  |  | | --- | --- | --- | | | GO:0008152 | metabolic process | | --- | --- | | |

|  |  |  |  |
| --- | --- | --- | --- |
| |  |  |  | | --- | --- | --- | | | GO:0009987 | cellular process | | --- | --- | | |

|  |  |  |  |
| --- | --- | --- | --- |
| |  |  |  | | --- | --- | --- | | | GO:0008150 | biological\_process | | --- | --- | | |

|  |  |  |  |
| --- | --- | --- | --- |
| |  |  |  | | --- | --- | --- | | | GO:0003673 | Gene\_Ontology | | --- | --- | | |

|  |  |  |  |  |  |  |  |  |  |  |  |
| --- | --- | --- | --- | --- | --- | --- | --- | --- | --- | --- | --- |
| |  |  |  |  |  |  |  |  |  |  |  | | --- | --- | --- | --- | --- | --- | --- | --- | --- | --- | --- | | | GO:0006272 4:14|14:6307 1.50e-08 4:72|14:6307 1.43e-05 | leading strand elongation | | | | --- | --- | --- | --- | | POL31 (YJR006W) | | DPB2 (YPR175W) | | DPB11 (YJL090C) | | POL3 (YDL102W) | | Genes ausentes |  | | |
