# Supplementary material for: How to identify essential genes from molecular networks?
Source: BMC Syst Biol. 2009 Oct 13;3:102. doi: 10.1186/1752-0509-3-102 (PMC2765966; doi:10.1186/1752-0509-3-102)
Supplement: Additional file 2 — Figure S1. Unpredicted essential metabolic genes matching GO classification with locally essential genes. [file 1752-0509-3-102-S2.GZ › BP_local_with_coincidencias.html]

|  |  |  |  |
| --- | --- | --- | --- |
| |  |  |  | | --- | --- | --- | | | GO:0031123 | RNA 3 end processing | | --- | --- | | |

|  |  |  |  |
| --- | --- | --- | --- |
| |  |  |  | | --- | --- | --- | | | GO:0006397 | mRNA processing | | --- | --- | | |

|  |  |  |  |  |  |  |  |  |
| --- | --- | --- | --- | --- | --- | --- | --- | --- |
| |  |  |  |  |  |  |  |  | | --- | --- | --- | --- | --- | --- | --- | --- | | | GO:0031124 1:7|7:6307 7.75e-03 1:39|7:6307 4.25e-02 | mRNA 3 end processing | | | | --- | --- | --- | --- | | CTK1 (YKL139W) | | Genes ausentes |  | | |

|  |  |  |  |
| --- | --- | --- | --- |
| |  |  |  | | --- | --- | --- | | | GO:0005991 | trehalose metabolic process | | --- | --- | | |

|  |  |  |  |  |  |  |  |  |
| --- | --- | --- | --- | --- | --- | --- | --- | --- |
| |  |  |  |  |  |  |  |  | | --- | --- | --- | --- | --- | --- | --- | --- | | | GO:0005992 1:7|7:6307 7.75e-03 1:39|7:6307 4.25e-02 | trehalose biosynthetic process | | | | --- | --- | --- | --- | | UGP1 (YKL035W) | | Genes ausentes |  | | |

|  |  |  |  |
| --- | --- | --- | --- |
| |  |  |  | | --- | --- | --- | | | GO:0006206 | pyrimidine base metabolic process | | --- | --- | | |

|  |  |  |  |  |  |  |  |  |
| --- | --- | --- | --- | --- | --- | --- | --- | --- |
| |  |  |  |  |  |  |  |  | | --- | --- | --- | --- | --- | --- | --- | --- | | | GO:0008655 1:5|5:6307 3.96e-03 1:39|5:6307 3.05e-02 | pyrimidine salvage | | | | --- | --- | --- | --- | | FUR1 (YHR128W) | | Genes ausentes |  | | |

|  |  |  |  |
| --- | --- | --- | --- |
| |  |  |  | | --- | --- | --- | | | GO:0046488 | phosphatidylinositol metabolic process | | --- | --- | | |

|  |  |  |  |
| --- | --- | --- | --- |
| |  |  |  | | --- | --- | --- | | | GO:0030384 | phosphoinositide metabolic process | | --- | --- | | |

|  |  |  |  |
| --- | --- | --- | --- |
| |  |  |  | | --- | --- | --- | | | GO:0006650 | glycerophospholipid metabolic process | | --- | --- | | |

|  |  |  |  |
| --- | --- | --- | --- |
| |  |  |  | | --- | --- | --- | | | GO:0046489 | phosphoinositide biosynthetic process | | --- | --- | | |

|  |  |  |  |
| --- | --- | --- | --- |
| |  |  |  | | --- | --- | --- | | | GO:0046474 | glycerophospholipid biosynthetic process | | --- | --- | | |

|  |  |  |  |  |  |  |  |  |
| --- | --- | --- | --- | --- | --- | --- | --- | --- |
| |  |  |  |  |  |  |  |  | | --- | --- | --- | --- | --- | --- | --- | --- | | | GO:0006661 1:2|2:6307 6.34e-04 1:39|2:6307 1.23e-02 | phosphatidylinositol biosynthetic process | | | | --- | --- | --- | --- | | PIS1 (YPR113W) | | Genes ausentes |  | | |

|  |  |  |  |  |  |  |  |  |
| --- | --- | --- | --- | --- | --- | --- | --- | --- |
| |  |  |  |  |  |  |  |  | | --- | --- | --- | --- | --- | --- | --- | --- | | | GO:0006772 1:2|2:6307 6.34e-04 1:39|2:6307 1.23e-02 | thiamin metabolic process | | | | --- | --- | --- | --- | | THI80 (YOR143C) | | Genes ausentes |  | | |

|  |  |  |  |
| --- | --- | --- | --- |
| |  |  |  | | --- | --- | --- | | | GO:0006077 | 1,6 beta glucan metabolic process | | --- | --- | | |

|  |  |  |  |
| --- | --- | --- | --- |
| |  |  |  | | --- | --- | --- | | | GO:0051273 | beta glucan metabolic process | | --- | --- | | |

|  |  |  |  |
| --- | --- | --- | --- |
| |  |  |  | | --- | --- | --- | | | GO:0051274 | beta glucan biosynthetic process | | --- | --- | | |

|  |  |  |  |
| --- | --- | --- | --- |
| |  |  |  | | --- | --- | --- | | | GO:0009250 | glucan biosynthetic process | | --- | --- | | |

|  |  |  |  |
| --- | --- | --- | --- |
| |  |  |  | | --- | --- | --- | | | GO:0006073 | glucan metabolic process | | --- | --- | | |

|  |  |  |  |
| --- | --- | --- | --- |
| |  |  |  | | --- | --- | --- | | | GO:0033692 | cellular polysaccharide biosynthetic process | | --- | --- | | |

|  |  |  |  |
| --- | --- | --- | --- |
| |  |  |  | | --- | --- | --- | | | GO:0051278 | fungal type cell wall polysaccharide biosynthetic process | | --- | --- | | |

|  |  |  |  |
| --- | --- | --- | --- |
| |  |  |  | | --- | --- | --- | | | GO:0009272 | fungal type cell wall biogenesis | | --- | --- | | |

|  |  |  |  |
| --- | --- | --- | --- |
| |  |  |  | | --- | --- | --- | | | GO:0031505 | fungal type cell wall organization and biogenesis | | --- | --- | | |

|  |  |  |  |
| --- | --- | --- | --- |
| |  |  |  | | --- | --- | --- | | | GO:0042546 | cell wall biogenesis | | --- | --- | | |

|  |  |  |  |
| --- | --- | --- | --- |
| |  |  |  | | --- | --- | --- | | | GO:0007047 | cell wall organization and biogenesis | | --- | --- | | |

|  |  |  |  |  |  |  |  |  |
| --- | --- | --- | --- | --- | --- | --- | --- | --- |
| |  |  |  |  |  |  |  |  | | --- | --- | --- | --- | --- | --- | --- | --- | | | GO:0006078 1:8|8:6307 1.01e-02 1:39|8:6307 4.84e-02 | 1,6 beta glucan biosynthetic process | | | | --- | --- | --- | --- | | UGP1 (YKL035W) | | Genes ausentes |  | | |

|  |  |  |  |  |  |  |  |  |
| --- | --- | --- | --- | --- | --- | --- | --- | --- |
| |  |  |  |  |  |  |  |  | | --- | --- | --- | --- | --- | --- | --- | --- | | | GO:0009105 1:2|2:6307 6.34e-04 1:39|2:6307 1.23e-02 | lipoic acid biosynthetic process | | | | --- | --- | --- | --- | | ACP1 (YKL192C) | | Genes ausentes |  | | |

|  |  |  |  |
| --- | --- | --- | --- |
| |  |  |  | | --- | --- | --- | | | GO:0006544 | glycine metabolic process | | --- | --- | | |

|  |  |  |  |
| --- | --- | --- | --- |
| |  |  |  | | --- | --- | --- | | | GO:0009070 | serine family amino acid biosynthetic process | | --- | --- | | |

|  |  |  |  |  |  |  |  |  |
| --- | --- | --- | --- | --- | --- | --- | --- | --- |
| |  |  |  |  |  |  |  |  | | --- | --- | --- | --- | --- | --- | --- | --- | | | GO:0006545 1:2|2:6307 6.34e-04 1:39|2:6307 1.23e-02 | glycine biosynthetic process | | | | --- | --- | --- | --- | | DFR1 (YOR236W) | | Genes ausentes |  | | |

|  |  |  |  |
| --- | --- | --- | --- |
| |  |  |  | | --- | --- | --- | | | GO:0006801 | superoxide metabolic process | | --- | --- | | |

|  |  |  |  |  |  |  |  |  |
| --- | --- | --- | --- | --- | --- | --- | --- | --- |
| |  |  |  |  |  |  |  |  | | --- | --- | --- | --- | --- | --- | --- | --- | | | GO:0019430 1:2|2:6307 6.34e-04 1:39|2:6307 1.23e-02 | removal of superoxide radicals | | | | --- | --- | --- | --- | | TRR1 (YDR353W) | | Genes ausentes |  | | |

|  |  |  |  |
| --- | --- | --- | --- |
| |  |  |  | | --- | --- | --- | | | GO:0006771 | riboflavin metabolic process | | --- | --- | | |

|  |  |  |  |
| --- | --- | --- | --- |
| |  |  |  | | --- | --- | --- | | | GO:0042727 | riboflavin and derivative biosynthetic process | | --- | --- | | |

|  |  |  |  |  |  |  |  |  |  |
| --- | --- | --- | --- | --- | --- | --- | --- | --- | --- |
| |  |  |  |  |  |  |  |  |  | | --- | --- | --- | --- | --- | --- | --- | --- | --- | | | GO:0009231 2:7|7:6307 2.21e-05 2:39|7:6307 7.67e-04 | riboflavin biosynthetic process | | | | --- | --- | --- | --- | | RIB7 (YBR153W) | | FMN1 (YDR236C) | | Genes ausentes |  | | |

|  |  |  |  |
| --- | --- | --- | --- |
| |  |  |  | | --- | --- | --- | | | GO:0006997 | nuclear organization and biogenesis | | --- | --- | | |

|  |  |  |  |  |  |  |  |  |
| --- | --- | --- | --- | --- | --- | --- | --- | --- |
| |  |  |  |  |  |  |  |  | | --- | --- | --- | --- | --- | --- | --- | --- | | | GO:0006998 1:7|7:6307 7.75e-03 1:39|7:6307 4.25e-02 | nuclear membrane organization and biogenesis | | | | --- | --- | --- | --- | | ACC1 (YNR016C) | | Genes ausentes |  | | |

|  |  |  |  |
| --- | --- | --- | --- |
| |  |  |  | | --- | --- | --- | | | GO:0032784 | regulation of RNA elongation | | --- | --- | | |

|  |  |  |  |
| --- | --- | --- | --- |
| |  |  |  | | --- | --- | --- | | | GO:0051254 | positive regulation of RNA metabolic process | | --- | --- | | |

|  |  |  |  |
| --- | --- | --- | --- |
| |  |  |  | | --- | --- | --- | | | GO:0045935 | positive regulation of nucleobase, nucleoside, nucleotide and nucleic acid metabolic process | | --- | --- | | |

|  |  |  |  |
| --- | --- | --- | --- |
| |  |  |  | | --- | --- | --- | | | GO:0031325 | positive regulation of cellular metabolic process | | --- | --- | | |

|  |  |  |  |
| --- | --- | --- | --- |
| |  |  |  | | --- | --- | --- | | | GO:0009893 | positive regulation of metabolic process | | --- | --- | | |

|  |  |  |  |
| --- | --- | --- | --- |
| |  |  |  | | --- | --- | --- | | | GO:0048522 | positive regulation of cellular process | | --- | --- | | |

|  |  |  |  |
| --- | --- | --- | --- |
| |  |  |  | | --- | --- | --- | | | GO:0048518 | positive regulation of biological process | | --- | --- | | |

|  |  |  |  |
| --- | --- | --- | --- |
| |  |  |  | | --- | --- | --- | | | GO:0050789 | regulation of biological process | | --- | --- | | |

|  |  |  |  |  |  |  |  |  |
| --- | --- | --- | --- | --- | --- | --- | --- | --- |
| |  |  |  |  |  |  |  |  | | --- | --- | --- | --- | --- | --- | --- | --- | | | GO:0032786 1:3|3:6307 1.43e-03 1:39|3:6307 1.84e-02 | positive regulation of RNA elongation | | | | --- | --- | --- | --- | | CTK1 (YKL139W) | | Genes ausentes |  | | |

|  |  |  |  |
| --- | --- | --- | --- |
| |  |  |  | | --- | --- | --- | | | GO:0006118 | electron transport | | --- | --- | | |

|  |  |  |  |
| --- | --- | --- | --- |
| |  |  |  | | --- | --- | --- | | | GO:0042775 | organelle ATP synthesis coupled electron transport | | --- | --- | | |

|  |  |  |  |
| --- | --- | --- | --- |
| |  |  |  | | --- | --- | --- | | | GO:0042773 | ATP synthesis coupled electron transport | | --- | --- | | |

|  |  |  |  |
| --- | --- | --- | --- |
| |  |  |  | | --- | --- | --- | | | GO:0006119 | oxidative phosphorylation | | --- | --- | | |

|  |  |  |  |
| --- | --- | --- | --- |
| |  |  |  | | --- | --- | --- | | | GO:0016310 | phosphorylation | | --- | --- | | |

|  |  |  |  |
| --- | --- | --- | --- |
| |  |  |  | | --- | --- | --- | | | GO:0006796 | phosphate metabolic process | | --- | --- | | |

|  |  |  |  |  |  |  |  |  |
| --- | --- | --- | --- | --- | --- | --- | --- | --- |
| |  |  |  |  |  |  |  |  | | --- | --- | --- | --- | --- | --- | --- | --- | | | GO:0006121 1:4|4:6307 2.54e-03 1:39|4:6307 2.45e-02 | mitochondrial electron transport, succinate to ubiquinone | | | | --- | --- | --- | --- | | SDH3 (YKL141W) | | Genes ausentes |  | | |

|  |  |  |  |
| --- | --- | --- | --- |
| |  |  |  | | --- | --- | --- | | | GO:0006644 | phospholipid metabolic process | | --- | --- | | |

|  |  |  |  |
| --- | --- | --- | --- |
| |  |  |  | | --- | --- | --- | | | GO:0006665 | sphingolipid metabolic process | | --- | --- | | |

|  |  |  |  |
| --- | --- | --- | --- |
| |  |  |  | | --- | --- | --- | | | GO:0008654 | phospholipid biosynthetic process | | --- | --- | | |

|  |  |  |  |  |  |  |  |  |  |  |
| --- | --- | --- | --- | --- | --- | --- | --- | --- | --- | --- |
| |  |  |  |  |  |  |  |  |  |  | | --- | --- | --- | --- | --- | --- | --- | --- | --- | --- | | | GO:0006633 3:16|16:6307 7.35e-06 3:39|16:6307 1.16e-04 | fatty acid biosynthetic process | | | | --- | --- | --- | --- | | TPI1 (YDR050C) | | ACP1 (YKL192C) | | ACC1 (YNR016C) | | Genes ausentes |  | | |

|  |  |  |  |
| --- | --- | --- | --- |
| |  |  |  | | --- | --- | --- | | | GO:0046467 | membrane lipid biosynthetic process | | --- | --- | | |

|  |  |  |  |
| --- | --- | --- | --- |
| |  |  |  | | --- | --- | --- | | | GO:0006643 | membrane lipid metabolic process | | --- | --- | | |

|  |  |  |  |  |  |  |  |  |  |
| --- | --- | --- | --- | --- | --- | --- | --- | --- | --- |
| |  |  |  |  |  |  |  |  |  | | --- | --- | --- | --- | --- | --- | --- | --- | --- | | | GO:0030148 2:15|15:6307 5.45e-04 2:39|15:6307 3.72e-03 | sphingolipid biosynthetic process | | | | --- | --- | --- | --- | | LCB1 (YMR296C) | | LCB2 (YDR062W) | | Genes ausentes |  | | |

|  |  |  |  |
| --- | --- | --- | --- |
| |  |  |  | | --- | --- | --- | | | GO:0006351 | transcription, DNA dependent | | --- | --- | | |

|  |  |  |  |
| --- | --- | --- | --- |
| |  |  |  | | --- | --- | --- | | | GO:0032984 | macromolecular complex disassembly | | --- | --- | | |

|  |  |  |  |
| --- | --- | --- | --- |
| |  |  |  | | --- | --- | --- | | | GO:0045229 | external encapsulating structure organization and biogenesis | | --- | --- | | |

|  |  |  |  |
| --- | --- | --- | --- |
| |  |  |  | | --- | --- | --- | | | GO:0006996 | organelle organization and biogenesis | | --- | --- | | |

|  |  |  |  |
| --- | --- | --- | --- |
| |  |  |  | | --- | --- | --- | | | GO:0016044 | membrane organization and biogenesis | | --- | --- | | |

|  |  |  |  |
| --- | --- | --- | --- |
| |  |  |  | | --- | --- | --- | | | GO:0022411 | cellular component disassembly | | --- | --- | | |

|  |  |  |  |  |  |  |  |  |
| --- | --- | --- | --- | --- | --- | --- | --- | --- |
| |  |  |  |  |  |  |  |  | | --- | --- | --- | --- | --- | --- | --- | --- | | | GO:0006353 1:4|4:6307 2.54e-03 1:39|4:6307 2.45e-02 | transcription termination | | | | --- | --- | --- | --- | | GRS1 (YBR121C) | | Genes ausentes |  | | |

|  |  |  |  |
| --- | --- | --- | --- |
| |  |  |  | | --- | --- | --- | | | GO:0042723 | thiamin and derivative metabolic process | | --- | --- | | |

|  |  |  |  |
| --- | --- | --- | --- |
| |  |  |  | | --- | --- | --- | | | GO:0042726 | riboflavin and derivative metabolic process | | --- | --- | | |

|  |  |  |  |
| --- | --- | --- | --- |
| |  |  |  | | --- | --- | --- | | | GO:0042364 | water soluble vitamin biosynthetic process | | --- | --- | | |

|  |  |  |  |
| --- | --- | --- | --- |
| |  |  |  | | --- | --- | --- | | | GO:0006767 | water soluble vitamin metabolic process | | --- | --- | | |

|  |  |  |  |
| --- | --- | --- | --- |
| |  |  |  | | --- | --- | --- | | | GO:0006740 | NADPH regeneration | | --- | --- | | |

|  |  |  |  |
| --- | --- | --- | --- |
| |  |  |  | | --- | --- | --- | | | GO:0006739 | NADP metabolic process | | --- | --- | | |

|  |  |  |  |
| --- | --- | --- | --- |
| |  |  |  | | --- | --- | --- | | | GO:0006769 | nicotinamide metabolic process | | --- | --- | | |

|  |  |  |  |
| --- | --- | --- | --- |
| |  |  |  | | --- | --- | --- | | | GO:0044265 | cellular macromolecule catabolic process | | --- | --- | | |

|  |  |  |  |
| --- | --- | --- | --- |
| |  |  |  | | --- | --- | --- | | | GO:0006007 | glucose catabolic process | | --- | --- | | |

|  |  |  |  |  |  |  |  |  |  |  |
| --- | --- | --- | --- | --- | --- | --- | --- | --- | --- | --- |
| |  |  |  |  |  |  |  |  |  |  | | --- | --- | --- | --- | --- | --- | --- | --- | --- | --- | | | GO:0006098 3:14|14:6307 3.12e-06 3:39|14:6307 7.59e-05 | pentose phosphate shunt | | | | --- | --- | --- | --- | | TPI1 (YDR050C) | | PGI1 (YBR196C) | | RKI1 (YOR095C) | | Genes ausentes |  | | |

|  |  |  |  |
| --- | --- | --- | --- |
| |  |  |  | | --- | --- | --- | | | GO:0043241 | protein complex disassembly | | --- | --- | | |

|  |  |  |  |
| --- | --- | --- | --- |
| |  |  |  | | --- | --- | --- | | | GO:0009057 | macromolecule catabolic process | | --- | --- | | |

|  |  |  |  |
| --- | --- | --- | --- |
| |  |  |  | | --- | --- | --- | | | GO:0043283 | biopolymer metabolic process | | --- | --- | | |

|  |  |  |  |
| --- | --- | --- | --- |
| |  |  |  | | --- | --- | --- | | | GO:0043284 | biopolymer biosynthetic process | | --- | --- | | |

|  |  |  |  |
| --- | --- | --- | --- |
| |  |  |  | | --- | --- | --- | | | GO:0006396 | RNA processing | | --- | --- | | |

|  |  |  |  |
| --- | --- | --- | --- |
| |  |  |  | | --- | --- | --- | | | GO:0016071 | mRNA metabolic process | | --- | --- | | |

|  |  |  |  |
| --- | --- | --- | --- |
| |  |  |  | | --- | --- | --- | | | GO:0006354 | RNA elongation | | --- | --- | | |

|  |  |  |  |
| --- | --- | --- | --- |
| |  |  |  | | --- | --- | --- | | | GO:0051252 | regulation of RNA metabolic process | | --- | --- | | |

|  |  |  |  |
| --- | --- | --- | --- |
| |  |  |  | | --- | --- | --- | | | GO:0032774 | RNA biosynthetic process | | --- | --- | | |

|  |  |  |  |
| --- | --- | --- | --- |
| |  |  |  | | --- | --- | --- | | | GO:0006399 | tRNA metabolic process | | --- | --- | | |

|  |  |  |  |
| --- | --- | --- | --- |
| |  |  |  | | --- | --- | --- | | | GO:0043624 | cellular protein complex disassembly | | --- | --- | | |

|  |  |  |  |
| --- | --- | --- | --- |
| |  |  |  | | --- | --- | --- | | | GO:0044267 | cellular protein metabolic process | | --- | --- | | |

|  |  |  |  |  |  |  |  |  |
| --- | --- | --- | --- | --- | --- | --- | --- | --- |
| |  |  |  |  |  |  |  |  | | --- | --- | --- | --- | --- | --- | --- | --- | | | GO:0006428 1:2|2:6307 6.34e-04 1:39|2:6307 1.23e-02 | isoleucyl tRNA aminoacylation | | | | --- | --- | --- | --- | | ILS1 (YBL076C) | | Genes ausentes |  | | |

|  |  |  |  |  |  |  |  |  |
| --- | --- | --- | --- | --- | --- | --- | --- | --- |
| |  |  |  |  |  |  |  |  | | --- | --- | --- | --- | --- | --- | --- | --- | | | GO:0006437 1:2|2:6307 6.34e-04 1:39|2:6307 1.23e-02 | tyrosyl tRNA aminoacylation | | | | --- | --- | --- | --- | | TYS1 (YGR185C) | | Genes ausentes |  | | |

|  |  |  |  |  |  |  |  |  |  |
| --- | --- | --- | --- | --- | --- | --- | --- | --- | --- |
| |  |  |  |  |  |  |  |  |  | | --- | --- | --- | --- | --- | --- | --- | --- | --- | | | GO:0006432 2:3|3:6307 4.53e-07 2:39|3:6307 1.11e-04 | phenylalanyl tRNA aminoacylation | | | | --- | --- | --- | --- | | FRS1 (YLR060W) | | FRS2 (YFL022C) | | Genes ausentes |  | | |

|  |  |  |  |  |  |  |  |  |
| --- | --- | --- | --- | --- | --- | --- | --- | --- |
| |  |  |  |  |  |  |  |  | | --- | --- | --- | --- | --- | --- | --- | --- | | | GO:0006421 1:2|2:6307 6.34e-04 1:39|2:6307 1.23e-02 | asparaginyl tRNA aminoacylation | | | | --- | --- | --- | --- | | DED81 (YHR019C) | | Genes ausentes |  | | |

|  |  |  |  |  |  |  |  |  |
| --- | --- | --- | --- | --- | --- | --- | --- | --- |
| |  |  |  |  |  |  |  |  | | --- | --- | --- | --- | --- | --- | --- | --- | | | GO:0006426 1:2|2:6307 6.34e-04 1:39|2:6307 1.23e-02 | glycyl tRNA aminoacylation | | | | --- | --- | --- | --- | | GRS1 (YBR121C) | | Genes ausentes |  | | |

|  |  |  |  |  |  |  |  |  |
| --- | --- | --- | --- | --- | --- | --- | --- | --- |
| |  |  |  |  |  |  |  |  | | --- | --- | --- | --- | --- | --- | --- | --- | | | GO:0006434 1:2|2:6307 6.34e-04 1:39|2:6307 1.23e-02 | seryl tRNA aminoacylation | | | | --- | --- | --- | --- | | SES1 (YDR023W) | | Genes ausentes |  | | |

|  |  |  |  |  |  |  |  |  |
| --- | --- | --- | --- | --- | --- | --- | --- | --- |
| |  |  |  |  |  |  |  |  | | --- | --- | --- | --- | --- | --- | --- | --- | | | GO:0006420 1:2|2:6307 6.34e-04 1:39|2:6307 1.23e-02 | arginyl tRNA aminoacylation | | | | --- | --- | --- | --- | | YDR341C (YDR341C) | | Genes ausentes |  | | |

|  |  |  |  |  |  |  |  |  |
| --- | --- | --- | --- | --- | --- | --- | --- | --- |
| |  |  |  |  |  |  |  |  | | --- | --- | --- | --- | --- | --- | --- | --- | | | GO:0006422 1:4|4:6307 2.54e-03 1:39|4:6307 2.45e-02 | aspartyl tRNA aminoacylation | | | | --- | --- | --- | --- | | DED81 (YHR019C) | | Genes ausentes |  | | |

|  |  |  |  |  |  |  |  |  |
| --- | --- | --- | --- | --- | --- | --- | --- | --- |
| |  |  |  |  |  |  |  |  | | --- | --- | --- | --- | --- | --- | --- | --- | | | GO:0006429 1:2|2:6307 6.34e-04 1:39|2:6307 1.23e-02 | leucyl tRNA aminoacylation | | | | --- | --- | --- | --- | | CDC60 (YPL160W) | | Genes ausentes |  | | |

|  |  |  |  |  |  |  |  |  |  |  |  |  |  |  |  |  |  |  |
| --- | --- | --- | --- | --- | --- | --- | --- | --- | --- | --- | --- | --- | --- | --- | --- | --- | --- | --- |
| |  |  |  |  |  |  |  |  |  |  |  |  |  |  |  |  |  |  | | --- | --- | --- | --- | --- | --- | --- | --- | --- | --- | --- | --- | --- | --- | --- | --- | --- | --- | | | GO:0006418 12:35|35:6307 7.87e-20 12:39|35:6307 3.64e-19 | tRNA aminoacylation for protein translation | | | | --- | --- | --- | --- | | ILS1 (YBL076C) | | HTS1 (YPR033C) | | CDC60 (YPL160W) | | FRS2 (YFL022C) | | SES1 (YDR023W) | | TYS1 (YGR185C) | | DED81 (YHR019C) | | VAS1 (YGR094W) | | YDR341C (YDR341C) | | GRS1 (YBR121C) | | KRS1 (YDR037W) | | Genes ausentes |  | | |

|  |  |  |  |  |  |  |  |  |
| --- | --- | --- | --- | --- | --- | --- | --- | --- |
| |  |  |  |  |  |  |  |  | | --- | --- | --- | --- | --- | --- | --- | --- | | | GO:0043039 13:37|37:6307 2.94e-21 13:39|37:6307 6.66e-21 | tRNA aminoacylation | | | | --- | --- | --- | --- | | ALA1 (YOR335C) | | Genes ausentes |  | | |

|  |  |  |  |
| --- | --- | --- | --- |
| |  |  |  | | --- | --- | --- | | | GO:0009069 | serine family amino acid metabolic process | | --- | --- | | |

|  |  |  |  |
| --- | --- | --- | --- |
| |  |  |  | | --- | --- | --- | | | GO:0008652 | amino acid biosynthetic process | | --- | --- | | |

|  |  |  |  |
| --- | --- | --- | --- |
| |  |  |  | | --- | --- | --- | | | GO:0043038 | amino acid activation | | --- | --- | | |

|  |  |  |  |
| --- | --- | --- | --- |
| |  |  |  | | --- | --- | --- | | | GO:0009309 | amine biosynthetic process | | --- | --- | | |

|  |  |  |  |  |  |  |  |  |
| --- | --- | --- | --- | --- | --- | --- | --- | --- |
| |  |  |  |  |  |  |  |  | | --- | --- | --- | --- | --- | --- | --- | --- | | | GO:0006430 1:2|2:6307 6.34e-04 1:39|2:6307 1.23e-02 | lysyl tRNA aminoacylation | | | | --- | --- | --- | --- | | KRS1 (YDR037W) | | Genes ausentes |  | | |

|  |  |  |  |
| --- | --- | --- | --- |
| |  |  |  | | --- | --- | --- | | | GO:0046351 | disaccharide biosynthetic process | | --- | --- | | |

|  |  |  |  |
| --- | --- | --- | --- |
| |  |  |  | | --- | --- | --- | | | GO:0000271 | polysaccharide biosynthetic process | | --- | --- | | |

|  |  |  |  |
| --- | --- | --- | --- |
| |  |  |  | | --- | --- | --- | | | GO:0005976 | polysaccharide metabolic process | | --- | --- | | |

|  |  |  |  |
| --- | --- | --- | --- |
| |  |  |  | | --- | --- | --- | | | GO:0016052 | carbohydrate catabolic process | | --- | --- | | |

|  |  |  |  |
| --- | --- | --- | --- |
| |  |  |  | | --- | --- | --- | | | GO:0016051 | carbohydrate biosynthetic process | | --- | --- | | |

|  |  |  |  |  |  |  |  |  |
| --- | --- | --- | --- | --- | --- | --- | --- | --- |
| |  |  |  |  |  |  |  |  | | --- | --- | --- | --- | --- | --- | --- | --- | | | GO:0006011 1:3|3:6307 1.43e-03 1:39|3:6307 1.84e-02 | UDP glucose metabolic process | | | | --- | --- | --- | --- | | UGP1 (YKL035W) | | Genes ausentes |  | | |

|  |  |  |  |
| --- | --- | --- | --- |
| |  |  |  | | --- | --- | --- | | | GO:0005984 | disaccharide metabolic process | | --- | --- | | |

|  |  |  |  |
| --- | --- | --- | --- |
| |  |  |  | | --- | --- | --- | | | GO:0044264 | cellular polysaccharide metabolic process | | --- | --- | | |

|  |  |  |  |
| --- | --- | --- | --- |
| |  |  |  | | --- | --- | --- | | | GO:0044275 | cellular carbohydrate catabolic process | | --- | --- | | |

|  |  |  |  |
| --- | --- | --- | --- |
| |  |  |  | | --- | --- | --- | | | GO:0019673 | GDP mannose metabolic process | | --- | --- | | |

|  |  |  |  |
| --- | --- | --- | --- |
| |  |  |  | | --- | --- | --- | | | GO:0006006 | glucose metabolic process | | --- | --- | | |

|  |  |  |  |
| --- | --- | --- | --- |
| |  |  |  | | --- | --- | --- | | | GO:0019320 | hexose catabolic process | | --- | --- | | |

|  |  |  |  |
| --- | --- | --- | --- |
| |  |  |  | | --- | --- | --- | | | GO:0006013 | mannose metabolic process | | --- | --- | | |

|  |  |  |  |
| --- | --- | --- | --- |
| |  |  |  | | --- | --- | --- | | | GO:0046365 | monosaccharide catabolic process | | --- | --- | | |

|  |  |  |  |
| --- | --- | --- | --- |
| |  |  |  | | --- | --- | --- | | | GO:0019318 | hexose metabolic process | | --- | --- | | |

|  |  |  |  |  |  |  |  |  |  |
| --- | --- | --- | --- | --- | --- | --- | --- | --- | --- |
| |  |  |  |  |  |  |  |  |  | | --- | --- | --- | --- | --- | --- | --- | --- | --- | | | GO:0006094 2:19|19:6307 1.43e-03 2:39|19:6307 5.96e-03 | gluconeogenesis | | | | --- | --- | --- | --- | | TPI1 (YDR050C) | | PGI1 (YBR196C) | | Genes ausentes |  | | |

|  |  |  |  |
| --- | --- | --- | --- |
| |  |  |  | | --- | --- | --- | | | GO:0019307 | mannose biosynthetic process | | --- | --- | | |

|  |  |  |  |
| --- | --- | --- | --- |
| |  |  |  | | --- | --- | --- | | | GO:0019319 | hexose biosynthetic process | | --- | --- | | |

|  |  |  |  |
| --- | --- | --- | --- |
| |  |  |  | | --- | --- | --- | | | GO:0046364 | monosaccharide biosynthetic process | | --- | --- | | |

|  |  |  |  |
| --- | --- | --- | --- |
| |  |  |  | | --- | --- | --- | | | GO:0046164 | alcohol catabolic process | | --- | --- | | |

|  |  |  |  |
| --- | --- | --- | --- |
| |  |  |  | | --- | --- | --- | | | GO:0005996 | monosaccharide metabolic process | | --- | --- | | |

|  |  |  |  |  |  |  |  |  |
| --- | --- | --- | --- | --- | --- | --- | --- | --- |
| |  |  |  |  |  |  |  |  | | --- | --- | --- | --- | --- | --- | --- | --- | | | GO:0009298 1:2|2:6307 6.34e-04 1:39|2:6307 1.23e-02 | GDP mannose biosynthetic process | | | | --- | --- | --- | --- | | PSA1 (YDL055C) | | Genes ausentes |  | | |

|  |  |  |  |
| --- | --- | --- | --- |
| |  |  |  | | --- | --- | --- | | | GO:0006629 | lipid metabolic process | | --- | --- | | |

|  |  |  |  |
| --- | --- | --- | --- |
| |  |  |  | | --- | --- | --- | | | GO:0019538 | protein metabolic process | | --- | --- | | |

|  |  |  |  |
| --- | --- | --- | --- |
| |  |  |  | | --- | --- | --- | | | GO:0005975 | carbohydrate metabolic process | | --- | --- | | |

|  |  |  |  |
| --- | --- | --- | --- |
| |  |  |  | | --- | --- | --- | | | GO:0008610 | lipid biosynthetic process | | --- | --- | | |

|  |  |  |  |
| --- | --- | --- | --- |
| |  |  |  | | --- | --- | --- | | | GO:0009059 | macromolecule biosynthetic process | | --- | --- | | |

|  |  |  |  |
| --- | --- | --- | --- |
| |  |  |  | | --- | --- | --- | | | GO:0019362 | pyridine nucleotide metabolic process | | --- | --- | | |

|  |  |  |  |  |  |  |  |  |
| --- | --- | --- | --- | --- | --- | --- | --- | --- |
| |  |  |  |  |  |  |  |  | | --- | --- | --- | --- | --- | --- | --- | --- | | | GO:0009117 3:19|19:6307 2.18e-05 3:39|19:6307 1.98e-04 | nucleotide metabolic process | | | | --- | --- | --- | --- | | DUT1 (YBR252W) | | Genes ausentes |  | | |

|  |  |  |  |
| --- | --- | --- | --- |
| |  |  |  | | --- | --- | --- | | | GO:0006753 | nucleoside phosphate metabolic process | | --- | --- | | |

|  |  |  |  |
| --- | --- | --- | --- |
| |  |  |  | | --- | --- | --- | | | GO:0019219 | regulation of nucleobase, nucleoside, nucleotide and nucleic acid metabolic process | | --- | --- | | |

|  |  |  |  |
| --- | --- | --- | --- |
| |  |  |  | | --- | --- | --- | | | GO:0006350 | transcription | | --- | --- | | |

|  |  |  |  |
| --- | --- | --- | --- |
| |  |  |  | | --- | --- | --- | | | GO:0016070 | RNA metabolic process | | --- | --- | | |

|  |  |  |  |
| --- | --- | --- | --- |
| |  |  |  | | --- | --- | --- | | | GO:0009225 | nucleotide sugar metabolic process | | --- | --- | | |

|  |  |  |  |
| --- | --- | --- | --- |
| |  |  |  | | --- | --- | --- | | | GO:0055086 | nucleobase, nucleoside and nucleotide metabolic process | | --- | --- | | |

|  |  |  |  |
| --- | --- | --- | --- |
| |  |  |  | | --- | --- | --- | | | GO:0044271 | nitrogen compound biosynthetic process | | --- | --- | | |

|  |  |  |  |
| --- | --- | --- | --- |
| |  |  |  | | --- | --- | --- | | | GO:0009110 | vitamin biosynthetic process | | --- | --- | | |

|  |  |  |  |
| --- | --- | --- | --- |
| |  |  |  | | --- | --- | --- | | | GO:0006412 | translation | | --- | --- | | |

|  |  |  |  |
| --- | --- | --- | --- |
| |  |  |  | | --- | --- | --- | | | GO:0009226 | nucleotide sugar biosynthetic process | | --- | --- | | |

|  |  |  |  |
| --- | --- | --- | --- |
| |  |  |  | | --- | --- | --- | | | GO:0046165 | alcohol biosynthetic process | | --- | --- | | |

|  |  |  |  |  |  |  |  |  |  |
| --- | --- | --- | --- | --- | --- | --- | --- | --- | --- |
| |  |  |  |  |  |  |  |  |  | | --- | --- | --- | --- | --- | --- | --- | --- | --- | | | GO:0009165 2:9|9:6307 6.48e-05 2:39|9:6307 1.31e-03 | nucleotide biosynthetic process | | | | --- | --- | --- | --- | | DFR1 (YOR236W) | | CDC8 (YJR057W) | | Genes ausentes |  | | |

|  |  |  |  |
| --- | --- | --- | --- |
| |  |  |  | | --- | --- | --- | | | GO:0019222 | regulation of metabolic process | | --- | --- | | |

|  |  |  |  |
| --- | --- | --- | --- |
| |  |  |  | | --- | --- | --- | | | GO:0006091 | generation of precursor metabolites and energy | | --- | --- | | |

|  |  |  |  |
| --- | --- | --- | --- |
| |  |  |  | | --- | --- | --- | | | GO:0009056 | catabolic process | | --- | --- | | |

|  |  |  |  |
| --- | --- | --- | --- |
| |  |  |  | | --- | --- | --- | | | GO:0006807 | nitrogen compound metabolic process | | --- | --- | | |

|  |  |  |  |
| --- | --- | --- | --- |
| |  |  |  | | --- | --- | --- | | | GO:0043170 | macromolecule metabolic process | | --- | --- | | |

|  |  |  |  |
| --- | --- | --- | --- |
| |  |  |  | | --- | --- | --- | | | GO:0044238 | primary metabolic process | | --- | --- | | |

|  |  |  |  |
| --- | --- | --- | --- |
| |  |  |  | | --- | --- | --- | | | GO:0009058 | biosynthetic process | | --- | --- | | |

|  |  |  |  |
| --- | --- | --- | --- |
| |  |  |  | | --- | --- | --- | | | GO:0006090 | pyruvate metabolic process | | --- | --- | | |

|  |  |  |  |
| --- | --- | --- | --- |
| |  |  |  | | --- | --- | --- | | | GO:0006631 | fatty acid metabolic process | | --- | --- | | |

|  |  |  |  |
| --- | --- | --- | --- |
| |  |  |  | | --- | --- | --- | | | GO:0046394 | carboxylic acid biosynthetic process | | --- | --- | | |

|  |  |  |  |
| --- | --- | --- | --- |
| |  |  |  | | --- | --- | --- | | | GO:0006520 | amino acid metabolic process | | --- | --- | | |

|  |  |  |  |
| --- | --- | --- | --- |
| |  |  |  | | --- | --- | --- | | | GO:0032787 | monocarboxylic acid metabolic process | | --- | --- | | |

|  |  |  |  |
| --- | --- | --- | --- |
| |  |  |  | | --- | --- | --- | | | GO:0016053 | organic acid biosynthetic process | | --- | --- | | |

|  |  |  |  |
| --- | --- | --- | --- |
| |  |  |  | | --- | --- | --- | | | GO:0019752 | carboxylic acid metabolic process | | --- | --- | | |

|  |  |  |  |
| --- | --- | --- | --- |
| |  |  |  | | --- | --- | --- | | | GO:0000273 | lipoic acid metabolic process | | --- | --- | | |

|  |  |  |  |
| --- | --- | --- | --- |
| |  |  |  | | --- | --- | --- | | | GO:0009108 | coenzyme biosynthetic process | | --- | --- | | |

|  |  |  |  |
| --- | --- | --- | --- |
| |  |  |  | | --- | --- | --- | | | GO:0006733 | oxidoreduction coenzyme metabolic process | | --- | --- | | |

|  |  |  |  |
| --- | --- | --- | --- |
| |  |  |  | | --- | --- | --- | | | GO:0006752 | group transfer coenzyme metabolic process | | --- | --- | | |

|  |  |  |  |
| --- | --- | --- | --- |
| |  |  |  | | --- | --- | --- | | | GO:0051188 | cofactor biosynthetic process | | --- | --- | | |

|  |  |  |  |
| --- | --- | --- | --- |
| |  |  |  | | --- | --- | --- | | | GO:0006732 | coenzyme metabolic process | | --- | --- | | |

|  |  |  |  |
| --- | --- | --- | --- |
| |  |  |  | | --- | --- | --- | | | GO:0009112 | nucleobase metabolic process | | --- | --- | | |

|  |  |  |  |
| --- | --- | --- | --- |
| |  |  |  | | --- | --- | --- | | | GO:0043094 | metabolic compound salvage | | --- | --- | | |

|  |  |  |  |
| --- | --- | --- | --- |
| |  |  |  | | --- | --- | --- | | | GO:0006800 | oxygen and reactive oxygen species metabolic process | | --- | --- | | |

|  |  |  |  |
| --- | --- | --- | --- |
| |  |  |  | | --- | --- | --- | | | GO:0031323 | regulation of cellular metabolic process | | --- | --- | | |

|  |  |  |  |
| --- | --- | --- | --- |
| |  |  |  | | --- | --- | --- | | | GO:0006793 | phosphorus metabolic process | | --- | --- | | |

|  |  |  |  |
| --- | --- | --- | --- |
| |  |  |  | | --- | --- | --- | | | GO:0044255 | cellular lipid metabolic process | | --- | --- | | |

|  |  |  |  |
| --- | --- | --- | --- |
| |  |  |  | | --- | --- | --- | | | GO:0006766 | vitamin metabolic process | | --- | --- | | |

|  |  |  |  |
| --- | --- | --- | --- |
| |  |  |  | | --- | --- | --- | | | GO:0044248 | cellular catabolic process | | --- | --- | | |

|  |  |  |  |
| --- | --- | --- | --- |
| |  |  |  | | --- | --- | --- | | | GO:0044260 | cellular macromolecule metabolic process | | --- | --- | | |

|  |  |  |  |
| --- | --- | --- | --- |
| |  |  |  | | --- | --- | --- | | | GO:0006519 | amino acid and derivative metabolic process | | --- | --- | | |

|  |  |  |  |
| --- | --- | --- | --- |
| |  |  |  | | --- | --- | --- | | | GO:0009308 | amine metabolic process | | --- | --- | | |

|  |  |  |  |
| --- | --- | --- | --- |
| |  |  |  | | --- | --- | --- | | | GO:0044262 | cellular carbohydrate metabolic process | | --- | --- | | |

|  |  |  |  |
| --- | --- | --- | --- |
| |  |  |  | | --- | --- | --- | | | GO:0006066 | alcohol metabolic process | | --- | --- | | |

|  |  |  |  |  |  |  |  |  |  |  |
| --- | --- | --- | --- | --- | --- | --- | --- | --- | --- | --- |
| |  |  |  |  |  |  |  |  |  |  | | --- | --- | --- | --- | --- | --- | --- | --- | --- | --- | | | GO:0006730 3:15|15:6307 4.87e-06 3:39|15:6307 9.45e-05 | one carbon compound metabolic process | | | | --- | --- | --- | --- | | SAH1 (YER043C) | | DFR1 (YOR236W) | | FOL3 (YMR113W) | | Genes ausentes |  | | |

|  |  |  |  |
| --- | --- | --- | --- |
| |  |  |  | | --- | --- | --- | | | GO:0006139 | nucleobase, nucleoside, nucleotide and nucleic acid metabolic process | | --- | --- | | |

|  |  |  |  |
| --- | --- | --- | --- |
| |  |  |  | | --- | --- | --- | | | GO:0044249 | cellular biosynthetic process | | --- | --- | | |

|  |  |  |  |
| --- | --- | --- | --- |
| |  |  |  | | --- | --- | --- | | | GO:0006082 | organic acid metabolic process | | --- | --- | | |

|  |  |  |  |
| --- | --- | --- | --- |
| |  |  |  | | --- | --- | --- | | | GO:0046483 | heterocycle metabolic process | | --- | --- | | |

|  |  |  |  |
| --- | --- | --- | --- |
| |  |  |  | | --- | --- | --- | | | GO:0051186 | cofactor metabolic process | | --- | --- | | |

|  |  |  |  |
| --- | --- | --- | --- |
| |  |  |  | | --- | --- | --- | | | GO:0006725 | aromatic compound metabolic process | | --- | --- | | |

|  |  |  |  |  |  |  |  |  |
| --- | --- | --- | --- | --- | --- | --- | --- | --- |
| |  |  |  |  |  |  |  |  | | --- | --- | --- | --- | --- | --- | --- | --- | | | GO:0006276 1:8|8:6307 1.01e-02 1:39|8:6307 4.84e-02 | plasmid maintenance | | | | --- | --- | --- | --- | | CDC8 (YJR057W) | | Genes ausentes |  | | |

|  |  |  |  |
| --- | --- | --- | --- |
| |  |  |  | | --- | --- | --- | | | GO:0050794 | regulation of cellular process | | --- | --- | | |

|  |  |  |  |
| --- | --- | --- | --- |
| |  |  |  | | --- | --- | --- | | | GO:0016043 | cellular component organization and biogenesis | | --- | --- | | |

|  |  |  |  |
| --- | --- | --- | --- |
| |  |  |  | | --- | --- | --- | | | GO:0044237 | cellular metabolic process | | --- | --- | | |

|  |  |  |  |
| --- | --- | --- | --- |
| |  |  |  | | --- | --- | --- | | | GO:0065007 | biological regulation | | --- | --- | | |

|  |  |  |  |
| --- | --- | --- | --- |
| |  |  |  | | --- | --- | --- | | | GO:0010467 | gene expression | | --- | --- | | |

|  |  |  |  |
| --- | --- | --- | --- |
| |  |  |  | | --- | --- | --- | | | GO:0008152 | metabolic process | | --- | --- | | |

|  |  |  |  |
| --- | --- | --- | --- |
| |  |  |  | | --- | --- | --- | | | GO:0009987 | cellular process | | --- | --- | | |

|  |  |  |  |
| --- | --- | --- | --- |
| |  |  |  | | --- | --- | --- | | | GO:0008150 | biological\_process | | --- | --- | | |

|  |  |  |  |
| --- | --- | --- | --- |
| |  |  |  | | --- | --- | --- | | | GO:0003673 | Gene\_Ontology | | --- | --- | | |

|  |  |  |  |  |  |  |  |  |
| --- | --- | --- | --- | --- | --- | --- | --- | --- |
| |  |  |  |  |  |  |  |  | | --- | --- | --- | --- | --- | --- | --- | --- | | | GO:0006760 1:3|3:6307 1.43e-03 1:39|3:6307 1.84e-02 | folic acid and derivative metabolic process | | | | --- | --- | --- | --- | | DFR1 (YOR236W) | | Genes ausentes |  | | |
