# Supplementary material for: How to identify essential genes from molecular networks?
Source: BMC Syst Biol. 2009 Oct 13;3:102. doi: 10.1186/1752-0509-3-102 (PMC2765966; doi:10.1186/1752-0509-3-102)
Supplement: Additional file 2 — Figure S1. Unpredicted essential metabolic genes matching GO classification with locally essential genes. [file 1752-0509-3-102-S2.GZ › MF_glogal_with_coincidencias.html]

|  |  |  |  |  |  |
| --- | --- | --- | --- | --- | --- |
| |  | | --- | | **RER2** |  |  |  |  |  | | --- | --- | --- | --- | | |  | | --- | | SGD | | GRID | | KEGG | | |


|  |  |  |  |
| --- | --- | --- | --- |
| |  |  |  | | --- | --- | --- | | | GO:0016728 | oxidoreductase activity, acting on CH or CH2 groups, disulfide as acceptor | | --- | --- | | |

|  |  |  |  |
| --- | --- | --- | --- |
| |  |  |  | | --- | --- | --- | | | GO:0016725 | oxidoreductase activity, acting on CH or CH2 groups | | --- | --- | | |

|  |  |  |  |  |  |  |  |  |
| --- | --- | --- | --- | --- | --- | --- | --- | --- |
| |  |  |  |  |  |  |  |  | | --- | --- | --- | --- | --- | --- | --- | --- | | | GO:0004748 1:4|4:6306 2.54e-03 1:72|4:6306 4.49e-02 | ribonucleoside diphosphate reductase activity | | | | --- | --- | --- | --- | | RNR2 (YJL026W) | | Genes ausentes |  | | |

|  |  |  |  |
| --- | --- | --- | --- |
| |  |  |  | | --- | --- | --- | | | GO:0008483 | transaminase activity | | --- | --- | | |

|  |  |  |  |  |  |  |  |  |
| --- | --- | --- | --- | --- | --- | --- | --- | --- |
| |  |  |  |  |  |  |  |  | | --- | --- | --- | --- | --- | --- | --- | --- | | | GO:0004360 1:2|2:6306 6.34e-04 1:72|2:6306 2.27e-02 | glutamine fructose 6 phosphate transaminase (isomerizing) activity | | | | --- | --- | --- | --- | | GFA1 (YKL104C) | | Genes ausentes |  | | |

|  |  |  |  |
| --- | --- | --- | --- |
| |  |  |  | | --- | --- | --- | | | GO:0016405 | CoA ligase activity | | --- | --- | | |

|  |  |  |  |
| --- | --- | --- | --- |
| |  |  |  | | --- | --- | --- | | | GO:0016878 | acid thiol ligase activity | | --- | --- | | |

|  |  |  |  |  |  |  |  |  |
| --- | --- | --- | --- | --- | --- | --- | --- | --- |
| |  |  |  |  |  |  |  |  | | --- | --- | --- | --- | --- | --- | --- | --- | | | GO:0003987 1:2|2:6306 6.34e-04 1:72|2:6306 2.27e-02 | acetate CoA ligase activity | | | | --- | --- | --- | --- | | ACS2 (YLR153C) | | Genes ausentes |  | | |

|  |  |  |  |
| --- | --- | --- | --- |
| |  |  |  | | --- | --- | --- | | | GO:0016796 | exonuclease activity, active with either ribo or deoxyribonucleic acids and producing 5 phosphomonoesters | | --- | --- | | |

|  |  |  |  |
| --- | --- | --- | --- |
| |  |  |  | | --- | --- | --- | | | GO:0008408 | 3 5 exonuclease activity | | --- | --- | | |

|  |  |  |  |
| --- | --- | --- | --- |
| |  |  |  | | --- | --- | --- | | | GO:0008297 | single stranded DNA specific exodeoxyribonuclease activity | | --- | --- | | |

|  |  |  |  |
| --- | --- | --- | --- |
| |  |  |  | | --- | --- | --- | | | GO:0008296 | 3 5 exodeoxyribonuclease activity | | --- | --- | | |

|  |  |  |  |
| --- | --- | --- | --- |
| |  |  |  | | --- | --- | --- | | | GO:0016895 | exodeoxyribonuclease activity, producing 5 phosphomonoesters | | --- | --- | | |

|  |  |  |  |
| --- | --- | --- | --- |
| |  |  |  | | --- | --- | --- | | | GO:0004529 | exodeoxyribonuclease activity | | --- | --- | | |

|  |  |  |  |
| --- | --- | --- | --- |
| |  |  |  | | --- | --- | --- | | | GO:0004527 | exonuclease activity | | --- | --- | | |

|  |  |  |  |
| --- | --- | --- | --- |
| |  |  |  | | --- | --- | --- | | | GO:0004536 | deoxyribonuclease activity | | --- | --- | | |

|  |  |  |  |
| --- | --- | --- | --- |
| |  |  |  | | --- | --- | --- | | | GO:0004518 | nuclease activity | | --- | --- | | |

|  |  |  |  |  |  |  |  |  |  |
| --- | --- | --- | --- | --- | --- | --- | --- | --- | --- |
| |  |  |  |  |  |  |  |  |  | | --- | --- | --- | --- | --- | --- | --- | --- | --- | | | GO:0008310 2:3|3:6306 4.53e-07 2:72|3:6306 3.83e-04 | single stranded DNA specific 3 5 exodeoxyribonuclease activity | | | | --- | --- | --- | --- | | POL31 (YJR006W) | | POL3 (YDL102W) | | Genes ausentes |  | | |

|  |  |  |  |
| --- | --- | --- | --- |
| |  |  |  | | --- | --- | --- | | | GO:0008094 | DNA dependent ATPase activity | | --- | --- | | |

|  |  |  |  |
| --- | --- | --- | --- |
| |  |  |  | | --- | --- | --- | | | GO:0042623 | ATPase activity, coupled | | --- | --- | | |

|  |  |  |  |
| --- | --- | --- | --- |
| |  |  |  | | --- | --- | --- | | | GO:0016887 | ATPase activity | | --- | --- | | |

|  |  |  |  |
| --- | --- | --- | --- |
| |  |  |  | | --- | --- | --- | | | GO:0017111 | nucleoside triphosphatase activity | | --- | --- | | |

|  |  |  |  |
| --- | --- | --- | --- |
| |  |  |  | | --- | --- | --- | | | GO:0016462 | pyrophosphatase activity | | --- | --- | | |

|  |  |  |  |
| --- | --- | --- | --- |
| |  |  |  | | --- | --- | --- | | | GO:0016818 | hydrolase activity, acting on acid anhydrides, in phosphorus containing anhydrides | | --- | --- | | |

|  |  |  |  |
| --- | --- | --- | --- |
| |  |  |  | | --- | --- | --- | | | GO:0043139 | 5 3 DNA helicase activity | | --- | --- | | |

|  |  |  |  |
| --- | --- | --- | --- |
| |  |  |  | | --- | --- | --- | | | GO:0004003 | ATP dependent DNA helicase activity | | --- | --- | | |

|  |  |  |  |
| --- | --- | --- | --- |
| |  |  |  | | --- | --- | --- | | | GO:0003678 | DNA helicase activity | | --- | --- | | |

|  |  |  |  |
| --- | --- | --- | --- |
| |  |  |  | | --- | --- | --- | | | GO:0008026 | ATP dependent helicase activity | | --- | --- | | |

|  |  |  |  |  |  |  |  |  |
| --- | --- | --- | --- | --- | --- | --- | --- | --- |
| |  |  |  |  |  |  |  |  | | --- | --- | --- | --- | --- | --- | --- | --- | | | GO:0043141 1:3|3:6306 1.43e-03 1:72|3:6306 3.39e-02 | ATP dependent 5 3 DNA helicase activity | | | | --- | --- | --- | --- | | RAD3 (YER171W) | | Genes ausentes |  | | |

|  |  |  |  |
| --- | --- | --- | --- |
| |  |  |  | | --- | --- | --- | | | GO:0032559 | adenyl ribonucleotide binding | | --- | --- | | |

|  |  |  |  |
| --- | --- | --- | --- |
| |  |  |  | | --- | --- | --- | | | GO:0032555 | purine ribonucleotide binding | | --- | --- | | |

|  |  |  |  |
| --- | --- | --- | --- |
| |  |  |  | | --- | --- | --- | | | GO:0030554 | adenyl nucleotide binding | | --- | --- | | |

|  |  |  |  |
| --- | --- | --- | --- |
| |  |  |  | | --- | --- | --- | | | GO:0032553 | ribonucleotide binding | | --- | --- | | |

|  |  |  |  |
| --- | --- | --- | --- |
| |  |  |  | | --- | --- | --- | | | GO:0017076 | purine nucleotide binding | | --- | --- | | |

|  |  |  |  |  |  |  |  |  |
| --- | --- | --- | --- | --- | --- | --- | --- | --- |
| |  |  |  |  |  |  |  |  | | --- | --- | --- | --- | --- | --- | --- | --- | | | GO:0016208 1:3|3:6306 1.43e-03 1:72|3:6306 3.39e-02 | AMP binding | | | | --- | --- | --- | --- | | ACS2 (YLR153C) | | Genes ausentes |  | | |

|  |  |  |  |
| --- | --- | --- | --- |
| |  |  |  | | --- | --- | --- | | | GO:0034062 | RNA polymerase activity | | --- | --- | | |

|  |  |  |  |  |  |  |  |  |  |
| --- | --- | --- | --- | --- | --- | --- | --- | --- | --- |
| |  |  |  |  |  |  |  |  |  | | --- | --- | --- | --- | --- | --- | --- | --- | --- | | | GO:0003896 2:2|2:6306 5.03e-08 2:72|2:6306 1.29e-04 | DNA primase activity | | | | --- | --- | --- | --- | | PRI2 (YKL045W) | | PRI1 (YIR008C) | | Genes ausentes |  | | |

|  |  |  |  |  |  |  |  |  |  |  |  |  |  |  |  |  |  |  |  |  |  |  |  |  |  |  |  |  |  |  |  |  |
| --- | --- | --- | --- | --- | --- | --- | --- | --- | --- | --- | --- | --- | --- | --- | --- | --- | --- | --- | --- | --- | --- | --- | --- | --- | --- | --- | --- | --- | --- | --- | --- | --- |
| |  |  |  |  |  |  |  |  |  |  |  |  |  |  |  |  |  |  |  |  |  |  |  |  |  |  |  |  |  |  |  |  | | --- | --- | --- | --- | --- | --- | --- | --- | --- | --- | --- | --- | --- | --- | --- | --- | --- | --- | --- | --- | --- | --- | --- | --- | --- | --- | --- | --- | --- | --- | --- | --- | | | GO:0003899 25:34|34:6306 4.48e-55 25:72|34:6306 1.24e-43 | DNA directed RNA polymerase activity | | | | --- | --- | --- | --- | | RPC34 (YNR003C) | | RPC31 (YNL151C) | | RPB11 (YOL005C) | | RPB3 (YIL021W) | | RPB5 (YBR154C) | | RPC10 (YHR143W-A) | | RPO31 (YOR116C) | | RPA135 (YPR010C) | | RPC19 (YNL113W) | | RPC40 (YPR110C) | | PRI2 (YKL045W) | | PRI1 (YIR008C) | | RPB2 (YOR151C) | | RPA43 (YOR340C) | | RPC11 (YDR045C) | | RPO21 (YDL140C) | | RPC82 (YPR190C) | | RPO26 (YPR187W) | | RPC25 (YKL144C) | | RPB8 (YOR224C) | | RPB7 (YDR404C) | | RPB10 (YOR210W) | | RPA190 (YOR341W) | | RPC53 (YDL150W) | | RET1 (YOR207C) | | Genes ausentes |  | | |

|  |  |  |  |  |  |  |  |  |
| --- | --- | --- | --- | --- | --- | --- | --- | --- |
| |  |  |  |  |  |  |  |  | | --- | --- | --- | --- | --- | --- | --- | --- | | | GO:0004325 1:2|2:6306 6.34e-04 1:72|2:6306 2.27e-02 | ferrochelatase activity | | | | --- | --- | --- | --- | | HEM15 (YOR176W) | | Genes ausentes |  | | |

|  |  |  |  |  |  |  |  |  |
| --- | --- | --- | --- | --- | --- | --- | --- | --- |
| |  |  |  |  |  |  |  |  | | --- | --- | --- | --- | --- | --- | --- | --- | | | GO:0016303 1:3|3:6306 1.43e-03 1:72|3:6306 3.39e-02 | 1 phosphatidylinositol 3 kinase activity | | | | --- | --- | --- | --- | | TOR2 (YKL203C) | | Genes ausentes |  | | |

|  |  |  |  |
| --- | --- | --- | --- |
| |  |  |  | | --- | --- | --- | | | GO:0000166 | nucleotide binding | | --- | --- | | |

|  |  |  |  |
| --- | --- | --- | --- |
| |  |  |  | | --- | --- | --- | | | GO:0005515 | protein binding | | --- | --- | | |

|  |  |  |  |  |  |  |  |  |  |  |  |
| --- | --- | --- | --- | --- | --- | --- | --- | --- | --- | --- | --- |
| |  |  |  |  |  |  |  |  |  |  |  | | --- | --- | --- | --- | --- | --- | --- | --- | --- | --- | --- | | | GO:0046983 4:24|24:6306 1.63e-06 4:72|24:6306 1.40e-04 | protein dimerization activity | | | | --- | --- | --- | --- | | RPB11 (YOL005C) | | RPB3 (YIL021W) | | RPC19 (YNL113W) | | RPC40 (YPR110C) | | Genes ausentes |  | | |

|  |  |  |  |  |  |  |  |  |  |  |
| --- | --- | --- | --- | --- | --- | --- | --- | --- | --- | --- |
| |  |  |  |  |  |  |  |  |  |  | | --- | --- | --- | --- | --- | --- | --- | --- | --- | --- | | | GO:0004430 3:4|4:6306 3.83e-10 3:72|4:6306 5.66e-06 | 1 phosphatidylinositol 4 kinase activity | | | | --- | --- | --- | --- | | PIK1 (YNL267W) | | STT4 (YLR305C) | | TOR2 (YKL203C) | | Genes ausentes |  | | |

|  |  |  |  |  |  |  |  |  |
| --- | --- | --- | --- | --- | --- | --- | --- | --- |
| |  |  |  |  |  |  |  |  | | --- | --- | --- | --- | --- | --- | --- | --- | | | GO:0016307 1:2|2:6306 6.34e-04 1:72|2:6306 2.27e-02 | phosphatidylinositol phosphate kinase activity | | | | --- | --- | --- | --- | | MSS4 (YDR208W) | | Genes ausentes |  | | |

|  |  |  |  |
| --- | --- | --- | --- |
| |  |  |  | | --- | --- | --- | | | GO:0035004 | phosphoinositide 3 kinase activity | | --- | --- | | |

|  |  |  |  |
| --- | --- | --- | --- |
| |  |  |  | | --- | --- | --- | | | GO:0001727 | lipid kinase activity | | --- | --- | | |

|  |  |  |  |  |  |  |  |  |  |
| --- | --- | --- | --- | --- | --- | --- | --- | --- | --- |
| |  |  |  |  |  |  |  |  |  | | --- | --- | --- | --- | --- | --- | --- | --- | --- | | | GO:0004428 4:7|7:6306 1.86e-11 4:72|7:6306 5.33e-07 | inositol or phosphatidylinositol kinase activity | | | | --- | --- | --- | --- | | PIK1 (YNL267W) | | STT4 (YLR305C) | | Genes ausentes |  | | |

|  |  |  |  |  |  |  |  |  |  |  |  |  |  |  |  |  |  |  |  |
| --- | --- | --- | --- | --- | --- | --- | --- | --- | --- | --- | --- | --- | --- | --- | --- | --- | --- | --- | --- |
| |  |  |  |  |  |  |  |  |  |  |  |  |  |  |  |  |  |  |  | | --- | --- | --- | --- | --- | --- | --- | --- | --- | --- | --- | --- | --- | --- | --- | --- | --- | --- | --- | | | GO:0016779 12:94|94:6306 1.03e-08 12:72|94:6306 4.34e-10 | nucleotidyltransferase activity | | | | --- | --- | --- | --- | | RPC31 (YNL151C) | | POL31 (YJR006W) | | DPB2 (YPR175W) | | RPO31 (YOR116C) | | RPA135 (YPR010C) | | POL3 (YDL102W) | | PRI2 (YKL045W) | | PRI1 (YIR008C) | | RPB2 (YOR151C) | | RPO21 (YDL140C) | | RPA190 (YOR341W) | | RET1 (YOR207C) | | Genes ausentes |  | | |

|  |  |  |  |  |  |  |  |  |  |  |  |
| --- | --- | --- | --- | --- | --- | --- | --- | --- | --- | --- | --- |
| |  |  |  |  |  |  |  |  |  |  |  | | --- | --- | --- | --- | --- | --- | --- | --- | --- | --- | --- | | | GO:0016773 6:19|19:6306 8.26e-12 6:72|19:6306 4.33e-08 | phosphotransferase activity, alcohol group as acceptor | | | | --- | --- | --- | --- | | PIK1 (YNL267W) | | STT4 (YLR305C) | | ERG12 (YMR208W) | | TOR2 (YKL203C) | | Genes ausentes |  | | |

|  |  |  |  |
| --- | --- | --- | --- |
| |  |  |  | | --- | --- | --- | | | GO:0016301 | kinase activity | | --- | --- | | |

|  |  |  |  |  |  |  |  |  |
| --- | --- | --- | --- | --- | --- | --- | --- | --- |
| |  |  |  |  |  |  |  |  | | --- | --- | --- | --- | --- | --- | --- | --- | | | GO:0004743 1:2|2:6306 6.34e-04 1:72|2:6306 2.27e-02 | pyruvate kinase activity | | | | --- | --- | --- | --- | | CDC19 (YAL038W) | | Genes ausentes |  | | |

|  |  |  |  |
| --- | --- | --- | --- |
| |  |  |  | | --- | --- | --- | | | GO:0016788 | hydrolase activity, acting on ester bonds | | --- | --- | | |

|  |  |  |  |
| --- | --- | --- | --- |
| |  |  |  | | --- | --- | --- | | | GO:0016817 | hydrolase activity, acting on acid anhydrides | | --- | --- | | |

|  |  |  |  |
| --- | --- | --- | --- |
| |  |  |  | | --- | --- | --- | | | GO:0016810 | hydrolase activity, acting on carbon nitrogen (but not peptide) bonds | | --- | --- | | |

|  |  |  |  |  |  |  |  |  |
| --- | --- | --- | --- | --- | --- | --- | --- | --- |
| |  |  |  |  |  |  |  |  | | --- | --- | --- | --- | --- | --- | --- | --- | | | GO:0016811 1:4|4:6306 2.54e-03 1:72|4:6306 4.49e-02 | hydrolase activity, acting on carbon nitrogen (but not peptide) bonds, in linear amides | | | | --- | --- | --- | --- | | QNS1 (YHR074W) | | Genes ausentes |  | | |

|  |  |  |  |  |  |  |  |  |
| --- | --- | --- | --- | --- | --- | --- | --- | --- |
| |  |  |  |  |  |  |  |  | | --- | --- | --- | --- | --- | --- | --- | --- | | | GO:0004661 1:2|2:6306 6.34e-04 1:72|2:6306 2.27e-02 | protein geranylgeranyltransferase activity | | | | --- | --- | --- | --- | | CDC43 (YGL155W) | | Genes ausentes |  | | |

|  |  |  |  |  |  |  |  |  |
| --- | --- | --- | --- | --- | --- | --- | --- | --- |
| |  |  |  |  |  |  |  |  | | --- | --- | --- | --- | --- | --- | --- | --- | | | GO:0045547 1:2|2:6306 6.34e-04 1:72|2:6306 2.27e-02 | dehydrodolichyl diphosphate synthase activity | | | | --- | --- | --- | --- | | RER2 (YBR002C) | | Genes ausentes |  | | |

|  |  |  |  |
| --- | --- | --- | --- |
| |  |  |  | | --- | --- | --- | | | GO:0008318 | protein prenyltransferase activity | | --- | --- | | |

|  |  |  |  |  |  |  |  |  |  |
| --- | --- | --- | --- | --- | --- | --- | --- | --- | --- |
| |  |  |  |  |  |  |  |  |  | | --- | --- | --- | --- | --- | --- | --- | --- | --- | | | GO:0004659 2:10|10:6306 1.01e-04 2:72|10:6306 5.45e-03 | prenyltransferase activity | | | | --- | --- | --- | --- | | RER2 (YBR002C) | | CDC43 (YGL155W) | | Genes ausentes |  | | |

|  |  |  |  |
| --- | --- | --- | --- |
| |  |  |  | | --- | --- | --- | | | GO:0016769 | transferase activity, transferring nitrogenous groups | | --- | --- | | |

|  |  |  |  |  |  |  |  |  |
| --- | --- | --- | --- | --- | --- | --- | --- | --- |
| |  |  |  |  |  |  |  |  | | --- | --- | --- | --- | --- | --- | --- | --- | | | GO:0016772 18:120|120:6306 6.37e-12 18:72|120:6306 5.14e-16 | transferase activity, transferring phosphorus containing groups | | | | --- | --- | --- | --- | | TOR2 (YKL203C) | | Genes ausentes |  | | |

|  |  |  |  |
| --- | --- | --- | --- |
| |  |  |  | | --- | --- | --- | | | GO:0016765 | transferase activity, transferring alkyl or aryl (other than methyl) groups | | --- | --- | | |

|  |  |  |  |  |  |  |  |  |
| --- | --- | --- | --- | --- | --- | --- | --- | --- |
| |  |  |  |  |  |  |  |  | | --- | --- | --- | --- | --- | --- | --- | --- | | | GO:0004662 1:2|2:6306 6.34e-04 1:72|2:6306 2.27e-02 | CAAX protein geranylgeranyltransferase activity | | | | --- | --- | --- | --- | | CDC43 (YGL155W) | | Genes ausentes |  | | |

|  |  |  |  |  |  |  |  |  |
| --- | --- | --- | --- | --- | --- | --- | --- | --- |
| |  |  |  |  |  |  |  |  | | --- | --- | --- | --- | --- | --- | --- | --- | | | GO:0004815 1:4|4:6306 2.54e-03 1:72|4:6306 4.49e-02 | aspartate tRNA ligase activity | | | | --- | --- | --- | --- | | DPS1 (YLL018C) | | Genes ausentes |  | | |

|  |  |  |  |  |  |  |  |  |
| --- | --- | --- | --- | --- | --- | --- | --- | --- |
| |  |  |  |  |  |  |  |  | | --- | --- | --- | --- | --- | --- | --- | --- | | | GO:0004830 1:2|2:6306 6.34e-04 1:72|2:6306 2.27e-02 | tryptophan tRNA ligase activity | | | | --- | --- | --- | --- | | WRS1 (YOL097C) | | Genes ausentes |  | | |

|  |  |  |  |  |  |  |  |  |
| --- | --- | --- | --- | --- | --- | --- | --- | --- |
| |  |  |  |  |  |  |  |  | | --- | --- | --- | --- | --- | --- | --- | --- | | | GO:0004829 1:2|2:6306 6.34e-04 1:72|2:6306 2.27e-02 | threonine tRNA ligase activity | | | | --- | --- | --- | --- | | THS1 (YIL078W) | | Genes ausentes |  | | |

|  |  |  |  |  |  |  |  |  |  |  |  |  |
| --- | --- | --- | --- | --- | --- | --- | --- | --- | --- | --- | --- | --- |
| |  |  |  |  |  |  |  |  |  |  |  |  | | --- | --- | --- | --- | --- | --- | --- | --- | --- | --- | --- | --- | | | GO:0004812 5:38|38:6306 2.63e-06 5:72|38:6306 6.31e-05 | aminoacyl tRNA ligase activity | | | | --- | --- | --- | --- | | GUS1 (YGL245W) | | WRS1 (YOL097C) | | THS1 (YIL078W) | | DPS1 (YLL018C) | | GLN4 (YOR168W) | | Genes ausentes |  | | |

|  |  |  |  |  |  |  |  |  |
| --- | --- | --- | --- | --- | --- | --- | --- | --- |
| |  |  |  |  |  |  |  |  | | --- | --- | --- | --- | --- | --- | --- | --- | | | GO:0016876 5:39|39:6306 3.43e-06 5:72|39:6306 7.18e-05 | ligase activity, forming aminoacyl tRNA and related compounds | | | | --- | --- | --- | --- | | THS1 (YIL078W) | | Genes ausentes |  | | |

|  |  |  |  |
| --- | --- | --- | --- |
| |  |  |  | | --- | --- | --- | | | GO:0016877 | ligase activity, forming carbon sulfur bonds | | --- | --- | | |

|  |  |  |  |
| --- | --- | --- | --- |
| |  |  |  | | --- | --- | --- | | | GO:0016875 | ligase activity, forming carbon oxygen bonds | | --- | --- | | |

|  |  |  |  |
| --- | --- | --- | --- |
| |  |  |  | | --- | --- | --- | | | GO:0016491 | oxidoreductase activity | | --- | --- | | |

|  |  |  |  |
| --- | --- | --- | --- |
| |  |  |  | | --- | --- | --- | | | GO:0004386 | helicase activity | | --- | --- | | |

|  |  |  |  |  |  |  |  |  |  |  |  |  |  |  |  |  |  |
| --- | --- | --- | --- | --- | --- | --- | --- | --- | --- | --- | --- | --- | --- | --- | --- | --- | --- |
| |  |  |  |  |  |  |  |  |  |  |  |  |  |  |  |  |  | | --- | --- | --- | --- | --- | --- | --- | --- | --- | --- | --- | --- | --- | --- | --- | --- | --- | | | GO:0016829 10:76|76:6306 1.78e-08 10:72|76:6306 1.04e-08 | lyase activity | | | | --- | --- | --- | --- | | ADE13 (YLR359W) | | CYR1 (YJL005W) | | ERG7 (YHR072W) | | FOL1 (YNL256W) | | HEM2 (YGL040C) | | FBA1 (YKL060C) | | HEM15 (YOR176W) | | HEM12 (YDR047W) | | MVD1 (YNR043W) | | HEM4 (YOR278W) | | Genes ausentes |  | | |

|  |  |  |  |
| --- | --- | --- | --- |
| |  |  |  | | --- | --- | --- | | | GO:0016787 | hydrolase activity | | --- | --- | | |

|  |  |  |  |
| --- | --- | --- | --- |
| |  |  |  | | --- | --- | --- | | | GO:0016740 | transferase activity | | --- | --- | | |

|  |  |  |  |
| --- | --- | --- | --- |
| |  |  |  | | --- | --- | --- | | | GO:0016874 | ligase activity | | --- | --- | | |

|  |  |  |  |
| --- | --- | --- | --- |
| |  |  |  | | --- | --- | --- | | | GO:0005488 | binding | | --- | --- | | |

|  |  |  |  |
| --- | --- | --- | --- |
| |  |  |  | | --- | --- | --- | | | GO:0003824 | catalytic activity | | --- | --- | | |

|  |  |  |  |
| --- | --- | --- | --- |
| |  |  |  | | --- | --- | --- | | | GO:0003674 | molecular\_function | | --- | --- | | |

|  |  |  |  |
| --- | --- | --- | --- |
| |  |  |  | | --- | --- | --- | | | GO:0003673 | Gene\_Ontology | | --- | --- | | |

|  |  |  |  |  |  |  |  |  |
| --- | --- | --- | --- | --- | --- | --- | --- | --- |
| |  |  |  |  |  |  |  |  | | --- | --- | --- | --- | --- | --- | --- | --- | | | GO:0004818 1:2|2:6306 6.34e-04 1:72|2:6306 2.27e-02 | glutamate tRNA ligase activity | | | | --- | --- | --- | --- | | GUS1 (YGL245W) | | Genes ausentes |  | | |
