# Supplementary material for: How to identify essential genes from molecular networks?
Source: BMC Syst Biol. 2009 Oct 13;3:102. doi: 10.1186/1752-0509-3-102 (PMC2765966; doi:10.1186/1752-0509-3-102)
Supplement: Additional file 2 — Figure S1. Unpredicted essential metabolic genes matching GO classification with locally essential genes. [file 1752-0509-3-102-S2.GZ › MF_local_unknown_with_coincidencias.html]

|  |  |
| --- | --- |
| |  | | --- | | Interacting selectizely with phosphopantetheine, the vitamin pantetheine 4 ,dihydrogen phosphate,. ChEBI:16858, GOC:mah | |


|  |  |  |  |  |  |  |  |  |
| --- | --- | --- | --- | --- | --- | --- | --- | --- |
| |  |  |  |  |  |  |  |  | | --- | --- | --- | --- | --- | --- | --- | --- | | | GO:0003984 1:2|2:6306 6.34e-04 1:20|2:6306 6.33e-03 | acetolactate synthase activity | | | | --- | --- | --- | --- | | ILV2 (YMR108W) | | Genes ausentes |  | | |

|  |  |  |  |  |  |  |  |  |  |
| --- | --- | --- | --- | --- | --- | --- | --- | --- | --- |
| |  |  |  |  |  |  |  |  |  | | --- | --- | --- | --- | --- | --- | --- | --- | --- | | | GO:0004321 2:2|2:6306 5.03e-08 2:20|2:6306 9.56e-06 | fatty acyl CoA synthase activity | | | | --- | --- | --- | --- | | FAS1 (YKL182W) | | FAS2 (YPL231W) | | Genes ausentes |  | | |

|  |  |  |  |
| --- | --- | --- | --- |
| |  |  |  | | --- | --- | --- | | | GO:0008375 | acetylglucosaminyltransferase activity | | --- | --- | | |

|  |  |  |  |
| --- | --- | --- | --- |
| |  |  |  | | --- | --- | --- | | | GO:0008194 | UDP glycosyltransferase activity | | --- | --- | | |

|  |  |  |  |
| --- | --- | --- | --- |
| |  |  |  | | --- | --- | --- | | | GO:0016758 | transferase activity, transferring hexosyl groups | | --- | --- | | |

|  |  |  |  |  |  |  |  |  |
| --- | --- | --- | --- | --- | --- | --- | --- | --- |
| |  |  |  |  |  |  |  |  | | --- | --- | --- | --- | --- | --- | --- | --- | | | GO:0004100 1:3|3:6306 1.43e-03 1:20|3:6306 9.49e-03 | chitin synthase activity | | | | --- | --- | --- | --- | | CHS2 (YBR038W) | | Genes ausentes |  | | |

|  |  |  |  |
| --- | --- | --- | --- |
| |  |  |  | | --- | --- | --- | | | GO:0016616 | oxidoreductase activity, acting on the CH OH group of donors, NAD or NADP as acceptor | | --- | --- | | |

|  |  |  |  |
| --- | --- | --- | --- |
| |  |  |  | | --- | --- | --- | | | GO:0016614 | oxidoreductase activity, acting on CH OH group of donors | | --- | --- | | |

|  |  |  |  |  |  |  |  |  |
| --- | --- | --- | --- | --- | --- | --- | --- | --- |
| |  |  |  |  |  |  |  |  | | --- | --- | --- | --- | --- | --- | --- | --- | | | GO:0004746 1:2|2:6306 6.34e-04 1:20|2:6306 6.33e-03 | riboflavin synthase activity | | | | --- | --- | --- | --- | | RIB5 (YBR256C) | | Genes ausentes |  | | |

|  |  |  |  |
| --- | --- | --- | --- |
| |  |  |  | | --- | --- | --- | | | GO:0004812 | aminoacyl tRNA ligase activity | | --- | --- | | |

|  |  |  |  |
| --- | --- | --- | --- |
| |  |  |  | | --- | --- | --- | | | GO:0016876 | ligase activity, forming aminoacyl tRNA and related compounds | | --- | --- | | |

|  |  |  |  |
| --- | --- | --- | --- |
| |  |  |  | | --- | --- | --- | | | GO:0016875 | ligase activity, forming carbon oxygen bonds | | --- | --- | | |

|  |  |  |  |  |  |  |  |  |
| --- | --- | --- | --- | --- | --- | --- | --- | --- |
| |  |  |  |  |  |  |  |  | | --- | --- | --- | --- | --- | --- | --- | --- | | | GO:0004825 1:2|2:6306 6.34e-04 1:20|2:6306 6.33e-03 | methionine tRNA ligase activity | | | | --- | --- | --- | --- | | MES1 (YGR264C) | | Genes ausentes |  | | |

|  |  |  |  |  |  |  |  |  |
| --- | --- | --- | --- | --- | --- | --- | --- | --- |
| |  |  |  |  |  |  |  |  | | --- | --- | --- | --- | --- | --- | --- | --- | | | GO:0008897 1:3|3:6306 1.43e-03 1:20|3:6306 9.49e-03 | phosphopantetheinyltransferase activity | | | | --- | --- | --- | --- | | FAS2 (YPL231W) | | Genes ausentes |  | | |

|  |  |  |  |
| --- | --- | --- | --- |
| |  |  |  | | --- | --- | --- | | | GO:0016780 | phosphotransferase activity, for other substituted phosphate groups | | --- | --- | | |

|  |  |  |  |
| --- | --- | --- | --- |
| |  |  |  | | --- | --- | --- | | | GO:0016779 | nucleotidyltransferase activity | | --- | --- | | |

|  |  |  |  |  |  |  |  |  |
| --- | --- | --- | --- | --- | --- | --- | --- | --- |
| |  |  |  |  |  |  |  |  | | --- | --- | --- | --- | --- | --- | --- | --- | | | GO:0004605 1:3|3:6306 1.43e-03 1:20|3:6306 9.49e-03 | phosphatidate cytidylyltransferase activity | | | | --- | --- | --- | --- | | CDS1 (YBR029C) | | Genes ausentes |  | | |

|  |  |  |  |
| --- | --- | --- | --- |
| |  |  |  | | --- | --- | --- | | | GO:0016419 | S malonyltransferase activity | | --- | --- | | |

|  |  |  |  |  |  |  |  |  |
| --- | --- | --- | --- | --- | --- | --- | --- | --- |
| |  |  |  |  |  |  |  |  | | --- | --- | --- | --- | --- | --- | --- | --- | | | GO:0050662 2:26|26:6306 5.00e-03 2:20|26:6306 2.97e-03 | coenzyme binding | | | | --- | --- | --- | --- | | ILV5 (YLR355C) | | Genes ausentes |  | | |

|  |  |  |  |
| --- | --- | --- | --- |
| |  |  |  | | --- | --- | --- | | | GO:0016408 | C acyltransferase activity | | --- | --- | | |

|  |  |  |  |
| --- | --- | --- | --- |
| |  |  |  | | --- | --- | --- | | | GO:0016417 | S acyltransferase activity | | --- | --- | | |

|  |  |  |  |
| --- | --- | --- | --- |
| |  |  |  | | --- | --- | --- | | | GO:0016420 | malonyltransferase activity | | --- | --- | | |

|  |  |  |  |
| --- | --- | --- | --- |
| |  |  |  | | --- | --- | --- | | | GO:0008415 | acyltransferase activity | | --- | --- | | |

|  |  |  |  |
| --- | --- | --- | --- |
| |  |  |  | | --- | --- | --- | | | GO:0016747 | transferase activity, transferring groups other than amino acyl groups | | --- | --- | | |

|  |  |  |  |
| --- | --- | --- | --- |
| |  |  |  | | --- | --- | --- | | | GO:0016744 | transferase activity, transferring aldehyde or ketonic groups | | --- | --- | | |

|  |  |  |  |
| --- | --- | --- | --- |
| |  |  |  | | --- | --- | --- | | | GO:0016757 | transferase activity, transferring glycosyl groups | | --- | --- | | |

|  |  |  |  |
| --- | --- | --- | --- |
| |  |  |  | | --- | --- | --- | | | GO:0016765 | transferase activity, transferring alkyl or aryl (other than methyl) groups | | --- | --- | | |

|  |  |  |  |
| --- | --- | --- | --- |
| |  |  |  | | --- | --- | --- | | | GO:0016772 | transferase activity, transferring phosphorus containing groups | | --- | --- | | |

|  |  |  |  |
| --- | --- | --- | --- |
| |  |  |  | | --- | --- | --- | | | GO:0016746 | transferase activity, transferring acyl groups | | --- | --- | | |

|  |  |  |  |  |  |  |  |  |
| --- | --- | --- | --- | --- | --- | --- | --- | --- |
| |  |  |  |  |  |  |  |  | | --- | --- | --- | --- | --- | --- | --- | --- | | | GO:0004315 1:2|2:6306 6.34e-04 1:20|2:6306 6.33e-03 | 3 oxoacyl acyl carrier protein synthase activity | | | | --- | --- | --- | --- | | FAS2 (YPL231W) | | Genes ausentes |  | | |

|  |  |  |  |  |  |  |  |  |
| --- | --- | --- | --- | --- | --- | --- | --- | --- |
| |  |  |  |  |  |  |  |  | | --- | --- | --- | --- | --- | --- | --- | --- | | | GO:0004316 1:2|2:6306 6.34e-04 1:20|2:6306 6.33e-03 | 3 oxoacyl acyl carrier protein reductase activity | | | | --- | --- | --- | --- | | FAS2 (YPL231W) | | Genes ausentes |  | | |

|  |  |  |  |  |  |  |  |  |
| --- | --- | --- | --- | --- | --- | --- | --- | --- |
| |  |  |  |  |  |  |  |  | | --- | --- | --- | --- | --- | --- | --- | --- | | | GO:0004314 1:2|2:6306 6.34e-04 1:20|2:6306 6.33e-03 | acyl carrier protein S malonyltransferase activity | | | | --- | --- | --- | --- | | FAS1 (YKL182W) | | Genes ausentes |  | | |

|  |  |  |  |  |  |  |  |  |
| --- | --- | --- | --- | --- | --- | --- | --- | --- |
| |  |  |  |  |  |  |  |  | | --- | --- | --- | --- | --- | --- | --- | --- | | | GO:0004312 2:5|5:6306 5.03e-06 2:20|5:6306 9.50e-05 | fatty acid synthase activity | | | | --- | --- | --- | --- | | FAS1 (YKL182W) | | Genes ausentes |  | | |

|  |  |  |  |
| --- | --- | --- | --- |
| |  |  |  | | --- | --- | --- | | | GO:0016866 | intramolecular transferase activity | | --- | --- | | |

|  |  |  |  |
| --- | --- | --- | --- |
| |  |  |  | | --- | --- | --- | | | GO:0016491 | oxidoreductase activity | | --- | --- | | |

|  |  |  |  |
| --- | --- | --- | --- |
| |  |  |  | | --- | --- | --- | | | GO:0016874 | ligase activity | | --- | --- | | |

|  |  |  |  |
| --- | --- | --- | --- |
| |  |  |  | | --- | --- | --- | | | GO:0016740 | transferase activity | | --- | --- | | |

|  |  |  |  |
| --- | --- | --- | --- |
| |  |  |  | | --- | --- | --- | | | GO:0016853 | isomerase activity | | --- | --- | | |

|  |  |  |  |  |  |  |  |  |
| --- | --- | --- | --- | --- | --- | --- | --- | --- |
| |  |  |  |  |  |  |  |  | | --- | --- | --- | --- | --- | --- | --- | --- | | | GO:0004619 1:5|5:6306 3.96e-03 1:20|5:6306 1.58e-02 | phosphoglycerate mutase activity | | | | --- | --- | --- | --- | | GPM1 (YKL152C) | | Genes ausentes |  | | |

|  |  |  |  |  |  |  |  |  |  |
| --- | --- | --- | --- | --- | --- | --- | --- | --- | --- |
| |  |  |  |  |  |  |  |  |  | | --- | --- | --- | --- | --- | --- | --- | --- | --- | | | GO:0016868 2:9|9:6306 6.49e-05 2:20|9:6306 3.40e-04 | intramolecular transferase activity, phosphotransferases | | | | --- | --- | --- | --- | | GPM1 (YKL152C) | | PCM1 (YEL058W) | | Genes ausentes |  | | |

|  |  |  |  |  |  |  |  |  |
| --- | --- | --- | --- | --- | --- | --- | --- | --- |
| |  |  |  |  |  |  |  |  | | --- | --- | --- | --- | --- | --- | --- | --- | | | GO:0030976 1:8|8:6306 1.01e-02 1:20|8:6306 2.51e-02 | thiamin pyrophosphate binding | | | | --- | --- | --- | --- | | ILV2 (YMR108W) | | Genes ausentes |  | | |

|  |  |  |  |
| --- | --- | --- | --- |
| |  |  |  | | --- | --- | --- | | | GO:0048037 | cofactor binding | | --- | --- | | |

|  |  |  |  |
| --- | --- | --- | --- |
| |  |  |  | | --- | --- | --- | | | GO:0019842 | vitamin binding | | --- | --- | | |

|  |  |  |  |
| --- | --- | --- | --- |
| |  |  |  | | --- | --- | --- | | | GO:0003824 | catalytic activity | | --- | --- | | |

|  |  |  |  |
| --- | --- | --- | --- |
| |  |  |  | | --- | --- | --- | | | GO:0005488 | binding | | --- | --- | | |

|  |  |  |  |
| --- | --- | --- | --- |
| |  |  |  | | --- | --- | --- | | | GO:0003674 | molecular\_function | | --- | --- | | |

|  |  |  |  |
| --- | --- | --- | --- |
| |  |  |  | | --- | --- | --- | | | GO:0003673 | Gene\_Ontology | | --- | --- | | |

|  |  |  |  |  |  |  |  |  |
| --- | --- | --- | --- | --- | --- | --- | --- | --- |
| |  |  |  |  |  |  |  |  | | --- | --- | --- | --- | --- | --- | --- | --- | | | GO:0031177 1:3|3:6306 1.43e-03 1:20|3:6306 9.49e-03 | phosphopantetheine binding | | | | --- | --- | --- | --- | | FAS2 (YPL231W) | | Genes ausentes |  | | |
