# Supplementary material for: How to identify essential genes from molecular networks?
Source: BMC Syst Biol. 2009 Oct 13;3:102. doi: 10.1186/1752-0509-3-102 (PMC2765966; doi:10.1186/1752-0509-3-102)
Supplement: Additional file 2 — Figure S1. Unpredicted essential metabolic genes matching GO classification with locally essential genes. [file 1752-0509-3-102-S2.GZ › MF_local_with_coincidencias.html]

|  |  |  |  |  |  |  |  |  |
| --- | --- | --- | --- | --- | --- | --- | --- | --- |
| |  |  |  |  |  |  |  |  | | --- | --- | --- | --- | --- | --- | --- | --- | | | GO:0008177 1:5|5:6306 3.96e-03 1:39|5:6306 3.06e-02 | succinate dehydrogenase (ubiquinone) activity | | | | --- | --- | --- | --- | | SDH3 (YKL141W) | | Genes ausentes |  | | |

|  |  |  |  |  |  |  |  |  |  |
| --- | --- | --- | --- | --- | --- | --- | --- | --- | --- |
| |  |  |  |  |  |  |  |  |  | | --- | --- | --- | --- | --- | --- | --- | --- | --- | | | GO:0050661 2:12|12:6306 2.17e-04 2:39|12:6306 2.37e-03 | NADP binding | | | | --- | --- | --- | --- | | DFR1 (YOR236W) | | RIB7 (YBR153W) | | Genes ausentes |  | | |

|  |  |  |  |  |  |  |  |  |
| --- | --- | --- | --- | --- | --- | --- | --- | --- |
| |  |  |  |  |  |  |  |  | | --- | --- | --- | --- | --- | --- | --- | --- | | | GO:0004326 1:3|3:6306 1.43e-03 1:39|3:6306 1.84e-02 | tetrahydrofolylpolyglutamate synthase activity | | | | --- | --- | --- | --- | | FOL3 (YMR113W) | | Genes ausentes |  | | |

|  |  |  |  |
| --- | --- | --- | --- |
| |  |  |  | | --- | --- | --- | | | GO:0019201 | nucleotide kinase activity | | --- | --- | | |

|  |  |  |  |  |  |  |  |  |
| --- | --- | --- | --- | --- | --- | --- | --- | --- |
| |  |  |  |  |  |  |  |  | | --- | --- | --- | --- | --- | --- | --- | --- | | | GO:0009041 1:2|2:6306 6.34e-04 1:39|2:6306 1.23e-02 | uridylate kinase activity | | | | --- | --- | --- | --- | | CDC8 (YJR057W) | | Genes ausentes |  | | |

|  |  |  |  |
| --- | --- | --- | --- |
| |  |  |  | | --- | --- | --- | | | GO:0050662 | coenzyme binding | | --- | --- | | |

|  |  |  |  |  |  |  |  |  |
| --- | --- | --- | --- | --- | --- | --- | --- | --- |
| |  |  |  |  |  |  |  |  | | --- | --- | --- | --- | --- | --- | --- | --- | | | GO:0048038 1:3|3:6306 1.43e-03 1:39|3:6306 1.84e-02 | quinone binding | | | | --- | --- | --- | --- | | SDH3 (YKL141W) | | Genes ausentes |  | | |

|  |  |  |  |  |  |  |  |  |
| --- | --- | --- | --- | --- | --- | --- | --- | --- |
| |  |  |  |  |  |  |  |  | | --- | --- | --- | --- | --- | --- | --- | --- | | | GO:0003983 1:2|2:6306 6.34e-04 1:39|2:6306 1.23e-02 | UTP:glucose 1 phosphate uridylyltransferase activity | | | | --- | --- | --- | --- | | UGP1 (YKL035W) | | Genes ausentes |  | | |

|  |  |  |  |
| --- | --- | --- | --- |
| |  |  |  | | --- | --- | --- | | | GO:0004674 | protein serine/threonine kinase activity | | --- | --- | | |

|  |  |  |  |
| --- | --- | --- | --- |
| |  |  |  | | --- | --- | --- | | | GO:0019205 | nucleobase, nucleoside, nucleotide kinase activity | | --- | --- | | |

|  |  |  |  |
| --- | --- | --- | --- |
| |  |  |  | | --- | --- | --- | | | GO:0004672 | protein kinase activity | | --- | --- | | |

|  |  |  |  |  |  |  |  |  |
| --- | --- | --- | --- | --- | --- | --- | --- | --- |
| |  |  |  |  |  |  |  |  | | --- | --- | --- | --- | --- | --- | --- | --- | | | GO:0008353 1:4|4:6306 2.54e-03 1:39|4:6306 2.45e-02 | RNA polymerase subunit kinase activity | | | | --- | --- | --- | --- | | CTK1 (YKL139W) | | Genes ausentes |  | | |

|  |  |  |  |  |  |  |  |  |
| --- | --- | --- | --- | --- | --- | --- | --- | --- |
| |  |  |  |  |  |  |  |  | | --- | --- | --- | --- | --- | --- | --- | --- | | | GO:0008650 1:2|2:6306 6.34e-04 1:39|2:6306 1.23e-02 | rRNA (uridine 2 O ) methyltransferase activity | | | | --- | --- | --- | --- | | SPB1 (YCL054W) | | Genes ausentes |  | | |

|  |  |  |  |
| --- | --- | --- | --- |
| |  |  |  | | --- | --- | --- | | | GO:0016668 | oxidoreductase activity, acting on sulfur group of donors, NAD or NADP as acceptor | | --- | --- | | |

|  |  |  |  |  |  |  |  |  |
| --- | --- | --- | --- | --- | --- | --- | --- | --- |
| |  |  |  |  |  |  |  |  | | --- | --- | --- | --- | --- | --- | --- | --- | | | GO:0004791 1:2|2:6306 6.34e-04 1:39|2:6306 1.23e-02 | thioredoxin disulfide reductase activity | | | | --- | --- | --- | --- | | TRR1 (YDR353W) | | Genes ausentes |  | | |

|  |  |  |  |
| --- | --- | --- | --- |
| |  |  |  | | --- | --- | --- | | | GO:0016454 | C palmitoyltransferase activity | | --- | --- | | |

|  |  |  |  |
| --- | --- | --- | --- |
| |  |  |  | | --- | --- | --- | | | GO:0016409 | palmitoyltransferase activity | | --- | --- | | |

|  |  |  |  |
| --- | --- | --- | --- |
| |  |  |  | | --- | --- | --- | | | GO:0016408 | C acyltransferase activity | | --- | --- | | |

|  |  |  |  |
| --- | --- | --- | --- |
| |  |  |  | | --- | --- | --- | | | GO:0008415 | acyltransferase activity | | --- | --- | | |

|  |  |  |  |
| --- | --- | --- | --- |
| |  |  |  | | --- | --- | --- | | | GO:0016747 | transferase activity, transferring groups other than amino acyl groups | | --- | --- | | |

|  |  |  |  |  |  |  |  |  |  |
| --- | --- | --- | --- | --- | --- | --- | --- | --- | --- |
| |  |  |  |  |  |  |  |  |  | | --- | --- | --- | --- | --- | --- | --- | --- | --- | | | GO:0004758 2:2|2:6306 5.03e-08 2:39|2:6306 3.73e-05 | serine C palmitoyltransferase activity | | | | --- | --- | --- | --- | | LCB1 (YMR296C) | | LCB2 (YDR062W) | | Genes ausentes |  | | |

|  |  |  |  |
| --- | --- | --- | --- |
| |  |  |  | | --- | --- | --- | | | GO:0016635 | oxidoreductase activity, acting on the CH CH group of donors, quinone or related compound as acceptor | | --- | --- | | |

|  |  |  |  |
| --- | --- | --- | --- |
| |  |  |  | | --- | --- | --- | | | GO:0016667 | oxidoreductase activity, acting on sulfur group of donors | | --- | --- | | |

|  |  |  |  |
| --- | --- | --- | --- |
| |  |  |  | | --- | --- | --- | | | GO:0009055 | electron carrier activity | | --- | --- | | |

|  |  |  |  |
| --- | --- | --- | --- |
| |  |  |  | | --- | --- | --- | | | GO:0016627 | oxidoreductase activity, acting on the CH CH group of donors | | --- | --- | | |

|  |  |  |  |  |  |  |  |  |
| --- | --- | --- | --- | --- | --- | --- | --- | --- |
| |  |  |  |  |  |  |  |  | | --- | --- | --- | --- | --- | --- | --- | --- | | | GO:0000104 1:4|4:6306 2.54e-03 1:39|4:6306 2.45e-02 | succinate dehydrogenase activity | | | | --- | --- | --- | --- | | SDH3 (YKL141W) | | Genes ausentes |  | | |

|  |  |  |  |
| --- | --- | --- | --- |
| |  |  |  | | --- | --- | --- | | | GO:0016436 | rRNA (uridine) methyltransferase activity | | --- | --- | | |

|  |  |  |  |
| --- | --- | --- | --- |
| |  |  |  | | --- | --- | --- | | | GO:0008649 | rRNA methyltransferase activity | | --- | --- | | |

|  |  |  |  |
| --- | --- | --- | --- |
| |  |  |  | | --- | --- | --- | | | GO:0008757 | S adenosylmethionine dependent methyltransferase activity | | --- | --- | | |

|  |  |  |  |
| --- | --- | --- | --- |
| |  |  |  | | --- | --- | --- | | | GO:0008173 | RNA methyltransferase activity | | --- | --- | | |

|  |  |  |  |
| --- | --- | --- | --- |
| |  |  |  | | --- | --- | --- | | | GO:0008168 | methyltransferase activity | | --- | --- | | |

|  |  |  |  |  |  |  |  |  |
| --- | --- | --- | --- | --- | --- | --- | --- | --- |
| |  |  |  |  |  |  |  |  | | --- | --- | --- | --- | --- | --- | --- | --- | | | GO:0016435 1:2|2:6306 6.34e-04 1:39|2:6306 1.23e-02 | rRNA (guanine) methyltransferase activity | | | | --- | --- | --- | --- | | SPB1 (YCL054W) | | Genes ausentes |  | | |

|  |  |  |  |
| --- | --- | --- | --- |
| |  |  |  | | --- | --- | --- | | | GO:0016421 | CoA carboxylase activity | | --- | --- | | |

|  |  |  |  |  |  |  |  |  |
| --- | --- | --- | --- | --- | --- | --- | --- | --- |
| |  |  |  |  |  |  |  |  | | --- | --- | --- | --- | --- | --- | --- | --- | | | GO:0003989 1:2|2:6306 6.34e-04 1:39|2:6306 1.23e-02 | acetyl CoA carboxylase activity | | | | --- | --- | --- | --- | | ACC1 (YNR016C) | | Genes ausentes |  | | |

|  |  |  |  |
| --- | --- | --- | --- |
| |  |  |  | | --- | --- | --- | | | GO:0016881 | acid amino acid ligase activity | | --- | --- | | |

|  |  |  |  |  |  |  |  |  |
| --- | --- | --- | --- | --- | --- | --- | --- | --- |
| |  |  |  |  |  |  |  |  | | --- | --- | --- | --- | --- | --- | --- | --- | | | GO:0004075 1:2|2:6306 6.34e-04 1:39|2:6306 1.23e-02 | biotin carboxylase activity | | | | --- | --- | --- | --- | | ACC1 (YNR016C) | | Genes ausentes |  | | |

|  |  |  |  |
| --- | --- | --- | --- |
| |  |  |  | | --- | --- | --- | | | GO:0051748 | UTP monosaccharide 1 phosphate uridylyltransferase activity | | --- | --- | | |

|  |  |  |  |
| --- | --- | --- | --- |
| |  |  |  | | --- | --- | --- | | | GO:0016776 | phosphotransferase activity, phosphate group as acceptor | | --- | --- | | |

|  |  |  |  |
| --- | --- | --- | --- |
| |  |  |  | | --- | --- | --- | | | GO:0016773 | phosphotransferase activity, alcohol group as acceptor | | --- | --- | | |

|  |  |  |  |
| --- | --- | --- | --- |
| |  |  |  | | --- | --- | --- | | | GO:0016301 | kinase activity | | --- | --- | | |

|  |  |  |  |  |  |  |  |  |
| --- | --- | --- | --- | --- | --- | --- | --- | --- |
| |  |  |  |  |  |  |  |  | | --- | --- | --- | --- | --- | --- | --- | --- | | | GO:0016780 1:5|5:6306 3.96e-03 1:39|5:6306 3.06e-02 | phosphotransferase activity, for other substituted phosphate groups | | | | --- | --- | --- | --- | | PIS1 (YPR113W) | | Genes ausentes |  | | |

|  |  |  |  |
| --- | --- | --- | --- |
| |  |  |  | | --- | --- | --- | | | GO:0016779 | nucleotidyltransferase activity | | --- | --- | | |

|  |  |  |  |  |  |  |  |  |  |
| --- | --- | --- | --- | --- | --- | --- | --- | --- | --- |
| |  |  |  |  |  |  |  |  |  | | --- | --- | --- | --- | --- | --- | --- | --- | --- | | | GO:0016769 2:11|11:6306 1.51e-04 2:39|11:6306 1.98e-03 | transferase activity, transferring nitrogenous groups | | | | --- | --- | --- | --- | | LCB1 (YMR296C) | | LCB2 (YDR062W) | | Genes ausentes |  | | |

|  |  |  |  |
| --- | --- | --- | --- |
| |  |  |  | | --- | --- | --- | | | GO:0016746 | transferase activity, transferring acyl groups | | --- | --- | | |

|  |  |  |  |
| --- | --- | --- | --- |
| |  |  |  | | --- | --- | --- | | | GO:0016741 | transferase activity, transferring one carbon groups | | --- | --- | | |

|  |  |  |  |
| --- | --- | --- | --- |
| |  |  |  | | --- | --- | --- | | | GO:0016772 | transferase activity, transferring phosphorus containing groups | | --- | --- | | |

|  |  |  |  |  |  |  |  |  |  |
| --- | --- | --- | --- | --- | --- | --- | --- | --- | --- |
| |  |  |  |  |  |  |  |  |  | | --- | --- | --- | --- | --- | --- | --- | --- | --- | | | GO:0003919 2:2|2:6306 5.03e-08 2:39|2:6306 3.73e-05 | FMN adenylyltransferase activity | | | | --- | --- | --- | --- | | FAD1 (YDL045C) | | FMN1 (YDR236C) | | Genes ausentes |  | | |

|  |  |  |  |  |  |  |  |  |  |
| --- | --- | --- | --- | --- | --- | --- | --- | --- | --- |
| |  |  |  |  |  |  |  |  |  | | --- | --- | --- | --- | --- | --- | --- | --- | --- | | | GO:0004826 2:3|3:6306 4.53e-07 2:39|3:6306 1.11e-04 | phenylalanine tRNA ligase activity | | | | --- | --- | --- | --- | | FRS1 (YLR060W) | | FRS2 (YFL022C) | | Genes ausentes |  | | |

|  |  |  |  |  |  |  |  |  |
| --- | --- | --- | --- | --- | --- | --- | --- | --- |
| |  |  |  |  |  |  |  |  | | --- | --- | --- | --- | --- | --- | --- | --- | | | GO:0004828 1:2|2:6306 6.34e-04 1:39|2:6306 1.23e-02 | serine tRNA ligase activity | | | | --- | --- | --- | --- | | SES1 (YDR023W) | | Genes ausentes |  | | |

|  |  |  |  |  |  |  |  |  |
| --- | --- | --- | --- | --- | --- | --- | --- | --- |
| |  |  |  |  |  |  |  |  | | --- | --- | --- | --- | --- | --- | --- | --- | | | GO:0004815 1:4|4:6306 2.54e-03 1:39|4:6306 2.45e-02 | aspartate tRNA ligase activity | | | | --- | --- | --- | --- | | DED81 (YHR019C) | | Genes ausentes |  | | |

|  |  |  |  |  |  |  |  |  |
| --- | --- | --- | --- | --- | --- | --- | --- | --- |
| |  |  |  |  |  |  |  |  | | --- | --- | --- | --- | --- | --- | --- | --- | | | GO:0004822 1:2|2:6306 6.34e-04 1:39|2:6306 1.23e-02 | isoleucine tRNA ligase activity | | | | --- | --- | --- | --- | | ILS1 (YBL076C) | | Genes ausentes |  | | |

|  |  |  |  |  |  |  |  |  |
| --- | --- | --- | --- | --- | --- | --- | --- | --- |
| |  |  |  |  |  |  |  |  | | --- | --- | --- | --- | --- | --- | --- | --- | | | GO:0004820 1:2|2:6306 6.34e-04 1:39|2:6306 1.23e-02 | glycine tRNA ligase activity | | | | --- | --- | --- | --- | | GRS1 (YBR121C) | | Genes ausentes |  | | |

|  |  |  |  |  |  |  |  |  |
| --- | --- | --- | --- | --- | --- | --- | --- | --- |
| |  |  |  |  |  |  |  |  | | --- | --- | --- | --- | --- | --- | --- | --- | | | GO:0004824 1:2|2:6306 6.34e-04 1:39|2:6306 1.23e-02 | lysine tRNA ligase activity | | | | --- | --- | --- | --- | | KRS1 (YDR037W) | | Genes ausentes |  | | |

|  |  |  |  |  |  |  |  |  |
| --- | --- | --- | --- | --- | --- | --- | --- | --- |
| |  |  |  |  |  |  |  |  | | --- | --- | --- | --- | --- | --- | --- | --- | | | GO:0004814 1:2|2:6306 6.34e-04 1:39|2:6306 1.23e-02 | arginine tRNA ligase activity | | | | --- | --- | --- | --- | | YDR341C (YDR341C) | | Genes ausentes |  | | |

|  |  |  |  |  |  |  |  |  |
| --- | --- | --- | --- | --- | --- | --- | --- | --- |
| |  |  |  |  |  |  |  |  | | --- | --- | --- | --- | --- | --- | --- | --- | | | GO:0004816 1:2|2:6306 6.34e-04 1:39|2:6306 1.23e-02 | asparagine tRNA ligase activity | | | | --- | --- | --- | --- | | DED81 (YHR019C) | | Genes ausentes |  | | |

|  |  |  |  |  |  |  |  |  |
| --- | --- | --- | --- | --- | --- | --- | --- | --- |
| |  |  |  |  |  |  |  |  | | --- | --- | --- | --- | --- | --- | --- | --- | | | GO:0004831 1:2|2:6306 6.34e-04 1:39|2:6306 1.23e-02 | tyrosine tRNA ligase activity | | | | --- | --- | --- | --- | | TYS1 (YGR185C) | | Genes ausentes |  | | |

|  |  |  |  |  |  |  |  |  |  |  |  |  |  |  |  |  |  |  |  |  |
| --- | --- | --- | --- | --- | --- | --- | --- | --- | --- | --- | --- | --- | --- | --- | --- | --- | --- | --- | --- | --- |
| |  |  |  |  |  |  |  |  |  |  |  |  |  |  |  |  |  |  |  |  | | --- | --- | --- | --- | --- | --- | --- | --- | --- | --- | --- | --- | --- | --- | --- | --- | --- | --- | --- | --- | | | GO:0004812 13:38|38:6306 6.76e-21 13:39|38:6306 1.01e-20 | aminoacyl tRNA ligase activity | | | | --- | --- | --- | --- | | ILS1 (YBL076C) | | FRS1 (YLR060W) | | HTS1 (YPR033C) | | CDC60 (YPL160W) | | FRS2 (YFL022C) | | ALA1 (YOR335C) | | SES1 (YDR023W) | | TYS1 (YGR185C) | | DED81 (YHR019C) | | VAS1 (YGR094W) | | YDR341C (YDR341C) | | GRS1 (YBR121C) | | KRS1 (YDR037W) | | Genes ausentes |  | | |

|  |  |  |  |  |  |  |  |  |
| --- | --- | --- | --- | --- | --- | --- | --- | --- |
| |  |  |  |  |  |  |  |  | | --- | --- | --- | --- | --- | --- | --- | --- | | | GO:0016876 13:39|39:6306 1.51e-20 13:39|39:6306 1.51e-20 | ligase activity, forming aminoacyl tRNA and related compounds | | | | --- | --- | --- | --- | | ALA1 (YOR335C) | | Genes ausentes |  | | |

|  |  |  |  |
| --- | --- | --- | --- |
| |  |  |  | | --- | --- | --- | | | GO:0016885 | ligase activity, forming carbon carbon bonds | | --- | --- | | |

|  |  |  |  |
| --- | --- | --- | --- |
| |  |  |  | | --- | --- | --- | | | GO:0016879 | ligase activity, forming carbon nitrogen bonds | | --- | --- | | |

|  |  |  |  |
| --- | --- | --- | --- |
| |  |  |  | | --- | --- | --- | | | GO:0016875 | ligase activity, forming carbon oxygen bonds | | --- | --- | | |

|  |  |  |  |
| --- | --- | --- | --- |
| |  |  |  | | --- | --- | --- | | | GO:0016491 | oxidoreductase activity | | --- | --- | | |

|  |  |  |  |
| --- | --- | --- | --- |
| |  |  |  | | --- | --- | --- | | | GO:0016740 | transferase activity | | --- | --- | | |

|  |  |  |  |  |  |  |  |  |  |  |  |  |  |  |  |  |  |  |  |  |  |  |
| --- | --- | --- | --- | --- | --- | --- | --- | --- | --- | --- | --- | --- | --- | --- | --- | --- | --- | --- | --- | --- | --- | --- |
| |  |  |  |  |  |  |  |  |  |  |  |  |  |  |  |  |  |  |  |  |  |  | | --- | --- | --- | --- | --- | --- | --- | --- | --- | --- | --- | --- | --- | --- | --- | --- | --- | --- | --- | --- | --- | --- | | | GO:0016874 15:120|120:6306 5.69e-09 15:39|120:6306 1.09e-16 | ligase activity | | | | --- | --- | --- | --- | | ILS1 (YBL076C) | | FRS1 (YLR060W) | | HTS1 (YPR033C) | | CDC60 (YPL160W) | | FRS2 (YFL022C) | | ALA1 (YOR335C) | | SES1 (YDR023W) | | TYS1 (YGR185C) | | DED81 (YHR019C) | | VAS1 (YGR094W) | | YDR341C (YDR341C) | | ACC1 (YNR016C) | | GRS1 (YBR121C) | | FOL3 (YMR113W) | | KRS1 (YDR037W) | | Genes ausentes |  | | |

|  |  |  |  |  |  |  |  |  |
| --- | --- | --- | --- | --- | --- | --- | --- | --- |
| |  |  |  |  |  |  |  |  | | --- | --- | --- | --- | --- | --- | --- | --- | | | GO:0004823 1:2|2:6306 6.34e-04 1:39|2:6306 1.23e-02 | leucine tRNA ligase activity | | | | --- | --- | --- | --- | | CDC60 (YPL160W) | | Genes ausentes |  | | |

|  |  |  |  |  |  |  |  |  |
| --- | --- | --- | --- | --- | --- | --- | --- | --- |
| |  |  |  |  |  |  |  |  | | --- | --- | --- | --- | --- | --- | --- | --- | | | GO:0009374 1:5|5:6306 3.96e-03 1:39|5:6306 3.06e-02 | biotin binding | | | | --- | --- | --- | --- | | ACC1 (YNR016C) | | Genes ausentes |  | | |

|  |  |  |  |
| --- | --- | --- | --- |
| |  |  |  | | --- | --- | --- | | | GO:0048037 | cofactor binding | | --- | --- | | |

|  |  |  |  |
| --- | --- | --- | --- |
| |  |  |  | | --- | --- | --- | | | GO:0019842 | vitamin binding | | --- | --- | | |

|  |  |  |  |
| --- | --- | --- | --- |
| |  |  |  | | --- | --- | --- | | | GO:0016209 | antioxidant activity | | --- | --- | | |

|  |  |  |  |
| --- | --- | --- | --- |
| |  |  |  | | --- | --- | --- | | | GO:0003824 | catalytic activity | | --- | --- | | |

|  |  |  |  |
| --- | --- | --- | --- |
| |  |  |  | | --- | --- | --- | | | GO:0005488 | binding | | --- | --- | | |

|  |  |  |  |
| --- | --- | --- | --- |
| |  |  |  | | --- | --- | --- | | | GO:0003674 | molecular\_function | | --- | --- | | |

|  |  |  |  |
| --- | --- | --- | --- |
| |  |  |  | | --- | --- | --- | | | GO:0003673 | Gene\_Ontology | | --- | --- | | |

|  |  |  |  |  |  |  |  |  |
| --- | --- | --- | --- | --- | --- | --- | --- | --- |
| |  |  |  |  |  |  |  |  | | --- | --- | --- | --- | --- | --- | --- | --- | | | GO:0031177 1:3|3:6306 1.43e-03 1:39|3:6306 1.84e-02 | phosphopantetheine binding | | | | --- | --- | --- | --- | | ACP1 (YKL192C) | | Genes ausentes |  | | |
